# Quantitative iTRAQ LC-MS/MS Proteomics Reveals the Proteome Profiles of DF-1 Cells after Infection with Subgroup J Avian Leukosis Virus

**DOI:** 10.1155/2015/395307

**Published:** 2015-01-08

**Authors:** Xiaofei Li, Qi Wang, Yanni Gao, Xiaole Qi, Yongqiang Wang, Honglei Gao, Yulong Gao, Xiaomei Wang

**Affiliations:** ^1^Division of Avian Infectious Diseases, State Key Laboratory of Veterinary Biotechnology, Harbin Veterinary Research Institute, Chinese Academy of Agricultural Sciences, Harbin 150001, China; ^2^Jiangsu Co-Innovation Center for Prevention and Control of Important Animal Infectious Disease and Zoonoses, Yangzhou 225009, China

## Abstract

Avian leukosis virus subgroup J (ALV-J) is an avian oncogenic retrovirus that can induce various clinical tumors and has caused severe economic losses in China. To improve our understanding of the host cellular responses to virus infection and the pathogenesis of ALV-J infection, we applied isobaric tags for relative and absolute quantification (iTRAQ) labeling coupled with multidimensional liquid chromatography-tandem mass spectrometry to detect the protein changes in DF-1 cells infected and mock-infected with ALV-J. A total of 75 cellular proteins were significantly changed, including 33 upregulated proteins and 42 downregulated proteins. The reliability of iTRAQ-LC MS/MS was confirmed via real-time PCR. Most of these proteins were related to the physiological functions of metabolic processes, biosynthetic processes, responses to stimuli, protein binding, signal transduction, cell cytoskeleton, and so forth. We also found some proteins that play important roles in apoptosis and oncogenicity. The differentially expressed proteins identified may provide valuable information to elucidate the pathogenesis of virus infection and virus-host interactions.

## 1. Introduction

The J subgroup of avian leukosis virus (ALV-J), which belongs to the Retroviridae family, was first isolated from white-meat-type chickens in the United Kingdom in 1988 [1]. It can predominantly lead to myeloid leukosis (ML) and immunosuppression effects in both naturally and experimentally infected chickens [2, 3]. In China, ALV-J-associated myeloid leukosis in chickens was first reported in 1999 [4]. ALV-J can induce various tumors, growth retardation, and production problems. In addition, in recent years, it has become widespread in many parts of our country and leads to severe economic losses in the poultry industry.

The pathogenesis of virus infection and the mechanism through which the virus interacts with host cells remain unclear. During virus infection, the proteins of host cells may be significantly changed. It is now possible to use proteomic techniques to identify the changes in protein abundance that indicate host cellular responses to virus infection and provide useful information to obtain a better understanding of the pathogenesis of virus infection [5–8]. Kvaratskhelia et al. [9] applied enzymatic digestion coupled with mass spectrometry (MS) to detect the sites of glycosylation on the surface of avian leukosis virus subgroup A (ALV-A) and found that carbohydrates may play an important role in receptor binding.

To explore the possible mechanisms of virus infection, we used isobaric tags for relative and absolute quantification (iTRAQ) combined with multidimensional liquid chromatography (LC) and tandem MS analysis to perform a quantitative proteomic analysis of DF-1 cells infected with ALV-J [10]. To the best of our knowledge, no previous study had used the iTRAQ LC-MS/MS proteomics strategy to investigate the differently expressed proteins in ALV-J-infected DF-1 cells. The iTRAQ labeling technology could greatly increase the identification sensitivity and quantitation accuracy of proteomic analyses through a multiplexed quantitation strategy [11]. The results showed that 75 proteins were significantly changed after ALV-J infection. These changed proteins may provide valuable information to study the molecular mechanisms underlying ALV-J pathogenesis.

## 2. Materials and Methods

### 2.1. Reagents

The iTRAQ Reagent Multi-Plex Kit was acquired from Applied Biosystems (Foster City, CA, USA). A multidimensional liquid chromatographer (RIGOL 3220) was purchased from RIGOL, and the chromatographic column (Agela, C18 chromatographic column, 250 × 4.6 mm i.d., filler particles diameter: 5 *μ*m) was acquired from Agela Co., Ltd. (Tianjin, China). The LC-MS/MS instrument (Q-Exactive) was obtained from Thermo Fisher Scientific.

### 2.2. Cell Culture and Virus Infection

DF-1 cells (ATCC accession number: CRL-12203) were cultured in Dulbecco's modified Eagle medium (DMEM; HyClone, Beijing, China) supplemented with 10% fetal bovine serum (FBS) and 100 *μ*g/mL streptomycin and penicillin at 37°C in a 5% CO_2_ atmosphere. ALV-J strain HPRS-103 (GenBank: Z46390) was kindly provided by Professor Venugopal Nair. DF-1 cells cultured in flasks to approximately 80% confluence were infected with 0.5 mL of 10^3.5^/mL 50% tissue culture infectious doses (TCID50) of ALV-J for 144 h. Uninfected DF-1 cells served as mock-infected cells.

### 2.3. Indirect Immune Fluorescence Assay (IFA)

At 144 h after infection, the infected DF-1 cells were washed twice with PBS and fixed with anhydrous ethanol for 20 min. The fixed cells were then incubated with mouse anti-P27 monoclonal antibody (prepared in our lab) at 37°C for 60 min. After washing three times with PBST (0.01 M PBS, pH 7.2, 0.05% Tween 20), the cells were incubated with goat anti-mouse IgG conjugated to FITC (Sigma, USA) at 37°C for another 60 min. Finally, the cells were observed under a Carl Zeiss Vision microscope (ZEISS Axio Observer D1) after three washes with PBST.

### 2.4. Protein Extraction, Digestion, and Labeling with iTRAQ Reagents

Infected and mock-infected DF-1 cells were washed twice with PBS. The cells were lysed in a lysis buffer (9 M urea, 4% CHAPS, 1% DTT, and 1% IPG buffer). The mixtures were centrifuged at 15,000 g and 4°C for 15 min. The supernatant was collected, and the protein concentration was determined using the Bradford protein assay [12] (Bio-Rad Laboratories). Then, 100 *μ*g of protein was mixed overnight with four volumes of cold (−20°C) acetone and then dissolved using the dissolution buffer. After being reduced, alkylated, and digested with trypsin, the samples were labeled following the manufacturer's instructions described in the iTRAQ protocol. The labeled samples were pooled for further analysis.

### 2.5. LC-MS/MS and Database Searches

The iTRAQ-labeled sample mixtures were then fractionated by strong cation exchange (SCX) chromatography on a high-performance liquid chromatography (HPLC) system (RIGOL 3220; Beijing, China) using a chromatographic column (Agela, C18 chromatographic column, 250 × 4.6 mm i.d., filler particles diameter: 5 *μ*m; Tianjin, China). Mobile phase A consisted of 2% ACN-98% H_2_O (pH 10.0), and mobile phase B consisted of 98% ACN-2% H_2_O (pH 10.0). The solvent gradient was as follows: 5%–8% B for 1 min, 8%–32% B for 24 min, 32%–95% B for 2 min, 95% for 4 min, and 95%–5% B for 1 min. The column temperature was 45°C, the flow rate was 0.7 mL/min, and the detection wavelength was 214 nm. Peptides were collected every minute within the effective gradient from 8% to 32%. A total of 27 fractions were collected and then dried.

The dried fractions were dissolved in 1.9% ACN/98% H_2_O/0.1% FA aqueous solution and combined into nine samples. The samples were centrifuged at 12,000 ×r for 3 min, and the supernatant was collected. The supernatant was then analyzed using the EASY-nLC-1000 liquid phase interfaced with a Q Exactive mass spectrometer (Thermo Fisher). The chromatographic conditions are as follows: liquid phase, EASY-nLC-1000; enriching column, C18, 5 *μ*m, ID100 *μ*m, 20 mm in length; separation column, C18, 3 *μ*m, ID75 *μ*m, 120 mm in length; mobile phase A, 1.9% ACN + 98% H_2_O + 0.1% FA; mobile phase B, 98% ACN + 1.9% H_2_O + 0.1% FA; and flow rate, 450 nl/min.

*Elution Conditions.*  See Table 1.

The data were acquired at 38 min. The spray voltage was 2.0 KV, the capillary temperature was 320°C, the collision energy was 30, and the acquisition quality range was 300–1400 da.

The relative quantification and protein identification were performed with the Protein Discoverer software (version 1.2) using the built-in mascot as the search engine.

### 2.6. Real-Time PCR

The primers (Table 2) were synthesized by BoShi Biotechnology Company (Harbin, China). The gene was amplified from the genomic DNA of DF-1 cells by polymerase chain reaction (PCR). The PCR-amplified products were separated in a 2% agarose gel and then purified using a DNA gel extraction kit (Axygen Biotechnology Limited, Hangzhou City, China). The products were then ligated into the pZeroBack/blunt vector (Tiangen Biotech Co., Ltd., Beijing, China), and the sequence was verified. The plasmid DNA was used as the standard to construct the standard curve via SYBR Green real-time PCR. The total cellular RNA of the infected or mock-infected DF-1 cells was extracted using the RNeasy Mini Kit (QIAGEN, China) according to the manufacturer's protocol. Reverse transcription was performed using a PrimeScript II First-Strand cDNA Synthesis Kit (TaKaRa, China) as described in the protocol. The real-time PCR was performed using the Roche LightCycler 480 real-time PCR System.

### 2.7. Bioinformatics Analysis

The functional annotation of the 75 proteins in DF-1 cells that were significantly changed after infection with ALV-J was performed using the GOSlimViewer tool of the AgBase database (http://www.agbase.msstate.edu/) [13]. In addition, we aimed to determine how ALV-J interacts with the host cellular proteins and how it affects the function of host cells. The identified proteins were inputted into the STRING database to obtain the protein-protein interaction network [14, 15] (http://string.embl.de/).

## 3. Results

### 3.1. Confirmation of ALV-J Infection in DF-1 Cells by IFA

**Figure 1:**
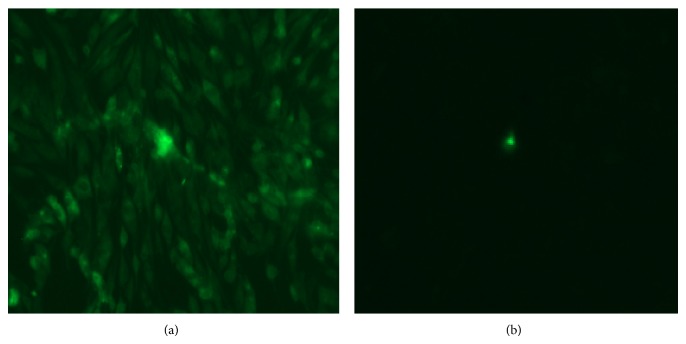
Identification of DF-1 cells infected with ALV-J by IFA. (a) DF1 cells infected with ALV-B. (b) Normal uninfected DF1 cells.

To confirm that the DF-1 cells were infected by ALV-J, IFA was used to detect the viral P27 antigen. The results showed clear green fluorescence in ALV-J-infected DF-1 cells 144 h after infection, whereas the uninfected DF-1 cells exhibited no green fluorescence (Figure 1).

### 3.2. Protein Profile Obtained by iTRAQ LC-MS/MS Analysis

To explore the differences in the protein expression levels after virus infection, the total proteins of ALV-J-infected and mock-infected DF-1 cells were extracted for iTRAQ-LC-MS/MS analysis. A total of 1091 proteins were detected, including 75 proteins in DF-1 cells that were significantly changed infection with ALV-J for 144 h (Table 3). These differently expressed proteins were divided into two clusters: upregulated and downregulated. The number of upregulated proteins was 33, whereas the number of downregulated proteins was 42.

### 3.3. Functional Classifications of the Identified Proteins

To annotate the functions of the 75 significantly changed proteins identified in our study, the proteins were submitted to GORetriever (http://www.agbase.msstate.edu/) for analysis. Three types of annotations were obtained using the website: molecular functions, biological processes, and cellular components.

**Figure 2:**
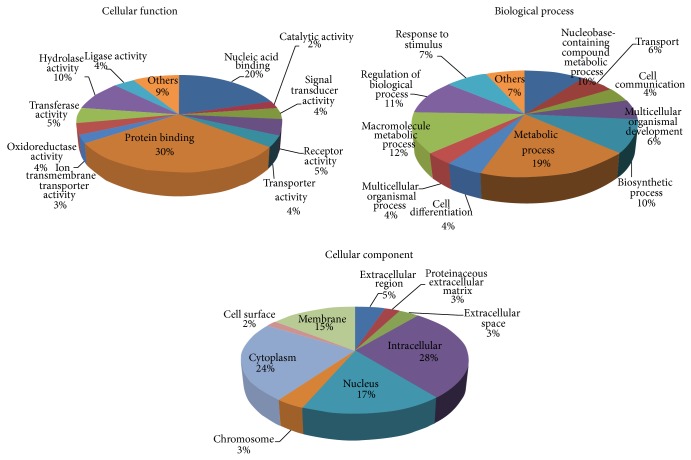
Functional annotation of the differently expressed proteins according to their biological process, molecular function, and cellular component.

The biological process annotation revealed that the significantly changed proteins were involved in metabolic process (19%), macromolecule metabolic process (12%), regulation of biological process (11%), biosynthetic processes (10%), nucleobase-containing compound metabolic process (10%), response to stimulus (7%), and various other activities (31%) (Figure 2, biological process).

The molecular function annotation revealed that these differently expressed proteins were involved in protein binding (30%), nucleic acid binding (21%), hydrolase activity (11%), transferase activity (5%), receptor activity (5%), oxidoreductase activity (4%), and various other activities (24%) (Figure 2, molecular function).

The cellular component annotation revealed that the altered proteins were associated with the following cellular components: intracellular (28%), cytoplasm (24%), nucleus (17%), membrane (15%), extracellular region (5%), chromosome (3%), and various others (8%) (Figure 2, cellular component).

### 3.4. Validation of the iTRAQ Data by Real-Time PCR

**Figure 3:**
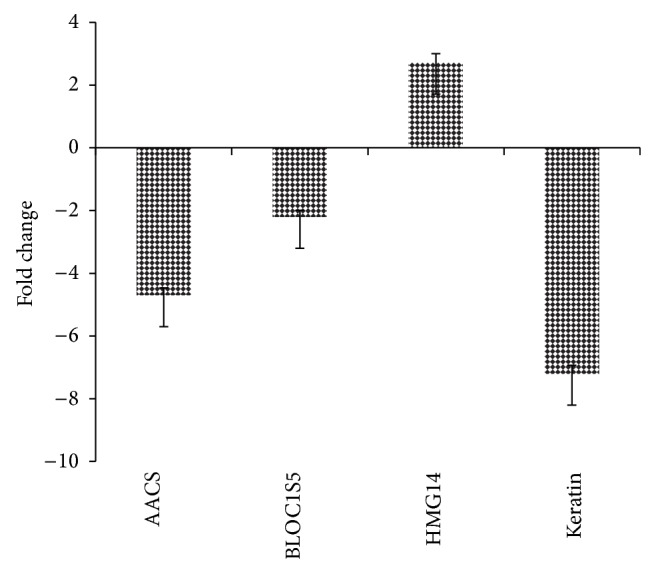
Transcriptional profiles of the significantly changed proteins in ALV-J-infected DF-1 cells. The error bars represent the standard deviations.

To confirm the results of the differentially expressed proteins identified by iTRAQ LC-MS/MS analysis, real-time PCR was performed to detect the transcript expression levels of the genes after ALV-J infection. We generated four standard curves to determine the gene expression of BLOC1S5, keratin, HMG14, and AACS in ALV-J-infected and mock-infected DF-1 cells. The results showed that HMG14 was upregulated (Figure 3), whereas BLOC1S5, AACS, and keratin were downregulated (Figure 3). The RT-PCR results were consistent with the results of the iTRAQ LC-MS/MS analysis (Table 3), confirming that the iTRAQ data were reliable.

### 3.5. Protein-Protein Interaction Analysis

**Figure 4:**
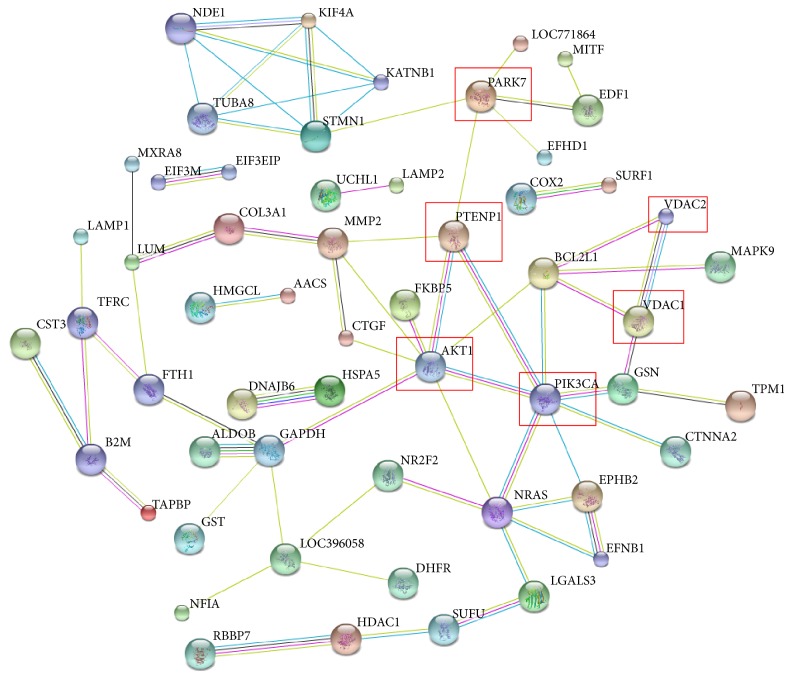
The protein-protein interaction between the identified proteins and the tumor- or apoptosis-associated proteins analyzed by the STRING software. An edge was drawn with up to seven differently colored lines, representing the existence of the seven types of evidence used for predicting the associations: a red line indicates the presence of fusion evidence; a yellow line indicates text mining evidence; a purple line indicates experimental evidence; a blue line indicates cooccurrence evidence; a light blue line indicates database evidence; a green line indicates neighborhood evidence; a black line indicates coexpression evidence.

The mechanism through which the virus interacts with host cells remains unclear, and oncogenicity is an important index of the pathogenicity of ALV-J. During virus infection, some proteins of host cells may be significantly changed. As a result, the functions of the changed proteins will also be altered. In our study, we aimed to determine whether the significantly changed proteins that were identified have some relationship with apoptosis or ALV-J-induced oncogenicity. We searched the STRING database to analyze the protein-protein interactions between the differently expressed proteins and PARK7, PTENP1, AKT1, PIK3CA (PI3K), and VDAC (Figure 4). These proteins are known to have some relationship with tumor-associated process and apoptosis. The protein-protein interaction networks may provide valuable information to further investigate the possible mechanism of ALV-J-induced oncogenicity.

## 4. Discussion

Proteomics is a relatively novel technology that has been used for the detection of the host cellular proteins response to virus infection [16, 17]. Isobaric tags for relative and absolute quantification (iTRAQ) combined with multidimensional liquid chromatography (LC) and tandem MS analysis are a powerful tool for quantitative proteomic analysis that has been widely applied in many studies [18–20]. In this study, we first applied the iTRAQ approach to identify the differential protein expression profiles of DF-1 cells infected with ALV-J. Using the iTRAQ LC-MS/MS technology, the significantly changed proteins were mostly associated with metabolic process, signal transducer activity, cell cytoskeleton, oxidoreductase activity, response to stimulus, and immune responses. In addition, some apoptosis and tumor-associated proteins (VEGF-A, ACTN4, and METAP2) were also identified by the iTRAQ LC-MS/MS technology.

### 4.1. Alterations of Tumor-Associated Proteins

Vascular endothelial growth factor A (VEGF-A) is an important inducer of angiogenesis [21]. As has been shown in many reports, upregulated VEGF-A can induce tumor formation via some unique signaling pathways [22, 23]. In addition, VEGF-A, which is known as a positive regulator, contributes to tumor growth and promotes tumor formation [24, 25]. Previous studies described a threshold level of proteins to promote tumorigenesis, which indicated that the expression level of one protein needs to reach the threshold level before promoting tumorigenesis [26, 27]. Studies in our lab showed that the increased replication of ALV-J increased the expression of VEGF-A, indicating an increased opportunity for ALV-J to push the expression level of VEGF-A to reach the threshold level to promote tumorigenesis [27]. In this study, we found that VEGF-A is overexpressed in DF-1 cells after infection with ALV-J. The results further suggested that VEGF-A is closely associated with ALV-J-induced tumorigenesis and may also suggest a novel molecular mechanism for better understanding of the higher oncogenicity of ALV-J.

Alpha-actinins (ACTNs) were classified into cytoskeleton proteins, while ACTN4 has some other unique functions, such as signal transduction, protein expression regulation, and nuclear transport. Histological analyses of cancer tissues showed a strong correlation between ACTN4 expression and tumorigenesis in several types of cancers [28–30]. Furthermore, upregulated ACTN4 in cancer cells has been suggested as a biomarker for drug resistance and malignant cell invasion [31–34]. Previous studies showed that ALV-J infection in DF-1 cells led to rapid increase in Akt phosphorylation and the phosphorylation of Akt was PI3K-dependent [35]. PI3K/Akt pathway also regulates viral replication of ALV-J [35]. Furthermore, AKT interacts with ACTN4 and ACTN4 is a functional partner of AKT [36]. Therefore, the upregulated ACTN4 observed in this study may be associated with tumorigenesis induced by ALV-J through PI3K/Akt pathway. This may provide useful information to elucidate the mechanism of ALV-J induced tumorigenesis and may also become potential therapeutic targets to control ALV-J infections.

METAP2 was considered to have some relationship with angiogenesis inhibition [37]. In addition, METAP2 can block B cell differentiation into plasma cells [38]. Some viruses, whose primary target cells are B cells, can clinically induce tumor formation. Therefore, downregulation of METAP2 in this study may influence the function of B cells, which may provide evidence to explain why ALV-J infection can result in immune suppression and tumorigenesis.

### 4.2. Redox Regulation

Peroxiredoxins (PRDXs), a family of peroxidases as antioxidant enzymes, can support tumor maintenance and survival through protecting cells from apoptosis induced by oxidative stress [39–41]. A previous study indicated that liver cells transfected with PRDX6 siRNA resulted in an increase in peroxide-induced cytotoxicity by apoptosis, which implies that decrease of PRDX6 promotes apoptosis [42]. Therefore, downregulated PRDX6 in this study suggests that ALV-J infection may weaken the antiapoptotic function of PRDX6.

In addition, PRDX1 was found to be upregulated in this study. A previous study indicated that the mice lacking PRDX1 have several malignant cancers, including sarcomas, carcinomas, and lymphomas [43]. These malignancies are associated with low expression of PRDX1, which suggests that PRDX1 may function as a tumor suppressor [43]. Studies also indicated that PRDX1 interacts with the c-Myc oncogene and can inhibit its transcriptional activity [44] and high expression of PRDX1 appears to be associated with less aggressive breast cancers [45]. Therefore, upregulation of PRDX1 in this study may result from the defense of host cells responses to the ALV-J infection.

### 4.3. Cytoskeleton Proteins and ALV-J Infection

Cytoskeleton proteins are involved in the maintenance of cell morphology, regulation of protein synthesis, endocytosis, cell movement, and cell-to-cell attachment [46, 47]. As determined through iTRAQ LC-MS/MS analysis, some cytoskeleton proteins were identified to be significantly changed in DF-1 cells after infection with ALV-J. Isoform 2 of the F-actin-capping protein subunit beta isoforms 1 and 2 (CAPZB) can regulate the growth of actin filaments, and actin filaments play a vital role in the maintenance of cell morphology [48]. Furthermore, actin-related protein 3 (ACTR3) and actin-related protein 5 (ACTR5) were also found to be changed. The low expression of these proteins revealed that the cytoskeletal proteins were disrupted during infection with ALV-J. In addition, the differential expression of these proteins may be due to the interaction between the virus and host cellular proteins after infection with ALV-J.

It has been reported that keratins have become the standard detection marker for tumor cells and were also the most common marker to identify tumor cells [49]. Previous studies showed that before tumor cells got the ability to migrate and invade the host, they need to undergo epithelial-mesenchymal transition, during which the cytoskeletons are rearranged and epithelial markers, such as keratins, claudins, and E-cadherin, are observed to be downregulated [49–52]. Immunohistochemical analysis showed that low expression of keratin was associated with a higher tumor grade in breast cancer [52]. Previous study indicated that acetoacetyl-CoA synthetase (AACS) was found in tumor tissues and plays important roles in metabolic processes of tumors [53]. Whether or not the downregulated keratin and AACS in this study were associated with tumorigenesis induced by ALV-J infection needs to be further investigated.

## 5. Conclusions

In summary, our study was the first to use iTRAQ LC-MS/MS to detect cellular responses to ALV-J infection in DF-1 cells. A total of 75 significantly changed proteins were identified. These differently expressed proteins may provide useful information for elucidating the molecular mechanism underlying the interaction between ALV-J and DF-1 cells and will also facilitate our understanding of the pathogenesis of ALV-J infection.

## Supplementary Material

List of differentially expressed proteins identified by iTRAQ analysis of DF-1 cells infected with ALV-J. Fold change=infected/control. Fold change >1 indicates up regulation, and fold change <1 indicates down regulation.

## Figures and Tables

**Table 1:** 

Time	0	24	30	31	38
B%	3	16	30	90	90

**Table 2:** Primer sequences for real-time PCR.

Gene	Sequence	Size
BLOC1S5	F-TATATGAGCGGGGCAGGCCCT	150 bp
	R-TTCCCCGACATCCTTGAT	

Keratin	F-ATGTCCCGCTCCGTCAGCTTC	150 bp
	R-AGAGCCCAGGTTGTAGAGGCT	

HMG14	F-ATGCCGAAGAGAAAGGTG	140 bp
	R-TCAGATTTATCCTTAGCCGCC	

AACS	F-ATGTCCCGCGAGCCCGAGATT	150 bp
	R-CACTGACCACTGGTATAAGTC	

**Table 3:** List of significant differentially expressed proteins identified by iTRAQ analysis of DF-1 cells infected with ALV-J.

Accession number	Protein name	Protein score	Fold change in expression	Protein MW	Protein PI
Cluster 1: tendency for upregulation (33)					
O73612	Ephrin-B1 GN=EFNB1	34.98	1.667	36.8	8.87
F1P187	Gephyrin (fragment) GN=GPHN	0.00	1.560	77.4	5.38
P12274	Nonhistone chromosomal protein HMG-14B GN=HMG14	0.00	1.473	11.2	9.63
E1BTX9	Serine/threonine-protein phosphatase	39.24	1.429	73.4	8.34
P08286	Histone H1.10	476.50	1.315	22.0	11.18
E1C281	PHD finger protein 6 GN=PHF6	0.00	1.281	41.0	8.62
Q5ZJ02	DBIRD complex subunit ZNF326 GN=ZNF326	45.04	1.280	63.5	5.78
Q5ZIK4	Protein yippee-like GN=YPEL5	0.00	1.276	13.8	7.31
Q5F3J5	Proteasome activator complex subunit 3 GN=PSME3	143.05	1.274	29.5	6.19
Q5F3Z5	DnaJ homolog subfamily B member 6 GN=DNAJB6	0.00	1.263	36.7	8.84
F1NB51	Zinc finger E-box-binding homeobox 1 GN=ZEB1	41.75	1.258	123.1	5.02
F1NLA7	Zinc finger CCCH domain-containing protein 11A GN=ZC3H11A	43.83	1.254	79.0	8.16
F1P5W3	Ephrin-B1 (Fragment) GN=EFNB1	34.98	1.249	32.7	8.46
F1NXG2	WW domain-binding protein 4 (Fragment) GN=WBP4	0.00	1.235	45.2	5.73
P08267	Ferritin heavy chain GN=FTH	65.41	1.226	21.1	6.21
Q6K1L7	Probable RNA-binding protein EIF1AD GN=eif1ad	0.00	1.210	21.2	4.79
F1NEY0	Syndecan (Fragment) GN=CPQ	45.65	1.208	19.9	4.70
F1NMD7	Pre-mRNA-splicing factor RBM22 GN=RBM22	21.07	1.205	46.7	8.54
O93481	Chromobox protein (CHCB2) GN=CBX3	0.00	1.190	19.8	5.12
F1NFJ0	DNA replication licensing factor MCM3 GN=MCM3	46.00	1.188	91.3	5.74
E1C9E9	DCN1-like protein GN=DCUN1D5	0.00	1.183	27.2	5.77
F1NAQ1	Vascular endothelial growth factor A GN=VEGFA	0.00	1.179	25.1	9.10
F1NLU6	Enhancer of mRNA-decapping protein 3 GN=EDC3	49.54	1.175	56.0	7.17
P16527	Myristoylated alanine-rich C-kinase substrate GN=MARCKS	30.67	1.174	27.7	4.44
Q5ZMC9	Nuclear distribution protein nudE homolog 1 GN=NDE1	0.00	1.173	39.5	5.11
Q5ZII6	Protein kish-A GN=TMEM167A	27.01	1.173	8.0	8.92
Q5ZIL9	KIF1-binding protein homolog GN=kbp	0.00	1.167	69.0	5.21
F1NFP5	Arginine-tRNA ligase, cytoplasmic GN=RARS	184.95	1.158	75.4	6.98
E1C4V1	ATP synthase-coupling factor 6, mitochondrial GN=ATP5J	111.05	1.157	12.5	9.33
Q90595	Transcription factor MafF GN=MAFF	37.09	1.155	16.6	9.74
R4GJF8	TAR DNA-binding protein 43 GN=TARDBP	131.97	1.155	42.2	6.19
Q6B7Z6	Polymyositis/scleroderma autoantigen 1 GN=EXOSC9	0.00	1.151	49.3	5.54
E1C7X8	S-adenosylmethionine synthase GN=LOC427292	14.44	1.150	43.2	6.62

Cluster 2: Tendency to down-regulation (42)					
R4GKA6	Collagen alpha-2(VI) chain GN=COL6A2	181.18	0.850	102.4	5.48
E1BXS2	Guanine nucleotide-binding protein G(i) subunit alpha-1 GN=GNAI1	147.69	0.850	40.4	5.97
Q90927	Nuclear factor 1 GN=cNFI-A4	0.00	0.850	54.6	8.31
E1BUI0	tRNA pseudouridine synthase (Fragment) GN=PUSL1	55.04	0.849	33.7	9.64
Q90617-3	Isoform LAMP-2C of Lysosome-associated membrane glycoprotein 2 GN=LAMP2	149.29	0.849	46.4	6.43
Q90733	COUP transcription factor 2 GN=NR2F2	0.00	0.848	45.4	8.28
P12957-2	Isoform Brain l-cad of Caldesmon GN=CALD1	0.00	0.847	58.8	8.44
F1N9D8	Cathepsin B GN=CTSB	133.09	0.847	37.6	5.86
A4GTP0	Galectin	66.44	0.846	25.7	8.27
Q08392	Glutathione S-transferase	108.74	0.846	25.3	8.88
F1N965	Frizzled-7 GN=FZD7	0.00	0.842	62.7	7.99
E1C3U7	Lysyl oxidase homolog 2 GN=LOXL2	0.00	0.842	86.9	6.49
F1NGX1	Integrin alpha-V GN=ITGAV	172.35	0.841	114.3	5.58
E1BRJ4	DNA-directed RNA polymerase GN=POLR3B	0.00	0.840	127.4	8.54
Q8AXV1	Endophilin-A1 GN=SH3GL2	65.55	0.839	39.9	5.47
F1NMF6	Procollagen-lysine,2-oxoglutarate 5-dioxygenase 1 GN=PLOD1	198.96	0.837	84.3	6.74
F1P2F0	Collagen alpha-3(VI) chain GN=COL6A3	642.87	0.836	339.4	6.68
R4GFM0	FERM, RhoGEF and pleckstrin domain-containing protein 1 GN=FARP1	0.00	0.835	119.5	8.15
F1N8G4	Diphthamide biosynthesis protein 2 GN=DPH2	0.00	0.834	52.1	5.54
H9L0H3	Alpha-actinin-4 (Fragment) GN=ACTN4	515.20	0.834	71.6	6.09
Q5F4B1	Phosphoglycolate phosphatase GN=PGP	39.98	0.833	33.0	5.73
P56673	Pituitary homeobox 1 GN=PITX1	20.32	0.833	34.5	9.11
Q90611	72 kDa type IV collagenase GN=MMP2	267.53	0.829	74.9	5.49
F1NME2	Integrin beta GN=ITGB5	51.04	0.828	88.4	6.71
F1NBZ7	Serine/threonine-protein phosphatase GN=PPP3CA	0.00	0.827	60.6	5.83
P51890	Lumican GN=LUM	44.33	0.826	38.6	6.52
B3TZC1	PNPLA7 GN=PNPLA7	0.00	0.821	147.6	7.17
Q5ZLG0	Acetoacetyl-CoA synthetase GN=AACS	189.47	0.818	74.3	6.49
F1NLD4	Inhibitor of nuclear factor kappa-B kinase subunit alpha GN=CHUK	0.00	0.817	86.1	6.32
F1NFE0	Collagen alpha-1(VI) chain GN=COL6A1	110.52	0.812	107.9	5.90
F1N9N4	Stathmin-3 GN=NPC2	34.96	0.808	16.2	6.51
F1NPX5	SH3 domain-binding glutamic acid-rich-like protein (Fragment) GN=SH3BGRL	106.54	0.802	12.9	4.88
P01038	Cystatin	39.09	0.799	15.3	7.69
Q8QG94	Suppressor of fused GN=SUFU	46.78	0.786	53.7	5.33
Q90Y35-2	Isoform 2 of Zinc finger protein 622 GN=ZNF622	0.00	0.783	42.6	6.65
F1NMZ3	Hemoglobin subunit epsilon GN=HBE	37.75	0.761	16.6	8.91
Q5ZK77	Biogenesis of lysosome-related organelles complex 1 subunit 5 GN=BLOC1S5	34.23	0.759	22.6	7.06
F1P0D2	Glutamine synthetase (Fragment) GN=LOC417253	0.00	0.758	44.9	7.02
F1NJT4	Fibronectin GN=FN1	1373.06	0.752	259.0	6.11
Q155F6	Tumor necrosis factor-inducible protein 6 GN=TNFIP6	44.59	0.747	30.7	6.02
F1NJT3	Fibronectin GN=FN1	1373.06	0.727	273.1	5.64
O93532	Keratin, type II cytoskeletal cochlear	95.26	0.693	53.8	6.10

Fold change = infected/control. Fold change >1 indicates upregulation, and fold change <1 indicates downregulation.
