# Supplementary material for: Quantitative iTRAQ LC-MS/MS Proteomics Reveals the Proteome Profiles of DF-1 Cells after Infection with Subgroup J Avian Leukosis Virus
Source: Biomed Res Int. 2015 Jan 8;2015:395307. doi: 10.1155/2015/395307 (PMC4302370; doi:10.1155/2015/395307)
Supplement: Supplementary file 1 — List of differentially expressed proteins identified by iTRAQ analysis of DF-1 cells infected with ALV-J. Fold change=infected/control. Fold change >1 indicates up regulation, and fold change <1 indicates down regulation. [file 395307.f1.pdf]

List of differentially expressed proteins identified by iTRAQ analysis of DF-1 cells infected with ALV-J.

Accession no.	Protein name	Protein score	Fold change in expression	Protein MW	Protein PI
O73612	Ephrin-B1 OS=Gallus gallus GN=EFNB1 PE=2 SV=1 - [EFNB1_CHICK]	34.98	1.667	36.8	8.87
F1P187	Gephyrin (Fragment) OS=Gallus gallus GN=GPHN PE=4 SV=2 - [F1P187_CHICK]	0.00	1.560	77.4	5.38
P12274	Non-histone chromosomal protein HMG-14B OS=Gallus gallus GN=HMG14 PE=3 SV=2 - [HMGN1_CHICK]	0.00	1.473	11.2	9.63
E1BTX9	Serine/threonine-protein phosphatase OS=Gallus gallus PE=3 SV=2 - [E1BTX9_CHICK]	39.24	1.429	73.4	8.34
P08286	Histone H1.10 OS=Gallus gallus PE=1 SV=3 - [H110_CHICK]	476.50	1.315	22.0	11.18
E1C281	PHD finger protein 6 OS=Gallus gallus GN=PHF6 PE=4 SV=1 - [E1C281_CHICK]	0.00	1.281	41.0	8.62
Q5ZJ02	DBIRD complex subunit ZNF326 OS=Gallus gallus GN=ZNF326 PE=2 SV=3 - [ZN326_CHICK]	45.04	1.280	63.5	5.78
Q5ZIK4	Protein yippee-like OS=Gallus gallus GN=YPEL5 PE=2 SV=1 - [Q5ZIK4_CHICK]	0.00	1.276	13.8	7.31
Q5F3J5	Proteasome activator complex subunit 3 OS=Gallus gallus GN=PSME3 PE=1 SV=1 - [PSME3_CHICK]	143.05	1.274	29.5	6.19
Q5F3Z5	DnaJ homolog subfamily B member 6 OS=Gallus gallus GN=DNAJB6 PE=2 SV=1 - [DNJB6_CHICK]	0.00	1.263	36.7	8.84

F1NB51	Zinc finger E-box-binding homeobox 1 OS=Gallus gallus GN=ZEB1 PE=3 SV=1 - [F1NB51_CHICK]	41.75	1.258	123.1	5.02
F1NLA7	Zinc finger CCCH domain-containing protein 11A OS=Gallus gallus GN=ZC3H11A PE=4 SV=1 - [F1NLA7_CHICK]	43.83	1.254	79.0	8.16
F1P5W3	Ephrin-B1 (Fragment) OS=Gallus gallus GN=EFNB1 PE=3 SV=2 - [F1P5W3_CHICK]	34.98	1.249	32.7	8.46
F1NXG2	WW domain-binding protein 4 (Fragment) OS=Gallus gallus GN=WBP4 PE=4 SV=2 - [F1NXG2_CHICK]	0.00	1.235	45.2	5.73
P08267	Ferritin heavy chain OS=Gallus gallus GN=FTH PE=2 SV=2 - [FRIH_CHICK]	65.41	1.226	21.1	6.21
Q6K1L7	Probable RNA-binding protein EIF1AD OS=Gallus gallus GN=eif1ad PE=2 SV=1 - [EIF1A_CHICK]	0.00	1.210	21.2	4.79
F1NEY0	Syndecan (Fragment) OS=Gallus gallus GN=CPQ PE=3 SV=2 - [F1NEY0_CHICK]	45.65	1.208	19.9	4.70
F1NMD7	Pre-mRNA-splicing factor RBM22 OS=Gallus gallus GN=RBM22 PE=4 SV=1 - [F1NMD7_CHICK]	21.07	1.205	46.7	8.54
O93481	Chromobox protein (CHCB2) OS=Gallus gallus GN=CBX3 PE=2 SV=1 - [O93481_CHICK]	0.00	1.190	19.8	5.12
F1NFJ0	DNA replication licensing factor MCM3 OS=Gallus gallus GN=MCM3 PE=3 SV=1 - [F1NFJ0_CHICK]	46.00	1.188	91.3	5.74
E1C9E9	DCN1-like protein OS=Gallus gallus GN=DCUN1D5 PE=4 SV=2 - [E1C9E9_CHICK]	0.00	1.183	27.2	5.77
F1NAQ1	Vascular endothelial growth factor A OS=Gallus gallus GN=VEGFA PE=3 SV=1 - [F1NAQ1_CHICK]	0.00	1.179	25.1	9.10
F1NLU6	Enhancer of mRNA-decapping protein 3 OS=Gallus gallus GN=EDC3 PE=4 SV=1 - [F1NLU6_CHICK]	49.54	1.175	56.0	7.17
P16527	Myristoylated alanine-rich C-kinase substrate OS=Gallus gallus GN=MARCKS PE=1 SV=2 -	30.67	1.174	27.7	4.44

	[MARCS_CHICK]				
Q5ZMC9	Nuclear distribution protein nudE homolog 1 OS=Gallus gallus GN=NDE1 PE=2 SV=1 - [NDE1_CHICK]	0.00	1.173	39.5	5.11
Q5ZII6	Protein kish-A OS=Gallus gallus GN=TMEM167A PE=3 SV=1 - [KISHA_CHICK]	27.01	1.173	8.0	8.92
Q5ZIL9	KIF1-binding protein homolog OS=Gallus gallus GN=kbp PE=2 SV=1 - [KBP_CHICK]	0.00	1.167	69.0	5.21
F1NFP5	Arginine--tRNA ligase, cytoplasmic OS=Gallus gallus GN=RARS PE=3 SV=2 - [F1NFP5_CHICK]	184.95	1.158	75.4	6.98
E1C4V1	ATP synthase-coupling factor 6, mitochondrial OS=Gallus gallus GN=ATP5J PE=3 SV=2 - [E1C4V1_CHICK]	111.05	1.157	12.5	9.33
Q90595	Transcription factor MafF OS=Gallus gallus GN=MAFF PE=2 SV=1 - [MAFF_CHICK]	37.09	1.155	16.6	9.74
R4GJF8	TAR DNA-binding protein 43 OS=Gallus gallus GN=TARDBP PE=4 SV=1 - [R4GJF8_CHICK]	131.97	1.155	42.2	6.19
Q6B7Z6	Polymyositis/scleroderma autoantigen 1 OS=Gallus gallus GN=EXOSC9 PE=2 SV=1 - [Q6B7Z6_CHICK]	0.00	1.151	49.3	5.54
E1C7X8	S-adenosylmethionine synthase OS=Gallus gallus GN=LOC427292 PE=3 SV=1 - [E1C7X8_CHICK]	14.44	1.150	43.2	6.62
Q5ZLD4	Transmembrane protein 11, mitochondrial OS=Gallus gallus GN=TMEM11 PE=2 SV=1 - [TMM11_CHICK]	46.41	1.149	21.7	7.36
R4GHT3	Uridine-cytidine kinase (Fragment) OS=Gallus gallus GN=LOC776992 PE=3 SV=1 - [R4GHT3_CHICK]	0.00	1.148	49.7	7.03
Q9I8G9	Histone-binding protein RBBP7 OS=Gallus gallus GN=RBBP7 PE=1 SV=1 - [RBBP7_CHICK]	111.20	1.146	47.7	4.94
F1NT19	Superoxide dismutase OS=Gallus gallus GN=SOD2 PE=3 SV=1 - [F1NT19_CHICK]	45.73	1.146	24.8	8.41

F1P578	Mitochondrial Rho GTPase OS=Gallus gallus GN=RHOT2 PE=3 SV=2 - [F1P578_CHICK]	79.27	1.139	69.1	5.24
F1NA55	Eukaryotic translation initiation factor 2A OS=Gallus gallus GN=EIF2A PE=4 SV=2 - [F1NA55_CHICK]	66.41	1.138	62.7	8.79
Q5ZKR5	Nuclear cap-binding protein subunit 2 OS=Gallus gallus GN=NCBP2 PE=2 SV=1 - [NCBP2_CHICK]	0.00	1.137	19.3	9.00
Q5ZIR9	E3 ubiquitin-protein ligase RNF185 OS=Gallus gallus GN=RNF185 PE=2 SV=1 - [RN185_CHICK]	23.79	1.134	20.8	7.62
Q5ZHN4	Protein XRP2 OS=Gallus gallus GN=RP2 PE=2 SV=3 - [XRP2_CHICK]	19.86	1.132	39.7	5.29
P13127	F-actin-capping protein subunit alpha-1 OS=Gallus gallus GN=CAPZA1 PE=1 SV=1 - [CAZA1_CHICK]	44.43	1.132	32.9	5.69
P18944	Cytochrome c oxidase subunit 2 OS=Gallus gallus GN=MT-CO2 PE=3 SV=1 - [COX2_CHICK]	0.00	1.132	25.6	4.72
F1NCR4	Ubiquitin carboxyl-terminal hydrolase OS=Gallus gallus PE=3 SV=2 - [F1NCR4_CHICK]	69.75	1.131	130.2	5.21
Q800L1	Surfeit locus protein 1 OS=Gallus gallus GN=SURF1 PE=3 SV=1 - [SURF1_CHICK]	47.36	1.128	35.2	9.74
Q5ZKJ0	Cleft lip and palate transmembrane protein 1-like protein OS=Gallus gallus GN=CLPTM1L PE=2 SV=1 - [CLP1L_CHICK]	0.00	1.128	61.6	8.97
Q90593	78 kDa glucose-regulated protein OS=Gallus gallus GN=HSPA5 PE=1 SV=1 - [GRP78_CHICK]	3036.20	1.126	72.0	5.22
F1NNS3	Protein HIRA (Fragment) OS=Gallus gallus GN=HIRA PE=4 SV=1 - [F1NNS3_CHICK]	65.23	1.124	110.6	7.88
Q5ZM57	Very-long-chain (3R)-3-hydroxyacyl-[acyl-carrier protein] dehydratase OS=Gallus gallus GN=PTPLAD1 PE=2 SV=1 - [HACD_CHICK]	17.88	1.122	42.8	9.06

P56517	Histone deacetylase 1 OS=Gallus gallus GN=HDAC1 PE=2 SV=1 - [HDAC1_CHICK]	51.95	1.121	54.9	5.49
Q76LT9	Transmembrane protein 258 OS=Gallus gallus GN=TMEM258 PE=3 SV=1 - [TM258_CHICK]	0.00	1.121	9.1	5.83
F1NU71	Mitochondrial import inner membrane translocase subunit TIM44 OS=Gallus gallus GN=TIMM44 PE=3 SV=2 - [F1NU71_CHICK]	160.28	1.120	51.2	8.12
Q98TQ8	Connective tissue growth factor (Precursor) OS=Gallus gallus GN=CTGF PE=2 SV=1 - [Q98TQ8_CHICK]	0.00	1.120	37.5	7.83
F1NVA4	Nucleophosmin (Fragment) OS=Gallus gallus GN=NPM1 PE=4 SV=2 - [F1NVA4_CHICK]	268.66	1.119	30.3	4.84
R4GGG5	Microfibrillar-associated protein 1 OS=Gallus gallus GN=MFAP1 PE=4 SV=1 - [R4GGG5_CHICK]	37.12	1.118	51.9	4.97
Q5ZJM2	Tryptophan rich basic protein OS=Gallus gallus GN=WRB PE=2 SV=1 - [Q5ZJM2_CHICK]	0.00	1.116	14.4	9.83
Q90640	Chromosome-associated kinesin KIF4 OS=Gallus gallus GN=KIF4 PE=2 SV=1 - [KIF4_CHICK]	41.66	1.116	138.8	6.52
P04268-4	Isoform 4 of Tropomyosin alpha-1 chain OS=Gallus gallus GN=TPM1 - [TPM1_CHICK]	328.58	1.115	32.9	4.78
Q5ZMC0	Endothelial differentiation-related factor 1 homolog OS=Gallus gallus GN=EDF1 PE=2 SV=1 - [EDF1_CHICK]	45.41	1.114	16.4	9.88
P02263	Histone H2A-IV OS=Gallus gallus PE=1 SV=2 - [H2A4_CHICK]	1212.94	1.113	13.9	10.90
F1P5R3	Chromatin assembly factor 1 subunit A (Fragment) OS=Gallus gallus GN=CHAF1A PE=4 SV=1 - [F1P5R3_CHICK]	0.00	1.111	105.0	5.31

Q5ZL36	Protrudin OS=Gallus gallus GN=ZFYVE27 PE=2 SV=1 - [ZFY27_CHICK]	35.29	1.111	44.9	5.45
R4GHV2	DCN1-like protein OS=Gallus gallus GN=DCUN1D2 PE=4 SV=1 - [R4GHV2_CHICK]	0.00	1.111	28.5	5.22
Q5ZLC4	Oxysterol-binding protein OS=Gallus gallus GN=OSBPL2 PE=2 SV=1 - [Q5ZLC4_CHICK]	0.00	1.110	55.3	5.94
P62167	Neuronal calcium sensor 1 OS=Gallus gallus GN=NCS1 PE=1 SV=2 - [NCS1_CHICK]	0.00	1.110	21.9	4.83
Q5ZHM2	DNA-binding protein Ikaros OS=Gallus gallus GN=IK PE=2 SV=1 - [Q5ZHM2_CHICK]	73.56	1.110	64.9	6.64
Q2VB19-2	Isoform 2 of Pumilio homolog 1 OS=Gallus gallus GN=PUM1 - [PUM1_CHICK]	40.66	1.109	116.3	6.84
Q5ZLY8	cAMP-regulated phosphoprotein 19 OS=Gallus gallus GN=ARPP19 PE=3 SV=3 - [ARP19_CHICK]	0.00	1.109	12.3	9.09
Q90VY6	Survivin gamma OS=Gallus gallus GN=BIRC5 PE=4 SV=1 - [Q90VY6_CHICK]	0.00	1.109	6.7	9.20
Q5ZME8	WD40 repeat-containing protein SMU1 OS=Gallus gallus GN=SMU1 PE=2 SV=1 - [SMU1_CHICK]	30.87	1.109	57.5	7.18
F1NI57	Pre-mRNA-splicing factor CWC22 homolog OS=Gallus gallus GN=CWC22 PE=4 SV=2 - [F1NI57_CHICK]	0.00	1.109	108.9	7.58
F1NFC0	Nuclease-sensitive element-binding protein 1 (Fragment) OS=Gallus gallus GN=YBX1 PE=4 SV=2 - [F1NFC0_CHICK]	168.61	1.108	31.1	9.98
P30352	Serine/arginine-rich splicing factor 2 OS=Gallus gallus GN=SRSF2 PE=2 SV=1 - [SRSF2_CHICK]	19.02	1.108	25.5	11.85
Q5ZL84	Sigma non-opioid intracellular receptor 1 OS=Gallus gallus GN=SIGMAR1 PE=2 SV=1 - [SGMR1_CHICK]	27.57	1.107	24.6	6.33
F1NLF2	KH domain-containing, RNA-binding, signal transduction-associated protein 1 OS=Gallus gallus	70.38	1.107	46.5	8.81

	GN=KHDRBS1 PE=4 SV=2 - [F1NLF2_CHICK]				
Q90596	Transcription factor MafK OS=Gallus gallus GN=MAFK PE=2 SV=1 - [MAFK_CHICK]	49.33	1.107	17.5	9.89
O13270	TATA-box-binding protein OS=Gallus gallus GN=TBP PE=2 SV=1 - [TBP_CHICK]	66.94	1.107	33.1	9.79
P18660	60S acidic ribosomal protein P1 OS=Gallus gallus GN=RPLP1 PE=3 SV=1 - [RLA1_CHICK]	76.70	1.107	11.5	4.21
Q9PUJ4	Ephrin-B2 (Precursor) OS=Gallus gallus GN=EFNB2 PE=2 SV=1 - [Q9PUJ4_CHICK]	0.00	1.106	36.7	8.84
Q98TF7	60S ribosomal protein L35 OS=Gallus gallus GN=RPL35 PE=2 SV=3 - [RL35_CHICK]	82.74	1.105	14.5	11.05
F1N874	Myocyte-specific enhancer factor 2A OS=Gallus gallus GN=MEF2A PE=3 SV=2 - [F1N874_CHICK]	0.00	1.104	52.7	8.69
F1NTH4	Actin filament-associated protein 1 OS=Gallus gallus GN=AFAP1 PE=4 SV=1 - [F1NTH4_CHICK]	42.00	1.104	81.1	8.63
Q9I882	Protein kinase C inhibitor OS=Gallus gallus GN=chPKCI PE=2 SV=1 - [Q9I882_CHICK]	57.68	1.104	13.8	6.79
P13216	Adrenodoxin, mitochondrial (Fragment) OS=Gallus gallus GN=FDX1 PE=1 SV=1 - [ADX_CHICK]	0.00	1.103	15.5	4.88
E1C6F0	U1 small nuclear ribonucleoprotein C OS=Gallus gallus GN=SNRPC PE=3 SV=1 - [RU1C_CHICK]	89.22	1.102	17.4	9.67
P67881	Cytochrome c OS=Gallus gallus GN=CYC PE=1 SV=2 - [CYC_CHICK]	232.97	1.102	11.7	9.50
R4GHZ1	Activating transcription factor 7-interacting protein 1 OS=Gallus gallus GN=ATF7IP PE=4 SV=1 - [R4GHZ1_CHICK]	0.00	1.102	102.9	5.58
Q5ZJA2	Anamorsin OS=Gallus gallus GN=CIAPIN1 PE=2 SV=1 - [CPIN1_CHICK]	77.01	1.101	32.6	5.12

P62801	Histone H4 OS=Gallus gallus GN=H4-I PE=1 SV=2 - [H4_CHICK]	1168.26	1.101	11.4	11.36
E1C697	Glutathione peroxidase OS=Gallus gallus GN=GPX7 PE=3 SV=1 - [E1C697_CHICK]	66.32	1.100	23.5	7.96
F1NWG6	Protein VAC14 homolog OS=Gallus gallus GN=VAC14 PE=4 SV=1 - [F1NWG6_CHICK]	51.78	1.100	87.8	6.30
E1C5S5	Protein kinase C delta type OS=Gallus gallus GN=PRKCD PE=3 SV=2 - [E1C5S5_CHICK]	0.00	1.099	78.4	8.12
F1P3F9	Glutamate dehydrogenase OS=Gallus gallus GN=GLUD1 PE=3 SV=2 - [F1P3F9_CHICK]	405.85	1.099	47.6	8.18
P49416	Syndecan-4 OS=Gallus gallus GN=SDC4 PE=1 SV=1 - [SDC4_CHICK]	34.52	1.099	21.5	4.45
P08636	40S ribosomal protein S17 OS=Gallus gallus GN=RPS17 PE=2 SV=3 - [RS17_CHICK]	47.58	1.099	15.5	9.85
F1NP38	TBC1 domain family member 23 OS=Gallus gallus GN=TBC1D23 PE=4 SV=1 - [F1NP38_CHICK]	0.00	1.098	76.1	5.36
Q5ZMW3	Apoptosis inhibitor 5 OS=Gallus gallus GN=API5 PE=2 SV=1 - [API5_CHICK]	247.27	1.098	58.6	7.39
F1NI14	Taxilin gamma OS=Gallus gallus GN=TXLNG PE=4 SV=2 - [F1NI14_CHICK]	37.79	1.098	56.5	5.96
Q9PT88	RNA polymerase common subunit RPB6 OS=Gallus gallus GN=RPB6 PE=2 SV=1 - [Q9PT88_CHICK]	31.69	1.097	14.5	4.22
F1P0Q8	H/ACA ribonucleoprotein complex subunit 4 OS=Gallus gallus GN=DKC1 PE=4 SV=2 - [F1P0Q8_CHICK]	69.01	1.097	58.1	9.28
F1NHW3	Enhancer of rudimentary homolog OS=Gallus gallus GN=ERH PE=3 SV=1 - [F1NHW3_CHICK]	35.65	1.096	12.3	5.92
P08284	Histone H1.01 OS=Gallus gallus PE=1 SV=2 - [H101_CHICK]	487.67	1.095	22.0	11.17

Q5ZI57	Trafficking protein particle complex subunit 3 OS=Gallus gallus GN=TRAPPC3 PE=2 SV=1 - [TPPC3_CHICK]	37.93	1.094	20.3	4.96
P17926	Nuclear factor 1 C-type OS=Gallus gallus GN=NFIC PE=1 SV=1 - [NFIC_CHICK]	0.00	1.094	49.2	8.12
E1C8R3	Carbohydrate sulfotransferase 3 OS=Gallus gallus GN=CHST3 PE=4 SV=2 - [E1C8R3_CHICK]	0.00	1.094	52.2	8.95
Q5XNV3	Cytoplasmic activation-proliferation-associated protein 1 OS=Gallus gallus GN=CAPRIN1 PE=2 SV=1 - [Q5XNV3_CHICK]	146.74	1.093	77.9	5.16
F1NMM9	Uridine-cytidine kinase OS=Gallus gallus GN=UCK2 PE=3 SV=2 - [F1NMM9_CHICK]	16.50	1.093	29.4	5.97
Q5F334	Leucine-rich repeat-containing protein 59 OS=Gallus gallus GN=LRRC59 PE=2 SV=1 - [LRC59_CHICK]	148.66	1.093	38.6	9.23
R4GH14	Charged multivesicular body protein 6 (Fragment) OS=Gallus gallus GN=CHMP6 PE=4 SV=1 - [R4GH14_CHICK]	0.00	1.092	12.7	4.09
E1BUW6	Microtubule-associated protein OS=Gallus gallus PE=4 SV=2 - [E1BUW6_CHICK]	264.37	1.092	86.1	9.04
F1NZW0	Transcription initiation factor TFIID subunit 3 OS=Gallus gallus GN=TAF3 PE=4 SV=2 - [F1NZW0_CHICK]	26.71	1.091	103.1	8.95
F1NYC4	Clathrin heavy chain (Fragment) OS=Gallus gallus GN=CLTCL1 PE=3 SV=1 - [F1NYC4_CHICK]	361.39	1.090	191.8	5.85
P84175	40S ribosomal protein S12 OS=Gallus gallus GN=RPS12 PE=1 SV=2 - [RS12_CHICK]	66.86	1.089	14.5	7.21
Q5F450	PAB-dependent poly(A)-specific ribonuclease subunit 2 OS=Gallus gallus GN=PAN2 PE=2 SV=1 - [PAN2_CHICK]	47.48	1.089	134.8	5.68

F1NGH3	Nuclear factor NF-kappa-B p100 subunit (Fragment) OS=Gallus gallus GN=NFKB2 PE=4 SV=2 - [F1NGH3_CHICK]	0.00	1.089	98.0	6.42
Q5F470	Ras-related protein Rab-8A OS=Gallus gallus GN=RAB8A PE=2 SV=1 - [RAB8A_CHICK]	93.13	1.089	23.5	9.09
Q5F471	Serine/threonine-protein phosphatase 6 regulatory subunit 3 OS=Gallus gallus GN=PPP6R3 PE=2 SV=1 - [PP6R3_CHICK]	38.49	1.089	98.2	4.58
F1NB93	Peroxisomal targeting signal 1 receptor (Fragment) OS=Gallus gallus GN=PEX5 PE=4 SV=2 - [F1NB93_CHICK]	0.00	1.089	66.5	4.68
P62764	Visinin-like protein 1 OS=Gallus gallus GN=VSNL1 PE=2 SV=2 - [VISL1_CHICK]	0.00	1.088	22.1	5.15
O42130	DNA topoisomerase 2-alpha OS=Gallus gallus GN=TOP2A PE=2 SV=2 - [TOP2A_CHICK]	101.55	1.088	174.9	8.81
F1NAZ3	Kinesin-like protein KIF2A OS=Gallus gallus GN=KIF2A PE=3 SV=2 - [F1NAZ3_CHICK]	19.08	1.086	79.9	6.68
F1NCR3	Cytosolic purine 5'-nucleotidase OS=Gallus gallus GN=NT5C2 PE=3 SV=1 - [F1NCR3_CHICK]	38.34	1.086	65.9	5.49
Q1KME6	Ras-related protein Rab-6A OS=Gallus gallus GN=RAB6A PE=2 SV=3 - [RAB6A_CHICK]	326.78	1.086	23.5	5.54
F1NGV1	Zinc finger CCCH domain-containing protein 15 (Fragment) OS=Gallus gallus GN=ZC3H15 PE=4 SV=2 - [F1NGV1_CHICK]	37.23	1.085	48.7	5.10
F2Z4M0	Histone-binding protein RBBP4 OS=Gallus gallus GN=RBBP4 PE=4 SV=1 - [F2Z4M0_CHICK]	120.37	1.084	47.6	4.89
P28337	Aminomethyltransferase, mitochondrial OS=Gallus gallus GN=AMT PE=2 SV=2 - [GCST_CHICK]	0.00	1.084	42.0	8.88

F1NUY9	Ufm1-specific protease 2 OS=Gallus gallus GN=UFSP2 PE=4 SV=1 - [F1NUY9_CHICK]	0.00	1.084	52.1	6.70
Q90631	Kinectin OS=Gallus gallus GN=KTN1 PE=1 SV=1 - [KTN1_CHICK]	879.49	1.084	155.9	5.90
Q5ZMR3	Protein-L-isoaspartate O-methyltransferase domain-containing protein 1 OS=Gallus gallus GN=PCMTD1 PE=2 SV=1 - [PCMD1_CHICK]	0.00	1.083	40.9	5.91
P84247	Histone H3.3 OS=Gallus gallus GN=H3-IX PE=1 SV=2 - [H33_CHICK]	39.79	1.083	15.3	11.27
Q5ZL33	Serine-threonine kinase receptor-associated protein OS=Gallus gallus GN=STRAP PE=2 SV=2 - [STRAP_CHICK]	249.06	1.083	38.1	5.11
F1P4P0	Ubiquitin-fold modifier 1 (Fragment) OS=Gallus gallus GN=UFM1 PE=4 SV=1 - [F1P4P0_CHICK]	34.95	1.083	9.0	9.60
A0M8T8	Caveolin OS=Gallus gallus GN=CAV1 PE=3 SV=1 - [A0M8T8_CHICK]	69.51	1.082	20.5	6.28
Q5ZKK2	Integrator complex subunit 9 OS=Gallus gallus GN=INTS9 PE=2 SV=1 - [INT9_CHICK]	0.00	1.082	73.7	6.37
Q5H7N8	Zinc finger CCCH domain-containing protein 15 OS=Gallus gallus GN=ZC3H15 PE=1 SV=1 - [ZC3HF_CHICK]	35.04	1.081	49.0	5.16
F1NU40	Nucleolin OS=Gallus gallus GN=NCL PE=4 SV=2 - [F1NU40_CHICK]	775.81	1.081	75.4	4.92
Q5ZJ65	Protein FAM76A OS=Gallus gallus GN=FAM76A PE=2 SV=1 - [FA76A_CHICK]	36.72	1.081	34.9	9.14
F6UZR6	Lamin-B receptor OS=Gallus gallus GN=LBR PE=4 SV=1 - [F6UZR6_CHICK]	56.08	1.080	73.3	9.07

E1C6Y7	pre-rRNA-processing protein FTSJ3 OS=Gallus gallus GN=FTSJ3 PE=3 SV=2 - [E1C6Y7_CHICK]	74.07	1.080	92.6	6.92
R4GJ94	Dynamin-like 120 kDa protein, mitochondrial OS=Gallus gallus GN=OPA1 PE=3 SV=1 - [R4GJ94_CHICK]	66.75	1.080	115.7	8.34
F2Z4M3	40S ribosomal protein S15 (Fragment) OS=Gallus gallus GN=RPS15 PE=3 SV=1 - [F2Z4M3_CHICK]	0.00	1.080	16.9	10.39
P09645	Tubulin alpha-8 chain (Fragment) OS=Gallus gallus PE=3 SV=1 - [TBA8_CHICK]	462.63	1.080	36.1	4.93
Q2MCJ7	Aquaporin-1 OS=Gallus gallus GN=AQP1 PE=2 SV=1 - [Q2MCJ7_CHICK]	0.00	1.079	28.5	6.90
Q8JGM4	Sulfhydryl oxidase 1 OS=Gallus gallus GN=QSOX1 PE=1 SV=1 - [QSOX1_CHICK]	36.43	1.078	83.0	7.40
Q5ZJS6	Polyadenylate-binding protein-interacting protein 2 OS=Gallus gallus GN=PAIP2 PE=2 SV=1 - [PAIP2_CHICK]	21.33	1.078	15.0	4.12
Q5ZHV6	GrpE protein homolog OS=Gallus gallus GN=GRPEL1 PE=2 SV=1 - [Q5ZHV6_CHICK]	145.11	1.078	24.8	7.49
Q5ZJF1	COP9 signalosome complex subunit 3 OS=Gallus gallus GN=COPS3 PE=2 SV=1 - [CSN3_CHICK]	0.00	1.078	47.9	6.79
F1NH21	Hsc70-interacting protein OS=Gallus gallus GN=ST13 PE=4 SV=1 - [F1NH21_CHICK]	59.23	1.078	40.2	5.14
Q5ZL00	ER membrane protein complex subunit 1 OS=Gallus gallus GN=EMC1 PE=2 SV=1 - [EMC1_CHICK]	111.61	1.078	109.8	7.52
Q5F3A1	Translocation protein SEC62 OS=Gallus gallus GN=SEC62 PE=2 SV=1 - [SEC62_CHICK]	40.32	1.078	45.9	7.08
F1NP51	Lamin-B2 OS=Gallus gallus GN=LMNB2 PE=3 SV=1 - [F1NP51_CHICK]	458.32	1.077	67.9	5.27
Q90879	Ubiquitin-conjugating enzyme E2 variant 1 OS=Gallus gallus GN=UBE2V1 PE=2 SV=2 - [UB2V1_CHICK]	83.22	1.077	16.3	7.94

F1NFV6	Fos-related antigen 2 OS=Gallus gallus GN=FOSL2 PE=4 SV=2 - [F1NFV6_CHICK]	34.46	1.077	33.1	8.97
Q9YH18-2	Isoform 2 of Protein quaking OS=Gallus gallus GN=QKI - [QKI_CHICK]	119.51	1.077	36.8	8.56
F1NYB3	Nucleolar and spindle-associated protein 1 OS=Gallus gallus GN=NUSAP1 PE=4 SV=1 - [F1NYB3_CHICK]	0.00	1.076	52.2	9.94
E1BYG9	Coiled-coil domain-containing protein 50 OS=Gallus gallus GN=CCDC50 PE=4 SV=2 - [E1BYG9_CHICK]	0.00	1.076	35.4	7.34
P02272	Histone H2A.V OS=Gallus gallus GN=H2AFV PE=1 SV=2 - [H2AV_CHICK]	417.72	1.075	13.5	10.58
F1NZ28	Anoctamin OS=Gallus gallus GN=ANO10 PE=3 SV=2 - [F1NZ28_CHICK]	70.84	1.075	70.9	7.33
F1N9Z7	Succinyl-CoA:3-ketoacid-coenzyme A transferase OS=Gallus gallus GN=OXCT1 PE=3 SV=2 - [F1N9Z7_CHICK]	484.87	1.075	56.1	7.91
P0C1H3	Histone H2B 1/2/3/4/6 OS=Gallus gallus GN=H2B-I PE=1 SV=2 - [H2B1_CHICK]	644.90	1.075	13.9	10.27
E1C8U1	Nucleoporin NUP53 OS=Gallus gallus GN=NUP35 PE=3 SV=2 - [E1C8U1_CHICK]	57.92	1.074	34.5	9.07
F1NID5	Ubiquitin-associated domain-containing protein 1 (Fragment) OS=Gallus gallus GN=UBAC1 PE=4 SV=2 - [F1NID5_CHICK]	0.00	1.073	45.4	5.02
E1BQL2	Serine/arginine repetitive matrix protein 1 OS=Gallus gallus GN=SRRM1 PE=4 SV=2 - [E1BQL2_CHICK]	44.16	1.072	100.8	11.82
Q90679	Thymocyte nuclear protein 1 OS=Gallus gallus GN=THYN1 PE=1 SV=1 - [THYN1_CHICK]	102.91	1.072	27.9	8.29
O42395	Cellular nucleic acid-binding protein OS=Gallus gallus GN=CNBP PE=2 SV=1 - [CNBP_CHICK]	37.77	1.072	19.0	7.83

F1P4U4	Ubiquitin carboxyl-terminal hydrolase (Fragment) OS=Gallus gallus GN=USP7 PE=3 SV=1 - [F1P4U4_CHICK]	94.04	1.071	125.4	5.71
F1NWB7	Endoplasmin OS=Gallus gallus GN=HSP90B1 PE=3 SV=1 - [F1NWB7_CHICK]	534.94	1.070	91.5	4.89
F1N988	Clustered mitochondria protein homolog OS=Gallus gallus GN=KIAA0664 PE=3 SV=2 - [F1N988_CHICK]	0.00	1.070	150.4	6.10
Q5ZLM0	Parafibromin OS=Gallus gallus GN=CDC73 PE=2 SV=1 - [CDC73_CHICK]	41.39	1.070	60.7	9.61
G1K2Z3	Paired mesoderm homeobox protein 1 OS=Gallus gallus GN=PRRX1 PE=3 SV=1 - [G1K2Z3_CHICK]	54.11	1.070	27.2	9.47
Q5F3D1	Zinc finger CCHC domain-containing protein 8 OS=Gallus gallus GN=ZCCHC8 PE=2 SV=1 - [ZCHC8_CHICK]	0.00	1.070	68.4	5.05
E1C8Q5	V-type proton ATPase 116 kDa subunit a isoform 1 OS=Gallus gallus GN=ATP6V0A1 PE=4 SV=2 - [E1C8Q5_CHICK]	77.80	1.070	95.3	6.74
Q5ZK33	LETM1 and EF-hand domain-containing protein 1, mitochondrial OS=Gallus gallus GN=LETM1 PE=2 SV=1 - [LETM1_CHICK]	155.92	1.070	85.8	6.71
R4GKR9	DNA replication complex GINS protein PSF2 OS=Gallus gallus GN=GINS2 PE=3 SV=1 - [R4GKR9_CHICK]	53.13	1.069	21.2	5.07
Q5ZL72	60 kDa heat shock protein, mitochondrial OS=Gallus gallus GN=HSPD1 PE=1 SV=1 - [CH60_CHICK]	1427.97	1.069	60.9	5.87
Q4JIM4	Presenilin-1 OS=Gallus gallus GN=PSEN1 PE=1 SV=1 - [PSN1_CHICK]	18.46	1.069	52.8	5.10
P63270	Actin, gamma-enteric smooth muscle OS=Gallus gallus GN=ACTG2 PE=1 SV=1 - [ACTH_CHICK]	1825.98	1.068	41.8	5.48

P07322	Beta-enolase OS=Gallus gallus GN=ENO3 PE=1 SV=3 - [ENOB_CHICK]	35.08	1.068	47.2	7.61
F1P4J0	Ribosomal protein S6 kinase OS=Gallus gallus GN=RPS6KB1 PE=3 SV=1 - [F1P4J0_CHICK]	0.00	1.068	56.1	6.44
P32882	Tubulin beta-2 chain OS=Gallus gallus PE=2 SV=1 - [TBB2_CHICK]	835.42	1.067	49.9	4.89
E1C0E5	Transmembrane protein 9B OS=Gallus gallus GN=TMEM9B PE=2 SV=2 - [E1C0E5_CHICK]	27.15	1.066	21.3	7.84
Q5F477	UPF0554 protein C2orf43 homolog OS=Gallus gallus GN=RCJMB04_2g19 PE=2 SV=2 - [CB043_CHICK]	0.00	1.066	36.9	7.33
Q90955	Prostaglandin E synthase 3 (Fragment) OS=Gallus gallus GN=PTGES3 PE=1 SV=1 - [TEBP_CHICK]	80.47	1.066	16.9	4.41
Q5ZLL0	Vacuolar ATPase assembly integral membrane protein VMA21 OS=Gallus gallus GN=VMA21 PE=3 SV=1 - [VMA21_CHICK]	0.00	1.066	11.6	7.24
F6RW83	Ribosomal protein L19 OS=Gallus gallus GN=RPL19 PE=3 SV=1 - [F6RW83_CHICK]	114.74	1.065	23.2	11.36
F1NHH9	D-beta-hydroxybutyrate dehydrogenase, mitochondrial OS=Gallus gallus GN=BDH1 PE=3 SV=1 - [F1NHH9_CHICK]	32.32	1.065	38.0	7.93
E1C1L6	Ectonucleoside triphosphate diphosphohydrolase 5 OS=Gallus gallus GN=ENTPD5 PE=3 SV=1 - [ENTP5_CHICK]	0.00	1.065	47.9	5.62
F1NDP5	Thiosulfate sulfurtransferase OS=Gallus gallus GN=TST PE=4 SV=2 - [F1NDP5_CHICK]	59.60	1.065	32.9	7.58
Q5ZIL4	Spermatid perinuclear RNA-binding protein OS=Gallus gallus GN=STRBP PE=2 SV=1 - [STRBP_CHICK]	0.00	1.064	73.3	8.73

P47826	60S acidic ribosomal protein P0 OS=Gallus gallus GN=RPLP0 PE=2 SV=1 - [RLA0_CHICK]	302.72	1.064	34.3	5.99
Q90ZK6	Activin receptor type-1 OS=Gallus gallus GN=ACVR1 PE=2 SV=1 - [ACVR1_CHICK]	84.41	1.064	56.2	7.11
R4GLP7	Citrate synthase OS=Gallus gallus GN=LOC100858903 PE=3 SV=1 - [R4GLP7_CHICK]	218.37	1.063	42.5	6.99
Q5ZHP3	UPF0468 protein C16orf80 homolog OS=Gallus gallus GN=RCJMB04_34o2 PE=2 SV=1 - [CP080_CHICK]	0.00	1.061	22.7	9.76
Q5ZML4	Cysteine and histidine-rich domain-containing protein 1 OS=Gallus gallus GN=CHORDC1 PE=2 SV=1 - [CHRD1_CHICK]	0.00	1.061	37.3	7.24
H9L2N7	Protein pelota homolog OS=Gallus gallus GN=PELO PE=4 SV=2 - [H9L2N7_CHICK]	0.00	1.061	43.5	6.09
F1NLF1	Hydroxysteroid dehydrogenase-like protein 1 OS=Gallus gallus GN=HSDL1 PE=3 SV=1 - [F1NLF1_CHICK]	36.62	1.061	37.6	9.14
F1NGU9	Cleavage and polyadenylation-specificity factor subunit 6 OS=Gallus gallus GN=CPSF6 PE=4 SV=1 - [F1NGU9_CHICK]	173.74	1.060	59.1	7.40
F1NAD0	LysM and putative peptidoglycan-binding domain-containing protein 3 OS=Gallus gallus GN=LYSMD3 PE=4 SV=1 - [F1NAD0_CHICK]	34.26	1.060	33.4	6.86
Q5ZJ40	REST corepressor 3 OS=Gallus gallus GN=RCOR3 PE=2 SV=1 - [RCOR3_CHICK]	47.74	1.059	42.4	8.65
Q5ZHV1	Ras-related protein Rab-33B OS=Gallus gallus GN=RAB33B PE=2 SV=1 - [RB33B_CHICK]	0.00	1.059	25.7	8.05
F1NKB6	6-phosphofructokinase OS=Gallus gallus GN=PFKL PE=3 SV=2 - [F1NKB6_CHICK]	44.25	1.059	84.0	7.43

F1NKG6	Protein CYR61 (Fragment) OS=Gallus gallus GN=CYR61 PE=4 SV=2 - [F1NKG6_CHICK]	0.00	1.058	33.9	8.09
Q5ZM44	Calcineurin B homologous protein 1 OS=Gallus gallus GN=CHP1 PE=1 SV=3 - [CHP1_CHICK]	71.56	1.058	22.4	5.10
Q9YGL6-2	Isoform 2 of Paralemmin-1 OS=Gallus gallus GN=PALM - [PALM_CHICK]	0.00	1.058	35.0	4.84
P84229	Histone H3.2 OS=Gallus gallus GN=H3-I PE=1 SV=2 - [H32_CHICK]	22.98	1.058	15.4	11.27
F1NF89	40S ribosomal protein S24 OS=Gallus gallus GN=RPS24 PE=3 SV=2 - [F1NF89_CHICK]	110.69	1.058	15.6	10.78
F1NYY5	Phosphoethanolamine/phosphocholine phosphatase OS=Gallus gallus GN=PHOSPHO1 PE=4 SV=1 - [F1NYY5_CHICK]	0.00	1.057	30.6	6.67
R4GJN7	Histone H2A OS=Gallus gallus GN=LOC100857391 PE=3 SV=1 - [R4GJN7_CHICK]	687.52	1.057	15.0	10.87
Q5ZLE6	Eukaryotic translation initiation factor 3 subunit H OS=Gallus gallus GN=EIF3H PE=2 SV=1 - [EIF3H_CHICK]	107.86	1.057	39.5	6.47
Q8JG64	Protein disulfide-isomerase A3 OS=Gallus gallus GN=PDIA3 PE=2 SV=1 - [PDIA3_CHICK]	1732.89	1.056	56.1	6.02
Q6Q122	NADH dehydrogenase [ubiquinone] 1 subunit C2 OS=Gallus gallus GN=NDUFC2 PE=3 SV=1 - [Q6Q122_CHICK]	0.00	1.056	11.0	9.25
R9PXM0	Cyclin-dependent kinase 9 (Fragment) OS=Gallus gallus GN=CDK9 PE=4 SV=1 - [R9PXM0_CHICK]	23.50	1.056	39.4	9.23
P07850	Sulfite oxidase OS=Gallus gallus GN=SUOX PE=1 SV=3 - [SUOX_CHICK]	0.00	1.056	50.2	6.18

Q7SX83	CAMP response element-binding protein OS=Gallus gallus GN=CREB1 PE=2 SV=1 - [Q7SX83_CHICK]	68.98	1.056	35.1	5.27
Q5ZM11	Arginine--tRNA ligase, cytoplasmic OS=Gallus gallus GN=RARS PE=2 SV=1 - [SYRC_CHICK]	183.09	1.056	75.4	7.11
Q03669-2	Isoform 2 of Sarcoplasmic/endoplasmic reticulum calcium ATPase 2 OS=Gallus gallus GN=ATP2A2 - [AT2A2_CHICK]	118.88	1.056	109.7	5.36
F1P442	NEDD8-activating enzyme E1 regulatory subunit OS=Gallus gallus GN=NAE1 PE=4 SV=2 - [F1P442_CHICK]	51.43	1.055	60.4	5.58
Q90623-2	Isoform 2 of Protein phosphatase 1 regulatory subunit 12A OS=Gallus gallus GN=PPP1R12A - [MYPT1_CHICK]	70.78	1.054	106.9	5.45
Q5ZHZ0	Spliceosome RNA helicase DDX39B OS=Gallus gallus GN=DDX39B PE=2 SV=1 - [DX39B_CHICK]	212.37	1.054	49.0	5.67
Q5ZKD1	Choline/ethanolaminephosphotransferase 1 OS=Gallus gallus GN=CEPT1 PE=2 SV=2 - [CEPT1_CHICK]	0.00	1.054	46.1	8.00
E1BVX9	Signal recognition particle subunit SRP72 OS=Gallus gallus GN=SRP72 PE=3 SV=1 - [E1BVX9_CHICK]	146.44	1.054	74.4	9.20
P47838	40S ribosomal protein S6 OS=Gallus gallus GN=RPS6 PE=2 SV=1 - [RS6_CHICK]	104.68	1.054	28.6	10.83
F1NDA0	Glycylpeptide N-tetradecanoyltransferase OS=Gallus gallus GN=NMT1 PE=3 SV=2 - [F1NDA0_CHICK]	0.00	1.054	56.8	8.57
P05625	RAF proto-oncogene serine/threonine-protein kinase OS=Gallus gallus GN=RAF1 PE=2 SV=1 - [RAF1_CHICK]	0.00	1.053	73.1	9.22
Q5F3X4	116 kDa U5 small nuclear ribonucleoprotein component OS=Gallus gallus GN=EFTUD2 PE=2 SV=1 -	174.73	1.052	109.4	5.00

	[U5S1_CHICK]				
Q5ZHV7	SPRY domain-containing protein 7 OS=Gallus gallus GN=SPRYD7 PE=2 SV=1 - [SPRY7_CHICK]	32.37	1.052	21.7	6.77
E1BYM1	mRNA cap guanine-N7 methyltransferase OS=Gallus gallus GN=RNMT PE=3 SV=1 - [E1BYM1_CHICK]	75.87	1.052	47.5	5.62
P0CB50	Peroxiredoxin-1 OS=Gallus gallus GN=PRDX1 PE=1 SV=1 - [PRDX1_CHICK]	558.55	1.051	22.3	8.10
Q66VY4	Splicing factor 3a subunit 2 OS=Gallus gallus GN=SF3A2 PE=2 SV=1 - [Q66VY4_CHICK]	0.00	1.051	35.1	9.64
O42146	Metalloproteinase inhibitor 2 OS=Gallus gallus GN=TIMP2 PE=2 SV=1 - [TIMP2_CHICK]	28.33	1.051	24.3	7.47
Q5ZKQ7	Glucose-induced degradation protein 8 homolog OS=Gallus gallus GN=GID8 PE=2 SV=1 - [GID8_CHICK]	67.88	1.051	26.8	4.92
F1NYW0	Tyrosine-protein phosphatase non-receptor type OS=Gallus gallus GN=PTPN2 PE=3 SV=2 - [F1NYW0_CHICK]	85.78	1.051	48.8	8.85
Q9YH14	Progesterone receptor binding protein OS=Gallus gallus GN=rbf PE=4 SV=1 - [Q9YH14_CHICK]	69.21	1.051	8.3	8.37
Q9DFZ3	Protein Churchill OS=Gallus gallus GN=CHURC1 PE=3 SV=1 - [CHUR_CHICK]	15.86	1.050	12.5	5.20
E1C3D2	Septin-2 OS=Gallus gallus GN=SEPT2 PE=3 SV=1 - [E1C3D2_CHICK]	241.13	1.050	41.6	6.30
Q5ZK71	DNA topoisomerase OS=Gallus gallus GN=TOP3B PE=2 SV=1 - [Q5ZK71_CHICK]	0.00	1.050	96.6	8.09
F1NDN4	Structural maintenance of chromosomes protein OS=Gallus gallus GN=SMC4 PE=3 SV=1 - [F1NDN4_CHICK]	26.17	1.050	145.6	6.40

F6SU35	Ribosomal protein OS=Gallus gallus GN=RPL10A PE=3 SV=1 - [F6SU35_CHICK]	145.45	1.050	24.7	9.88
F1NNZ9	Protein kinase C and casein kinase substrate in neurons protein 2 OS=Gallus gallus GN=PACSIN2 PE=4 SV=1 - [F1NNZ9_CHICK]	82.36	1.049	51.7	5.34
P62758	Neurocalcin-delta OS=Gallus gallus GN=NCALD PE=2 SV=2 - [NCALD_CHICK]	0.00	1.049	22.2	5.35
E1C0F1	Dolichyl-diphosphooligosaccharide--protein glycosyltransferase subunit 1 (Fragment) OS=Gallus gallus GN=RPN1 PE=4 SV=2 - [E1C0F1_CHICK]	293.79	1.048	60.6	6.89
P35458	Dynactin subunit 1 OS=Gallus gallus GN=DCTN1 PE=2 SV=2 - [DCTN1_CHICK]	99.55	1.048	135.5	6.54
Q5ZLN4	Flap endonuclease 1 OS=Gallus gallus GN=FEN1 PE=2 SV=1 - [FEN1_CHICK]	238.65	1.048	43.0	8.12
F1NXG8	Pleckstrin homology domain-containing family B member 2 OS=Gallus gallus GN=PLEKHB2 PE=4 SV=1 - [F1NXG8_CHICK]	43.61	1.048	25.4	6.28
F1NTR5	Putative Polycomb group protein ASXL2 OS=Gallus gallus GN=ASXL2 PE=4 SV=2 - [F1NTR5_CHICK]	36.36	1.047	142.7	8.31
P62207	Serine/threonine-protein phosphatase PP1-beta catalytic subunit OS=Gallus gallus GN=PPP1CB PE=1 SV=3 - [PP1B_CHICK]	59.43	1.047	37.2	6.19
E1C6T4	Serine/threonine-protein kinase D OS=Gallus gallus GN=PRKD3 PE=3 SV=1 - [E1C6T4_CHICK]	0.00	1.047	100.3	6.73
E1C231	E3 ubiquitin-protein ligase listerin OS=Gallus gallus GN=LTN1 PE=3 SV=1 - [LTN1_CHICK]	0.00	1.047	199.3	6.05
E1C453	Heterogeneous nuclear ribonucleoprotein K OS=Gallus gallus GN=HNRNPK PE=4 SV=1 -	486.97	1.046	47.1	6.47

	[E1C453_CHICK]				
G1K303	Hydroxyacylglutathione hydrolase, mitochondrial OS=Gallus gallus GN=HAGH PE=3 SV=2 - [G1K303_CHICK]	0.00	1.046	34.2	7.81
Q5ZLG1	Ras-related protein Rab-18 OS=Gallus gallus GN=RAB18 PE=2 SV=1 - [RAB18_CHICK]	116.11	1.046	22.9	5.36
Q800K9	Surfeit locus protein 4 OS=Gallus gallus GN=SURF4 PE=2 SV=1 - [SURF4_CHICK]	30.80	1.045	30.6	7.78
E1C7T8	Coatomer subunit gamma OS=Gallus gallus GN=COPG2 PE=3 SV=1 - [E1C7T8_CHICK]	139.37	1.045	97.5	5.69
F1NH33	Aldehyde dehydrogenase OS=Gallus gallus GN=ALDH3A2 PE=3 SV=1 - [F1NH33_CHICK]	70.03	1.045	54.1	8.02
Q5ZJ43	Exocyst complex component 8 OS=Gallus gallus GN=EXOC8 PE=2 SV=1 - [EXOC8_CHICK]	23.98	1.044	80.4	5.30
P14105	Myosin-9 OS=Gallus gallus GN=MYH9 PE=2 SV=1 - [MYH9_CHICK]	1857.92	1.044	226.4	5.57
Q90ZH5	SAP18 OS=Gallus gallus GN=chSAP18 PE=1 SV=1 - [Q90ZH5_CHICK]	0.00	1.044	17.6	9.35
F1P0B0	Integrin alpha-6 (Fragment) OS=Gallus gallus GN=ITGA6 PE=3 SV=2 - [F1P0B0_CHICK]	47.16	1.044	115.0	6.73
E1BR08	Histone H2A OS=Gallus gallus GN=H2AFY2 PE=3 SV=1 - [E1BR08_CHICK]	23.89	1.044	40.2	9.70
Q5F3Z7	CTD small phosphatase-like protein 2 OS=Gallus gallus GN=CTDSPL2 PE=2 SV=2 - [CTSL2_CHICK]	0.00	1.044	52.8	6.42
Q98SN3	Aryl hydrocarbon receptor nuclear translocator OS=Gallus gallus GN=ARNT PE=2 SV=1 - [Q98SN3_CHICK]	0.00	1.044	87.6	6.49

R9PXN4	Alpha-actinin-1 (Fragment) OS=Gallus gallus GN=ACTN1 PE=4 SV=1 - [R9PXN4_CHICK]	822.90	1.043	101.8	5.38
Q6XFR0	CD47 glycoprotein isoform B OS=Gallus gallus GN=CD47 PE=2 SV=1 - [Q6XFR0_CHICK]	0.00	1.043	33.2	8.28
F1NJ40	ATP-dependent RNA helicase DDX42 OS=Gallus gallus GN=DDX42 PE=4 SV=2 - [F1NJ40_CHICK]	35.18	1.042	103.0	6.76
Q5ZJW4	Vesicle-trafficking protein SEC22b OS=Gallus gallus GN=SEC22B PE=2 SV=1 - [SC22B_CHICK]	197.04	1.042	24.7	8.72
R4GHN5	Drebrin (Fragment) OS=Gallus gallus GN=DBN1 PE=4 SV=1 - [R4GHN5_CHICK]	158.16	1.042	61.3	4.55
Q5ZM18	Nucleolar GTP-binding protein 1 OS=Gallus gallus GN=GTPBP4 PE=2 SV=1 - [Q5ZM18_CHICK]	28.91	1.042	73.9	9.52
F1NG39	Unconventional myosin-Ic OS=Gallus gallus GN=MYO1C PE=4 SV=1 - [F1NG39_CHICK]	380.28	1.042	118.8	9.16
Q08705	Transcriptional repressor CTCF OS=Gallus gallus GN=CTCF PE=1 SV=1 - [CTCF_CHICK]	0.00	1.041	82.8	6.86
H9L3J2	U4/U6 small nuclear ribonucleoprotein Prp3 OS=Gallus gallus GN=PRPF3 PE=4 SV=1 - [H9L3J2_CHICK]	68.05	1.041	77.3	9.54
Q9W719	Hypoxanthine-guanine phosphoribosyltransferase OS=Gallus gallus GN=HPRT1 PE=2 SV=1 - [HPRT_CHICK]	185.88	1.041	24.6	7.42
E1C502	Autophagy-related protein 3 OS=Gallus gallus GN=ATG3 PE=3 SV=1 - [E1C502_CHICK]	0.00	1.041	35.6	4.72
Q5F384	Protein YIPF3 OS=Gallus gallus GN=YIPF3 PE=2 SV=1 - [YIPF3_CHICK]	25.29	1.041	36.9	5.34
Q7SX63	Heat shock protein 70 OS=Gallus gallus GN=HSP70 PE=3 SV=1 - [Q7SX63_CHICK]	1189.15	1.041	69.9	5.86
P42558	GTP-binding nuclear protein Ran OS=Gallus gallus GN=RAN PE=2 SV=1 - [RAN_CHICK]	132.74	1.041	24.4	7.88

P11501	Heat shock protein HSP 90-alpha OS=Gallus gallus GN=HSP90AA1 PE=3 SV=3 - [HS90A_CHICK]	1260.43	1.040	84.0	5.08
E1BUM7	Alpha-(1,6)-fucosyltransferase OS=Gallus gallus GN=FUT8 PE=3 SV=1 - [E1BUM7_CHICK]	0.00	1.040	66.3	7.05
Q5ZKY9	Mediator of RNA polymerase II transcription subunit 20 OS=Gallus gallus GN=MED20 PE=2 SV=1 - [MED20_CHICK]	0.00	1.039	23.3	6.96
R4GMG2	Ribosome biogenesis protein BOP1 OS=Gallus gallus GN=BOP1 PE=3 SV=1 - [R4GMG2_CHICK]	0.00	1.039	74.0	8.57
F1NQU7	Eukaryotic translation initiation factor 4 gamma 2 (Fragment) OS=Gallus gallus GN=EIF4G2 PE=4 SV=1 - [F1NQU7_CHICK]	92.22	1.039	97.5	7.02
F1P4Y3	Serine/threonine-protein kinase 4 OS=Gallus gallus GN=STK4 PE=4 SV=1 - [F1P4Y3_CHICK]	0.00	1.039	55.4	5.11
F1NBT0	Serine/threonine-protein kinase 10 OS=Gallus gallus GN=STK10 PE=3 SV=2 - [STK10_CHICK]	0.00	1.038	112.4	7.42
Q90734	Alpha-actinin-4 OS=Gallus gallus GN=ACTN4 PE=1 SV=1 - [ACTN4_CHICK]	705.36	1.038	104.1	5.26
E1C1D1	Annexin OS=Gallus gallus GN=ANXA7 PE=3 SV=2 - [E1C1D1_CHICK]	81.85	1.038	49.3	5.91
P17785	Annexin A2 OS=Gallus gallus GN=ANXA2 PE=1 SV=2 - [ANXA2_CHICK]	803.51	1.038	38.6	7.33
P24367	Peptidyl-prolyl cis-trans isomerase B OS=Gallus gallus GN=PPIB PE=2 SV=1 - [PPIB_CHICK]	237.29	1.038	22.4	9.39
F1NZP0	Twinfilin-2 (Fragment) OS=Gallus gallus GN=TWF2 PE=4 SV=2 - [F1NZP0_CHICK]	99.21	1.038	39.1	6.55
P23007	Citrate synthase, mitochondrial OS=Gallus gallus GN=CS PE=1 SV=1 - [CISY_CHICK]	94.28	1.037	47.3	8.22

F1P165	Palmitoyltransferase (Fragment) OS=Gallus gallus GN=ZDHHC20 PE=3 SV=2 - [F1P165_CHICK]	0.00	1.037	41.6	7.55
I7LRG5	HN1 OS=Gallus gallus GN=HN1 PE=2 SV=1 - [I7LRG5_CHICK]	21.43	1.037	16.1	4.91
F1NXK0	Mini-chromosome maintenance complex-binding protein OS=Gallus gallus GN=MCMBP PE=4 SV=2 - [F1NXK0_CHICK]	61.35	1.037	67.6	5.29
F7BWA2	Signal transducer and activator of transcription OS=Gallus gallus GN=STAT1 PE=3 SV=1 - [F7BWA2_CHICK]	49.75	1.036	86.4	7.75
F1NWF6	Asparagine synthetase OS=Gallus gallus GN=ASNS PE=4 SV=1 - [F1NWF6_CHICK]	258.56	1.036	64.0	6.77
Q5ZLP8	Insulin-like growth factor 2 mRNA-binding protein 3 OS=Gallus gallus GN=IGF2BP3 PE=2 SV=1 - [IF2B3_CHICK]	158.92	1.036	64.3	8.94
F1NTY0	Zinc finger CCCH domain-containing protein 14 OS=Gallus gallus GN=ZC3H14 PE=4 SV=2 - [F1NTY0_CHICK]	42.31	1.035	81.6	7.36
E1BYT4	Phosphatidylinositol 3-kinase OS=Gallus gallus PE=3 SV=2 - [E1BYT4_CHICK]	27.54	1.035	105.9	7.05
Q8QGH2	Small ubiquitin-related modifier 1 OS=Gallus gallus GN=SUMO1 PE=3 SV=1 - [SUMO1_CHICK]	50.89	1.034	11.5	5.52
Q789A6	Nonmuscle myosin heavy chain OS=Gallus gallus GN=MYH10 PE=2 SV=1 - [Q789A6_CHICK]	683.47	1.034	228.9	5.50
E1C494	60S ribosome subunit biogenesis protein NIP7 homolog (Fragment) OS=Gallus gallus GN=NIP7 PE=3 SV=2 - [E1C494_CHICK]	0.00	1.034	21.1	9.39

Q90838	Leucine zipper protein OS=Gallus gallus GN=PTRF PE=2 SV=1 - [Q90838_CHICK]	450.33	1.034	41.3	5.64
F1N8Q2	Microtubule-associated protein RP/EB family member 2 (Fragment) OS=Gallus gallus GN=MAPRE2 PE=4 SV=2 - [F1N8Q2_CHICK]	95.13	1.033	33.6	6.24
F1N833	Prohibitin-2 (Fragment) OS=Gallus gallus GN=PHB2 PE=4 SV=2 - [F1N833_CHICK]	115.77	1.033	30.6	9.74
F1NYI3	tRNA-splicing ligase RtcB homolog OS=Gallus gallus GN=C1H22orf28 PE=3 SV=2 - [F1NYI3_CHICK]	193.32	1.033	55.2	7.24
F1P0P4	Signal recognition particle subunit SRP68 OS=Gallus gallus GN=SRP68 PE=3 SV=1 - [F1P0P4_CHICK]	69.84	1.033	68.8	8.43
F1NLW5	Casein kinase I isoform epsilon OS=Gallus gallus GN=CSNK1E PE=4 SV=2 - [F1NLW5_CHICK]	33.92	1.033	47.0	9.79
F1NZ25	Eukaryotic translation initiation factor 3 subunit B OS=Gallus gallus GN=EIF3B PE=3 SV=2 - [F1NZ25_CHICK]	138.81	1.033	85.6	5.11
Q56I99	Mothers against decapentaplegic homolog 5 OS=Gallus gallus GN=SMAD5 PE=2 SV=1 - [SMAD5_CHICK]	0.00	1.033	52.2	7.71
E1BUM0	Peptidyl-prolyl cis-trans isomerase E OS=Gallus gallus GN=PPIE PE=3 SV=1 - [E1BUM0_CHICK]	38.06	1.033	33.3	5.60
F1NCI6	60S ribosomal protein L7 (Fragment) OS=Gallus gallus GN=RPL7 PE=3 SV=1 - [F1NCI6_CHICK]	268.65	1.032	28.7	10.83
Q5ZHX1	Ras-related protein Rap-1b OS=Gallus gallus GN=RAP1B PE=2 SV=1 - [RAP1B_CHICK]	68.11	1.032	20.8	5.78
I6L4M8	Phosphatase and actin regulator OS=Gallus gallus GN=PHACTR4 PE=3 SV=2 - [I6L4M8_CHICK]	33.44	1.032	78.2	6.96
F1NDH1	Arginyl-tRNA--protein transferase 1 OS=Gallus gallus GN=ATE1 PE=3 SV=1 - [F1NDH1_CHICK]	20.49	1.032	54.9	7.14

E1C8R1	Probable ATP-dependent RNA helicase DDX6 OS=Gallus gallus GN=DDX6 PE=4 SV=1 - [E1C8R1_CHICK]	86.47	1.032	54.4	8.78
F1NEB4	Protein transport protein Sec31A OS=Gallus gallus GN=SEC31A PE=4 SV=2 - [F1NEB4_CHICK]	123.55	1.032	133.2	6.28
F1NCN0	Transcription factor AP-1 OS=Gallus gallus GN=JUN PE=4 SV=1 - [F1NCN0_CHICK]	55.99	1.032	34.4	8.75
E1BSS3	Eukaryotic translation initiation factor 3 subunit A OS=Gallus gallus GN=EIF3A PE=3 SV=1 - [E1BSS3_CHICK]	321.63	1.031	163.2	7.15
Q9PV94	Small nuclear ribonucleoprotein-associated protein B' OS=Gallus gallus GN=SNRPB PE=2 SV=1 - [RSMB_CHICK]	98.51	1.031	24.6	11.19
Q5ZL77	Synembryn-A OS=Gallus gallus GN=RIC8A PE=2 SV=1 - [RIC8A_CHICK]	48.11	1.031	61.5	5.25
Q5F464	Lipoma-preferred partner homolog OS=Gallus gallus GN=LPP PE=2 SV=1 - [LPP_CHICK]	128.39	1.030	65.1	7.50
F1P375	Raftlin OS=Gallus gallus GN=RFTN1 PE=4 SV=1 - [F1P375_CHICK]	27.93	1.030	65.6	5.44
Q5ZLW3	Dymeclin OS=Gallus gallus GN=DYM PE=2 SV=1 - [DYM_CHICK]	0.00	1.030	76.1	6.07
E1C878	Glucosamine-6-phosphate deaminase 2 isoform 1 OS=Gallus gallus GN=GNPDA2 PE=2 SV=1 - [E1C878_CHICK]	0.00	1.029	31.1	6.60
A0SVH2	Tyrosine-protein kinase transmembrane receptor OS=Gallus gallus GN=ROR2 PE=2 SV=1 - [A0SVH2_CHICK]	0.00	1.029	104.5	6.42
Q6VN51	Protein phospatase 3 regulatory subunit B alpha isoform type 1 OS=Gallus gallus GN=PPP3R1 PE=2 SV=1	48.27	1.029	19.3	4.81

	- [Q6VN51_CHICK]				
Q5ZK03	Protein transport protein Sec23A OS=Gallus gallus GN=SEC23A PE=2 SV=1 - [SC23A_CHICK]	120.41	1.029	86.2	6.79
E1C6Q1	Pachytene checkpoint protein 2 homolog OS=Gallus gallus GN=TRIP13 PE=3 SV=1 - [PCH2_CHICK]	17.59	1.029	49.0	6.30
F1NED5	X-ray repair cross-complementing protein 5 (Fragment) OS=Gallus gallus GN=XRCC6 PE=4 SV=2 - [F1NED5_CHICK]	89.08	1.029	71.2	6.15
E1C9F6	Neurofibromin OS=Gallus gallus GN=NF1 PE=4 SV=1 - [E1C9F6_CHICK]	26.64	1.029	319.6	7.50
F1P3Q0	Nuclear distribution protein nudE-like 1 OS=Gallus gallus GN=NDEL1 PE=4 SV=2 - [F1P3Q0_CHICK]	56.60	1.029	37.0	5.38
O42484	17-beta-hydroxysteroid dehydrogenase type IV OS=Gallus gallus GN=HSD17B4 PE=2 SV=1 - [O42484_CHICK]	193.35	1.028	80.1	8.37
Q8UWC5	Nuclear protein matrin 3 OS=Gallus gallus GN=MATR3 PE=2 SV=1 - [Q8UWC5_CHICK]	206.08	1.028	100.7	6.15
Q5ZJV9	CCR4-NOT transcription complex subunit 7 OS=Gallus gallus GN=CNOT7 PE=2 SV=1 - [CNOT7_CHICK]	0.00	1.028	32.7	4.84
F1N9J4	60S ribosomal protein L22 (Fragment) OS=Gallus gallus GN=RPL22 PE=4 SV=1 - [F1N9J4_CHICK]	206.19	1.028	14.6	9.19
Q679P3	PDZ and LIM domain protein 7 OS=Gallus gallus GN=PDLIM7 PE=1 SV=1 - [PDLI7_CHICK]	57.94	1.027	45.7	8.70
Q5ZIP6	OTU domain-containing protein 6B OS=Gallus gallus GN=OTUD6B PE=2 SV=1 - [OTU6B_CHICK]	29.09	1.027	34.5	6.30
D2D3P4	Rab27a OS=Gallus gallus GN=Rab27a PE=2 SV=1 - [D2D3P4_CHICK]	76.73	1.027	25.1	5.36

Q5ZML3	Serine/arginine-rich splicing factor 1 OS=Gallus gallus GN=SRSF1 PE=1 SV=3 - [SRSF1_CHICK]	346.63	1.027	28.0	6.95
F1NQS2	Glutathione S-transferase OS=Gallus gallus GN=LOC396380 PE=3 SV=1 - [F1NQS2_CHICK]	58.54	1.027	26.3	9.03
E1C006	Proteasome subunit alpha type OS=Gallus gallus GN=PSMA2 PE=3 SV=1 - [E1C006_CHICK]	66.92	1.027	25.9	7.49
F1NE72	Lysine--tRNA ligase OS=Gallus gallus GN=KARS PE=3 SV=1 - [F1NE72_CHICK]	113.71	1.026	67.8	6.23
P84173	Prohibitin OS=Gallus gallus GN=PHB PE=1 SV=1 - [PHB_CHICK]	279.16	1.026	29.9	5.76
F1P394	DNA ligase OS=Gallus gallus GN=LIG3 PE=3 SV=2 - [F1P394_CHICK]	34.45	1.025	101.2	8.79
F1P2T2	NADPH--cytochrome P450 reductase OS=Gallus gallus GN=POR PE=3 SV=2 - [F1P2T2_CHICK]	145.16	1.025	76.9	5.60
Q5ZKC9	14-3-3 protein zeta OS=Gallus gallus GN=YWHAZ PE=2 SV=1 - [1433Z_CHICK]	661.87	1.025	27.8	4.79
F1NYQ3	Non-lysosomal glucosylceramidase OS=Gallus gallus GN=LOC100859407 PE=3 SV=2 - [F1NYQ3_CHICK]	59.47	1.025	96.0	6.40
P00368	Glutamate dehydrogenase 1, mitochondrial OS=Gallus gallus GN=GLUD1 PE=1 SV=1 - [DHE3_CHICK]	345.53	1.025	55.7	8.28
E1C3Y5	Protein transport protein Sec23A OS=Gallus gallus GN=SEC23A PE=4 SV=1 - [E1C3Y5_CHICK]	98.48	1.025	86.1	6.96
E1BVT3	Malate dehydrogenase OS=Gallus gallus GN=MDH2 PE=3 SV=1 - [E1BVT3_CHICK]	1291.26	1.024	35.6	8.56
F1NRM8	Endoplasmic reticulum resident protein 29 (Fragment) OS=Gallus gallus GN=ERP29 PE=4 SV=2 - [F1NRM8_CHICK]	207.13	1.024	29.1	8.22
F1NSQ4	Mitochondrial Rho GTPase OS=Gallus gallus GN=RHOT1 PE=3 SV=1 - [F1NSQ4_CHICK]	51.22	1.024	70.8	6.15

Q6IVA4	Ubiquitin-like modifier-activating enzyme 5 OS=Gallus gallus GN=UBA5 PE=2 SV=1 - [UBA5_CHICK]	29.64	1.024	44.1	4.84
F1NWP3	Heat shock cognate 71 kDa protein OS=Gallus gallus GN=HSPA8 PE=3 SV=1 - [F1NWP3_CHICK]	1443.98	1.024	70.8	5.52
Q5ZJJ8	Ubiquitin-like domain-containing CTD phosphatase 1 OS=Gallus gallus GN=UBLCP1 PE=2 SV=1 - [UBCP1_CHICK]	0.00	1.024	36.8	6.37
Q5F4B2	Switch-associated protein 70 OS=Gallus gallus GN=SWAP70 PE=2 SV=1 - [SWP70_CHICK]	39.60	1.024	68.4	6.34
F1NNL1	Transmembrane protein 231 OS=Gallus gallus GN=TMEM231 PE=3 SV=2 - [TM231_CHICK]	0.00	1.024	36.5	9.33
F1NJT2	Tyrosine-protein kinase receptor (Fragment) OS=Gallus gallus GN=INSR PE=3 SV=2 - [F1NJT2_CHICK]	0.00	1.024	153.3	6.14
Q5ZLC9	Golgin subfamily A member 7 OS=Gallus gallus GN=GOLGA7 PE=2 SV=1 - [GOGA7_CHICK]	93.14	1.023	15.8	7.05
E1BWX1	Annexin OS=Gallus gallus GN=ANXA6 PE=3 SV=2 - [E1BWX1_CHICK]	718.62	1.023	86.9	6.32
P67966	Cysteine and glycine-rich protein 1 OS=Gallus gallus GN=CSRP1 PE=1 SV=2 - [CSRP1_CHICK]	78.94	1.023	20.4	8.57
P98152	Transcription factor p65 OS=Gallus gallus GN=RELA PE=1 SV=1 - [TF65_CHICK]	33.80	1.022	60.0	5.94
F2Z4L5	60S ribosomal protein L7a (Fragment) OS=Gallus gallus GN=RPL7A PE=4 SV=1 - [F2Z4L5_CHICK]	677.44	1.022	29.8	10.51
Q8UVC3	Inversin OS=Gallus gallus GN=INVS PE=2 SV=2 - [INVS_CHICK]	0.00	1.022	122.5	9.16
Q8AWB8	Cohesin complex subunit OS=Gallus gallus GN=SMC3 PE=2 SV=1 - [Q8AWB8_CHICK]	118.33	1.022	141.5	7.06
Q90835	Elongation factor 1-alpha 1 OS=Gallus gallus GN=EEF1A PE=2 SV=1 - [EF1A_CHICK]	1710.40	1.022	50.1	9.01

E1BTY4	Sorting nexin OS=Gallus gallus GN=SNX9 PE=3 SV=1 - [E1BTY4_CHICK]	98.07	1.022	67.1	5.58
F1NC77	NEDD4-binding protein 1 OS=Gallus gallus GN=N4BP1 PE=4 SV=1 - [F1NC77_CHICK]	0.00	1.022	103.9	6.38
Q5ZMP6	AP-2 complex subunit mu OS=Gallus gallus GN=AP2M1 PE=2 SV=1 - [AP2M1_CHICK]	79.25	1.022	49.4	9.54
P63283	SUMO-conjugating enzyme UBC9 OS=Gallus gallus GN=UBE2I PE=2 SV=1 - [UBC9_CHICK]	83.15	1.022	18.0	8.66
H9L340	ATP synthase subunit beta (Fragment) OS=Gallus gallus GN=ATP5B PE=3 SV=2 - [H9L340_CHICK]	1517.98	1.021	52.9	5.21
F1NMV4	ATP-dependent RNA helicase SUPV3L1, mitochondrial OS=Gallus gallus GN=SUPV3L1 PE=4 SV=1 - [F1NMV4_CHICK]	0.00	1.021	88.7	8.06
Q5ZKV4	Cytosolic Fe-S cluster assembly factor NUBP2 OS=Gallus gallus GN=NUBP2 PE=2 SV=1 - [NUBP2_CHICK]	77.54	1.021	29.3	6.07
E1BRJ9	Coronin OS=Gallus gallus GN=CORO7 PE=3 SV=2 - [E1BRJ9_CHICK]	0.00	1.021	100.0	5.76
O73664	MSSP-1 OS=Gallus gallus GN=RBMS1 PE=2 SV=1 - [O73664_CHICK]	191.07	1.020	40.6	8.35
H9L1C2	Transaldolase OS=Gallus gallus GN=TALDO1 PE=3 SV=2 - [H9L1C2_CHICK]	120.55	1.020	37.6	7.47
Q5ZL19	Syntaxin-6 OS=Gallus gallus GN=STX6 PE=2 SV=1 - [STX6_CHICK]	28.37	1.020	29.1	4.93
P43347	Translationally-controlled tumor protein homolog OS=Gallus gallus GN=TPT1 PE=2 SV=1 - [TCTP_CHICK]	0.00	1.020	19.5	5.00
H9KZF8	ATP synthase subunit gamma OS=Gallus gallus GN=ATP5C1 PE=3 SV=2 - [H9KZF8_CHICK]	205.73	1.020	32.7	9.44

Q98932	Ras-related protein Rab-5C OS=Gallus gallus GN=RAB5C PE=1 SV=1 - [RAB5C_CHICK]	328.48	1.020	23.5	8.41
E1BYW9	Proteasome subunit beta type OS=Gallus gallus GN=PSMB3 PE=3 SV=1 - [E1BYW9_CHICK]	151.41	1.020	23.1	5.41
Q5ZK63	Activated RNA polymerase II transcriptional coactivator p15 OS=Gallus gallus GN=SUB1 PE=2 SV=1 - [TCP4_CHICK]	76.76	1.020	14.2	9.57
P15143	POU domain, class 2, transcription factor 1 OS=Gallus gallus GN=POU2F1 PE=2 SV=1 - [PO2F1_CHICK]	45.93	1.019	75.9	7.18
Q98TF6	60S ribosomal protein L36 OS=Gallus gallus GN=RPL36 PE=3 SV=1 - [RL36_CHICK]	49.72	1.019	12.3	11.34
E1BZR9	Tectonin beta-propeller repeat-containing protein 1 OS=Gallus gallus GN=TECPR1 PE=3 SV=1 - [TCPR1_CHICK]	0.00	1.019	131.7	6.11
Q5F371	Protein strawberry notch homolog 1 OS=Gallus gallus GN=SBNO1 PE=2 SV=1 - [SBNO1_CHICK]	44.27	1.019	137.1	6.48
Q5F398	Macrophage erythroblast attacher OS=Gallus gallus GN=MAEA PE=2 SV=1 - [MAEA_CHICK]	0.00	1.019	45.4	8.59
F1NC02	Proteasome subunit alpha type OS=Gallus gallus GN=PSMA4 PE=3 SV=2 - [F1NC02_CHICK]	86.05	1.019	29.5	7.33
F2Z4K7	40S ribosomal protein S3a OS=Gallus gallus GN=RPS3A PE=3 SV=1 - [F2Z4K7_CHICK]	145.07	1.019	29.8	9.72
Q5ZL12	Protein phosphatase 1 regulatory subunit 21 OS=Gallus gallus GN=PPP1R21 PE=2 SV=1 - [PPR21_CHICK]	0.00	1.019	88.8	7.83
P12003-1	Isoform 1 of Vinculin OS=Gallus gallus GN=VCL - [VINC_CHICK]	1523.89	1.018	116.9	6.33

Q5ZLH9	Protein CDV3 homolog OS=Gallus gallus GN=CDV3 PE=2 SV=1 - [CDV3_CHICK]	85.71	1.018	21.6	5.02
R4GKF3	Alpha-1,3-mannosyl-glycoprotein 4-beta-N-acetylglucosaminyltransferase C OS=Gallus gallus GN=MGAT4C PE=4 SV=1 - [R4GKF3_CHICK]	0.00	1.018	56.7	8.06
F1N9F8	Alcohol dehydrogenase [NADP(+)] OS=Gallus gallus GN=AKR1A1 PE=4 SV=1 - [F1N9F8_CHICK]	67.65	1.018	37.1	7.80
Q5ZML5	Protein LSM12 homolog OS=Gallus gallus GN=LSM12 PE=2 SV=1 - [LSM12_CHICK]	45.90	1.018	21.5	7.69
P13648	Lamin-A OS=Gallus gallus GN=LMNA PE=2 SV=1 - [LMNA_CHICK]	2150.18	1.018	73.1	6.95
Q07659	Fibroblast growth factor OS=Gallus gallus GN=bFGF PE=2 SV=1 - [Q07659_CHICK]	53.18	1.018	16.2	9.72
F1NY05	Hypoxia up-regulated protein 1 (Fragment) OS=Gallus gallus GN=HYOU1 PE=3 SV=2 - [F1NY05_CHICK]	455.92	1.018	108.3	5.17
Q5ZKI0	Calcium/calmodulin-dependent protein kinase type II delta chain OS=Gallus gallus GN=CAMK2D PE=2 SV=1 - [KCC2D_CHICK]	92.62	1.018	54.2	7.12
P41125	60S ribosomal protein L13 OS=Gallus gallus GN=RPL13 PE=2 SV=2 - [RL13_CHICK]	157.34	1.017	24.4	11.75
Q5ZLH4	Transmembrane protein 230 OS=Gallus gallus GN=TMEM230 PE=2 SV=1 - [TM230_CHICK]	0.00	1.017	13.2	9.28
E1C8Z0	Dolichyl-diphosphooligosaccharide--protein glycosyltransferase subunit 2 OS=Gallus gallus GN=RPN2 PE=4 SV=2 - [E1C8Z0_CHICK]	48.78	1.017	68.8	6.32
P00523	Proto-oncogene tyrosine-protein kinase Src OS=Gallus gallus GN=SRC PE=1 SV=4 - [SRC_CHICK]	37.06	1.017	60.0	7.65

H9L082	Bifunctional methylenetetrahydrofolate dehydrogenase/cyclohydrolase, mitochondrial OS=Gallus gallus GN=MTHFD2 PE=3 SV=1 - [H9L082_CHICK]	28.89	1.017	36.4	9.48
P14731	Lamin-B1 OS=Gallus gallus GN=LMNB1 PE=2 SV=1 - [LMNB1_CHICK]	172.48	1.017	66.5	5.20
P02607	Myosin light polypeptide 6 OS=Gallus gallus GN=MYL6 PE=1 SV=3 - [MYL6_CHICK]	234.67	1.016	17.0	4.59
E1C1R4	Ubiquitin carboxyl-terminal hydrolase 47 OS=Gallus gallus GN=USP47 PE=3 SV=1 - [UBP47_CHICK]	90.82	1.016	157.1	5.12
Q04584	Zyxin OS=Gallus gallus GN=ZYX PE=1 SV=1 - [ZYX_CHICK]	61.49	1.016	58.5	7.11
E1C4M6	Ubiquitin carboxyl-terminal hydrolase (Fragment) OS=Gallus gallus GN=USP14 PE=3 SV=2 - [E1C4M6_CHICK]	138.74	1.016	56.7	5.20
R4GFF8	Mitogen-activated protein kinase 6 OS=Gallus gallus GN=MAPK6 PE=4 SV=1 - [R4GFF8_CHICK]	0.00	1.016	79.6	5.15
P04268-5	Isoform 5 of Tropomyosin alpha-1 chain OS=Gallus gallus GN=TPM1 - [TPM1_CHICK]	307.02	1.016	32.8	4.75
F1NT50	Protein sidekick-1 OS=Gallus gallus GN=SDK1 PE=4 SV=2 - [F1NT50_CHICK]	0.00	1.016	158.6	5.63
Q9PVL6	Mitochondrial carrier homolog 2 OS=Gallus gallus GN=Mtch2 PE=2 SV=1 - [Q9PVL6_CHICK]	73.07	1.016	33.1	9.31
Q5ZHY5	STIP1 homology and U box-containing protein 1 OS=Gallus gallus GN=STUB1 PE=2 SV=1 - [CHIP_CHICK]	64.04	1.015	35.6	6.38
F1P1F4	Putative ATP-dependent RNA helicase DHX30 OS=Gallus gallus GN=DHX30 PE=4 SV=1 - [F1P1F4_CHICK]	38.24	1.015	137.7	7.96

F1NJ08	Vimentin OS=Gallus gallus GN=VIM PE=3 SV=1 - [F1NJ08_CHICK]	2495.66	1.015	53.2	5.21
F1P4D3	Glutamine-dependent NAD(+) synthetase OS=Gallus gallus GN=NADSYN1 PE=4 SV=1 - [F1P4D3_CHICK]	0.00	1.015	79.2	6.92
Q5ZIK2	PDZ domain-containing protein 11 OS=Gallus gallus GN=PDZD11 PE=2 SV=1 - [PDZ11_CHICK]	67.49	1.015	15.8	6.54
Q5ZLX2	Eukaryotic translation initiation factor 2 subunit 1 OS=Gallus gallus GN=EIF2S1 PE=2 SV=2 - [IF2A_CHICK]	259.42	1.014	36.2	5.14
Q5ZHW4	Ras-related protein Rab-5B OS=Gallus gallus GN=RAB5B PE=2 SV=1 - [RAB5B_CHICK]	214.75	1.014	23.6	8.13
H9L0H1	Transcription elongation factor SPT5 OS=Gallus gallus GN=SUPT5H PE=3 SV=2 - [H9L0H1_CHICK]	54.15	1.014	119.9	5.10
H9KZI4	Annexin OS=Gallus gallus PE=3 SV=2 - [H9KZI4_CHICK]	518.06	1.014	36.0	5.34
Q5ZKA4	Eukaryotic translation initiation factor 3 subunit J OS=Gallus gallus GN=EIF3J PE=2 SV=1 - [EIF3J_CHICK]	41.49	1.014	28.7	4.84
P09244	Tubulin beta-7 chain OS=Gallus gallus PE=2 SV=1 - [TBB7_CHICK]	932.17	1.014	49.6	4.89
Q90828	Cytoplasmic dynein 1 light intermediate chain 1 OS=Gallus gallus GN=DYNC1LI1 PE=1 SV=1 - [DC1L1_CHICK]	137.09	1.014	55.9	6.11
Q90965	Ras-related protein Rab-2A OS=Gallus gallus GN=RAB2A PE=2 SV=1 - [RAB2A_CHICK]	107.02	1.013	23.5	6.39
P25324	Thiosulfate sulfurtransferase OS=Gallus gallus GN=TST PE=1 SV=1 - [THTR_CHICK]	30.89	1.013	32.3	7.01

P02552	Tubulin alpha-1 chain (Fragment) OS=Gallus gallus PE=1 SV=1 - [TBA1_CHICK]	927.94	1.013	45.9	5.08
Q5ZLL7	WD repeat-containing protein 91 OS=Gallus gallus GN=WDR91 PE=2 SV=1 - [WDR91_CHICK]	0.00	1.013	83.4	6.62
F1NVQ6	Charged multivesicular body protein 1b OS=Gallus gallus GN=CHMP1B PE=4 SV=1 - [F1NVQ6_CHICK]	17.44	1.012	22.2	8.10
Q5F3U9	Sister chromatid cohesion protein PDS5 homolog B OS=Gallus gallus GN=PDS5B PE=2 SV=3 - [PDS5B_CHICK]	79.90	1.012	160.9	8.05
Q5ZKN2	Membrane-associated progesterone receptor component 1 OS=Gallus gallus GN=PGRMC1 PE=2 SV=3 - [PGRC1_CHICK]	34.01	1.012	21.3	4.81
P24798	Sodium/potassium-transporting ATPase subunit alpha-3 OS=Gallus gallus GN=ATP1A3 PE=2 SV=1 - [AT1A3_CHICK]	123.01	1.012	111.2	5.38
Q5ZK88	Paraspeckle component 1 OS=Gallus gallus GN=PSPC1 PE=2 SV=1 - [PSPC1_CHICK]	124.94	1.012	58.4	6.70
P60706	Actin, cytoplasmic 1 OS=Gallus gallus GN=ACTB PE=1 SV=1 - [ACTB_CHICK]	4027.53	1.011	41.7	5.48
Q6B842	Carnitine palmitoyltransferase I OS=Gallus gallus GN=CPT1A PE=2 SV=1 - [Q6B842_CHICK]	26.42	1.011	87.8	8.44
Q5ZJJ2	Replication protein A 70 kDa DNA-binding subunit OS=Gallus gallus GN=RPA1 PE=2 SV=1 - [RFA1_CHICK]	107.01	1.011	68.0	7.85
F1NGQ8	Vacuolar ATPase assembly integral membrane protein VMA21 OS=Gallus gallus GN=VMA21 PE=4 SV=1 - [F1NGQ8_CHICK]	53.59	1.011	11.5	7.24

R9PXN1	Eukaryotic initiation factor 4A-II OS=Gallus gallus GN=EIF4A2 PE=4 SV=1 - [R9PXN1_CHICK]	400.78	1.011	43.5	5.48
F6RX73	Proteasome subunit beta type (Fragment) OS=Gallus gallus GN=PSMB7 PE=3 SV=1 - [F6RX73_CHICK]	56.86	1.010	28.1	6.40
E1C612	Structural maintenance of chromosomes protein OS=Gallus gallus GN=SMC2 PE=3 SV=2 - [E1C612_CHICK]	41.96	1.010	134.8	8.62
Q07598	Non-specific lipid-transfer protein (Fragment) OS=Gallus gallus GN=SCP2 PE=2 SV=1 - [NLTP_CHICK]	480.36	1.010	58.7	8.19
E1C1E9	Acyl-coenzyme A oxidase OS=Gallus gallus GN=ACOX3 PE=3 SV=2 - [E1C1E9_CHICK]	24.09	1.010	79.9	7.72
Q5ZIV5	Programmed cell death protein 10 OS=Gallus gallus GN=PDCD10 PE=2 SV=1 - [PDC10_CHICK]	29.66	1.010	24.7	8.19
O13268	Proteasome subunit alpha type-7 OS=Gallus gallus GN=PSMA7 PE=2 SV=1 - [PSA7_CHICK]	57.10	1.010	28.1	8.84
O93378	Signal transducer and activator of transcription OS=Gallus gallus GN=Stat5 PE=2 SV=1 - [O93378_CHICK]	37.68	1.009	90.0	6.32
F1P4I9	Proteasomal ubiquitin receptor ADRM1 OS=Gallus gallus GN=ADRM1 PE=4 SV=1 - [F1P4I9_CHICK]	25.13	1.009	42.2	5.15
Q5ZK10	NSFL1 cofactor p47 OS=Gallus gallus GN=NSFL1C PE=2 SV=1 - [NSF1C_CHICK]	424.29	1.009	40.6	5.15
F1NVF0	Vacuolar protein sorting-associated protein 35 OS=Gallus gallus GN=VPS35 PE=3 SV=1 - [F1NVF0_CHICK]	123.48	1.009	91.7	5.58
Q9IAY5	Protein syndesmos OS=Gallus gallus GN=SDOS PE=1 SV=3 - [SDOS_CHICK]	290.14	1.009	33.3	6.13

Q5F414	RNA-binding protein PNO1 OS=Gallus gallus GN=PNO1 PE=2 SV=1 - [PNO1_CHICK]	0.00	1.009	26.4	9.92
H9KZ88	Guanine nucleotide-binding protein G(i) subunit alpha-2 (Fragment) OS=Gallus gallus GN=GNAI2 PE=4 SV=2 - [H9KZ88_CHICK]	182.67	1.008	39.0	5.76
Q5ZL42	Basic leucine zipper and W2 domain-containing protein 2 OS=Gallus gallus GN=BZW2 PE=2 SV=1 - [BZW2_CHICK]	51.12	1.008	47.8	8.27
F1P382	Vascular endothelial growth factor receptor 1 OS=Gallus gallus GN=FLT1 PE=3 SV=2 - [F1P382_CHICK]	32.61	1.008	149.3	9.19
F1P0Z7	ADP-ribosylation factor-like protein 8A OS=Gallus gallus GN=ARL8A PE=3 SV=2 - [F1P0Z7_CHICK]	62.79	1.007	21.5	8.43
Q6JHU8	Prolyl 3-hydroxylase 1 OS=Gallus gallus GN=LEPRE1 PE=1 SV=1 - [P3H1_CHICK]	145.49	1.007	81.6	5.50
F1NDU0	Protein CASC4 OS=Gallus gallus GN=CASC4 PE=4 SV=1 - [F1NDU0_CHICK]	70.46	1.007	42.5	6.15
F1P1X9	Casein kinase I isoform epsilon OS=Gallus gallus GN=DBT PE=3 SV=1 - [F1P1X9_CHICK]	40.25	1.007	54.1	8.56
F1NIK7	DNA-directed RNA polymerase OS=Gallus gallus GN=POLR3A PE=3 SV=1 - [F1NIK7_CHICK]	0.00	1.007	155.6	8.59
E1C834	Pericentriolar material 1 protein OS=Gallus gallus GN=PCM1 PE=4 SV=2 - [E1C834_CHICK]	0.00	1.007	222.4	5.05
Q5ZKW2	Protein LZIC OS=Gallus gallus GN=LZIC PE=2 SV=1 - [LZIC_CHICK]	70.08	1.007	21.5	4.88
Q5ZJV4	Mini-chromosome maintenance complex-binding protein OS=Gallus gallus GN=MCMBP PE=2 SV=2 - [MCMBP_CHICK]	62.71	1.007	71.9	5.64

Q90ZK7	Peptidyl-prolyl cis-trans isomerase OS=Gallus gallus GN=FKBP25 PE=2 SV=1 - [Q90ZK7_CHICK]	319.21	1.006	25.0	9.19
E1BSN7	Phosphorylase OS=Gallus gallus GN=PYGB PE=3 SV=2 - [E1BSN7_CHICK]	56.03	1.006	96.7	6.44
F1NLE6	Oxysterol-binding protein (Fragment) OS=Gallus gallus GN=OSBPL9 PE=3 SV=2 - [F1NLE6_CHICK]	22.12	1.006	83.2	5.88
Q5ZIP4	5'-3' exoribonuclease 2 OS=Gallus gallus GN=XRN2 PE=2 SV=1 - [XRN2_CHICK]	0.00	1.006	108.5	7.61
F1NT44	Translin OS=Gallus gallus GN=TSN PE=4 SV=1 - [F1NT44_CHICK]	56.91	1.006	26.0	6.71
Q5ZLC7	Microtubule-associated protein RP/EB family member 1 OS=Gallus gallus GN=MAPRE1 PE=2 SV=1 - [MARE1_CHICK]	163.83	1.006	29.1	5.14
F6SU00	Annexin OS=Gallus gallus GN=ANXA11 PE=3 SV=1 - [F6SU00_CHICK]	60.49	1.006	52.7	8.41
E1BXG0	Peptidyl-prolyl cis-trans isomerase OS=Gallus gallus GN=PPIH PE=3 SV=1 - [E1BXG0_CHICK]	54.56	1.006	19.2	8.07
R4GJB9	Receptor-type tyrosine-protein phosphatase eta OS=Gallus gallus GN=PTPRJ PE=4 SV=1 - [R4GJB9_CHICK]	0.00	1.006	130.1	4.97
P17924-2	Isoform 2 of Nuclear factor 1 B-type OS=Gallus gallus GN=NFIB - [NFIB_CHICK]	0.00	1.006	47.4	8.88
Q5F3T7-2	Isoform 2 of CUGBP Elav-like family member 1 OS=Gallus gallus GN=CELF1 - [CELF1_CHICK]	32.47	1.005	51.6	8.40
F1NEF8	NHL repeat-containing protein 2 OS=Gallus gallus GN=NHLRC2 PE=4 SV=2 - [F1NEF8_CHICK]	0.00	1.005	79.5	5.96
P17790-2	Isoform 2 of Basigin OS=Gallus gallus GN=BSG - [BASI_CHICK]	133.12	1.005	29.2	6.40

P57788	Monocarboxylate transporter 4 OS=Gallus gallus GN=SLC16A3 PE=2 SV=1 - [MOT4_CHICK]	79.67	1.005	51.0	7.39
F1NKT5	Transcription factor BTF3 homolog 4 OS=Gallus gallus GN=BTF3L4 PE=4 SV=1 - [F1NKT5_CHICK]	43.80	1.005	17.3	6.35
F1NZ86	Stress-70 protein, mitochondrial OS=Gallus gallus GN=HSPA9 PE=3 SV=1 - [F1NZ86_CHICK]	1034.16	1.005	73.1	6.43
P19352-2	Isoform 2 of Tropomyosin beta chain OS=Gallus gallus GN=TPM2 - [TPM2_CHICK]	211.59	1.005	32.8	4.69
H9L0U6	Proteasome subunit beta type OS=Gallus gallus GN=PSMB4 PE=3 SV=1 - [H9L0U6_CHICK]	58.62	1.004	28.1	5.97
Q08200	60S ribosomal protein L10 (Fragment) OS=Gallus gallus GN=RPL10 PE=2 SV=1 - [RL10_CHICK]	113.82	1.004	24.0	10.13
F1NGA2	ATP synthase subunit alpha OS=Gallus gallus GN=ATP5A1W PE=3 SV=2 - [F1NGA2_CHICK]	697.85	1.004	60.0	8.97
F1NG89	Ubiquitin carboxyl-terminal hydrolase (Fragment) OS=Gallus gallus GN=USP10 PE=3 SV=2 - [F1NG89_CHICK]	91.81	1.004	85.7	5.05
Q5ZIK9	Coatomer subunit epsilon OS=Gallus gallus GN=COPE PE=2 SV=1 - [COPE_CHICK]	163.44	1.004	34.3	5.14
Q5ZKD5	RRP12-like protein OS=Gallus gallus GN=RRP12 PE=2 SV=1 - [RRP12_CHICK]	51.87	1.004	144.0	8.32
F1NV49	ATP-dependent RNA helicase DDX1 OS=Gallus gallus GN=DDX1 PE=4 SV=2 - [F1NV49_CHICK]	142.17	1.003	81.8	7.27
F1N868	Tyrosine-protein kinase receptor OS=Gallus gallus GN=PDGFRB PE=3 SV=2 - [F1N868_CHICK]	24.64	1.003	119.2	5.43
Q9YH06	High mobility group protein B1 OS=Gallus gallus GN=HMGB1 PE=1 SV=1 - [HMGB1_CHICK]	287.11	1.003	24.9	5.74
Q9IAR7	TvbS1 OS=Gallus gallus GN=tvb PE=2 SV=1 - [Q9IAR7_CHICK]	42.16	1.003	41.5	6.61

F1N9H8	Protein PRRC1 OS=Gallus gallus GN=PRRC1 PE=4 SV=2 - [F1N9H8_CHICK]	57.73	1.003	45.9	5.80
F1NWD5	Leucine-rich repeat-containing protein 40 OS=Gallus gallus GN=LRRC40 PE=4 SV=1 - [F1NWD5_CHICK]	0.00	1.003	67.4	7.15
E1C078	Aldehyde dehydrogenase OS=Gallus gallus GN=LOC428812 PE=3 SV=2 - [E1C078_CHICK]	0.00	1.003	56.0	7.21
F1NPD3	60S ribosomal protein L18a (Fragment) OS=Gallus gallus GN=RPL18A PE=3 SV=2 - [F1NPD3_CHICK]	127.37	1.002	21.2	10.81
Q5ZLG8	Transmembrane anterior posterior transformation protein 1 homolog OS=Gallus gallus GN=TAPT1 PE=2 SV=2 - [TAPT1_CHICK]	0.00	1.002	65.7	8.47
F1N9P9	KRR1 small subunit processome component OS=Gallus gallus GN=KRR1 PE=3 SV=2 - [F1N9P9_CHICK]	0.00	1.002	43.1	9.94
E1C3A9	Vigilin OS=Gallus gallus GN=HDLBP PE=4 SV=1 - [E1C3A9_CHICK]	442.29	1.002	141.5	6.83
Q5F425	Protein lin-7 homolog C OS=Gallus gallus GN=LIN7C PE=1 SV=1 - [LIN7C_CHICK]	67.19	1.002	21.8	8.43
F1P399	Isoleucine--tRNA ligase, mitochondrial OS=Gallus gallus GN=IARS2 PE=3 SV=1 - [F1P399_CHICK]	110.05	1.002	111.9	6.89
Q5ZK95	Coiled-coil domain-containing protein 43 OS=Gallus gallus GN=CCDC43 PE=2 SV=1 - [CCD43_CHICK]	53.45	1.001	24.6	5.11
F1NY37	Acyl-coenzyme A oxidase OS=Gallus gallus GN=ACOX1 PE=3 SV=1 - [F1NY37_CHICK]	0.00	1.001	74.5	7.37
F1NK98	Tubulin-specific chaperone D OS=Gallus gallus GN=TBCD PE=4 SV=2 - [F1NK98_CHICK]	107.53	1.001	113.6	6.07
F1NQD9	Radixin (Fragment) OS=Gallus gallus GN=RDX PE=4 SV=2 - [F1NQD9_CHICK]	416.65	1.001	68.4	6.18
A5HNF6	Myeloid differentiation primary response protein MyD88 OS=Gallus gallus GN=MYD88 PE=1 SV=1 -	75.75	1.001	33.8	6.27

	[MYD88_CHICK]				
Q00944-2	Isoform 2 of Focal adhesion kinase 1 OS=Gallus gallus GN=PTK2 - [FAK1_CHICK]	0.00	1.001	40.0	6.34
F1NNQ3	Vacuolar protein sorting-associated protein 29 (Fragment) OS=Gallus gallus GN=VPS29 PE=4 SV=1 - [F1NNQ3_CHICK]	56.39	1.001	20.7	7.05
E1BSJ2	40S ribosomal protein S21 OS=Gallus gallus GN=RPS21 PE=3 SV=2 - [E1BSJ2_CHICK]	48.89	0.999	9.1	8.50
F1N8N7	Integrin beta OS=Gallus gallus GN=ITGB1 PE=3 SV=1 - [F1N8N7_CHICK]	331.42	0.999	88.6	5.25
F1NRB7	Sodium/potassium-transporting ATPase subunit beta-1 OS=Gallus gallus GN=ATP1B1 PE=3 SV=2 - [F1NRB7_CHICK]	29.41	0.999	28.5	5.82
F1NPU8	High mobility group protein B2 OS=Gallus gallus GN=HMGB2 PE=4 SV=1 - [F1NPU8_CHICK]	37.10	0.999	23.8	8.38
E1C458	CTP synthase OS=Gallus gallus GN=CTPS PE=3 SV=2 - [E1C458_CHICK]	23.23	0.999	69.7	7.39
Q5F3W6	14-3-3 protein gamma OS=Gallus gallus GN=YWHAG PE=1 SV=1 - [1433G_CHICK]	441.19	0.999	28.2	4.93
E1BXJ0	Protein-L-isoaspartate O-methyltransferase OS=Gallus gallus GN=LOC423008 PE=3 SV=2 - [E1BXJ0_CHICK]	63.69	0.999	27.1	6.89
Q7T0L4	Telomeric repeat-binding factor 2-interacting protein 1 OS=Gallus gallus GN=TERF2IP PE=1 SV=1 - [TE2IP_CHICK]	0.00	0.999	36.2	5.39
F1NCB0	DNA-directed RNA polymerase OS=Gallus gallus GN=POLR2B PE=3 SV=1 - [F1NCB0_CHICK]	81.20	0.999	133.7	6.95

E1BZ19	DNA topoisomerase 2 OS=Gallus gallus GN=TOP2B PE=3 SV=2 - [E1BZ19_CHICK]	80.49	0.999	183.2	8.28
Q5ZK40	SWI/SNF-related matrix-associated actin-dependent regulator of chromatin subfamily B member 1 OS=Gallus gallus GN=SMARCB1 PE=2 SV=1 - [SNF5_CHICK]	0.00	0.999	44.5	6.18
O13016	Tyrosine-protein phosphatase non-receptor type 1 OS=Gallus gallus GN=PTPN1 PE=1 SV=1 - [PTN1_CHICK]	119.64	0.998	50.3	6.07
Q8AXV0	Endophilin-A2 OS=Gallus gallus GN=SH3GL1 PE=1 SV=1 - [SH3G1_CHICK]	144.21	0.998	41.7	5.44
F1NN74	Anoctamin OS=Gallus gallus GN=TMEM16E PE=3 SV=2 - [F1NN74_CHICK]	38.85	0.998	106.4	6.33
R9PXM7	Coatomer subunit delta (Fragment) OS=Gallus gallus GN=ARCN1 PE=4 SV=1 - [R9PXM7_CHICK]	218.70	0.998	57.0	6.07
Q5F4B3	Trimethyllysine dioxygenase, mitochondrial OS=Gallus gallus GN=TMLHE PE=2 SV=1 - [TMLH_CHICK]	59.53	0.998	48.6	7.81
F1NRU9	Phosphatidylcholine:ceramide cholinephosphotransferase 1 (Fragment) OS=Gallus gallus GN=SGMS1 PE=4 SV=2 - [F1NRU9_CHICK]	39.95	0.998	48.9	8.76
F1P458	Sulfhydryl oxidase (Fragment) OS=Gallus gallus GN=QSOX2 PE=3 SV=2 - [F1P458_CHICK]	46.16	0.998	78.5	8.28
F1NR65	Charged multivesicular body protein 2b OS=Gallus gallus GN=CHMP2B PE=4 SV=1 - [F1NR65_CHICK]	0.00	0.998	24.0	8.75
F1NVH2	Tubulin-specific chaperone A OS=Gallus gallus GN=TBCA PE=4 SV=1 - [F1NVH2_CHICK]	32.17	0.998	12.7	5.44
P27003	Protein S100-A10 OS=Gallus gallus GN=S100A10 PE=3 SV=2 - [S10AA_CHICK]	227.10	0.998	11.3	7.39

P12902	Non-histone chromosomal protein HMG-14A OS=Gallus gallus PE=3 SV=2 - [HM14A_CHICK]	0.00	0.997	11.3	9.48
Q5ZIN0	Monoacylglycerol lipase ABHD12 OS=Gallus gallus GN=ABHD12 PE=2 SV=1 - [ABD12_CHICK]	0.00	0.997	43.8	8.91
Q5ZL38	SAGA-associated factor 29 homolog OS=Gallus gallus GN=CCDC101 PE=2 SV=1 - [SGF29_CHICK]	43.95	0.997	33.1	8.10
O42184	CAP-Gly domain-containing linker protein 1 OS=Gallus gallus GN=CLIP1 PE=2 SV=1 - [CLIP1_CHICK]	81.45	0.997	160.9	5.34
F1NKR8	Major vault protein OS=Gallus gallus GN=MVP PE=4 SV=2 - [F1NKR8_CHICK]	239.28	0.996	93.7	5.45
R4GMJ4	Eukaryotic translation initiation factor 3 subunit K OS=Gallus gallus GN=EIF3K PE=3 SV=1 - [R4GMJ4_CHICK]	67.16	0.996	34.2	6.24
Q5ZI72	Heterogeneous nuclear ribonucleoprotein D-like OS=Gallus gallus GN=HNRNPDL PE=2 SV=1 - [HNRDL_CHICK]	215.00	0.996	33.4	7.31
E1C3B2	Cytochrome c oxidase subunit 6A, mitochondrial OS=Gallus gallus GN=COX6A1 PE=3 SV=1 - [E1C3B2_CHICK]	0.00	0.996	11.8	9.35
Q5ZMN0	Acidic leucine-rich nuclear phosphoprotein 32 family member B OS=Gallus gallus GN=ANP32B PE=2 SV=1 - [AN32B_CHICK]	39.82	0.996	29.9	3.98
F1NZF0	DNA topoisomerase 2 OS=Gallus gallus GN=TOP2A PE=3 SV=2 - [F1NZF0_CHICK]	88.13	0.996	167.9	8.75
F1NTQ2	Beta-hexosaminidase (Fragment) OS=Gallus gallus GN=HEXB PE=3 SV=2 - [F1NTQ2_CHICK]	105.44	0.996	63.4	6.49
F1N9Y0	Tubulin gamma chain (Fragment) OS=Gallus gallus GN=TUBG1 PE=3 SV=2 - [F1N9Y0_CHICK]	22.35	0.995	48.3	5.90

F1NZ90	tRNA pseudouridine synthase (Fragment) OS=Gallus gallus GN=PUS1 PE=3 SV=2 - [F1NZ90_CHICK]	84.20	0.995	45.2	8.25
F2Z4K4	60S ribosomal protein L5 (Fragment) OS=Gallus gallus GN=RPL5 PE=3 SV=1 - [F2Z4K4_CHICK]	207.23	0.995	33.9	9.72
Q9PTR5	Lissencephaly-1 homolog OS=Gallus gallus GN=PAFAH1B1 PE=2 SV=3 - [LIS1_CHICK]	81.08	0.995	46.6	7.37
F1NJ60	Protein Hook homolog 1 OS=Gallus gallus GN=HOOK1 PE=4 SV=1 - [F1NJ60_CHICK]	0.00	0.995	83.0	5.21
P20135	Glutathione S-transferase theta-1 OS=Gallus gallus GN=GSTT1 PE=1 SV=3 - [GSTT1_CHICK]	0.00	0.995	29.8	7.11
Q6JLB2	Proteasome subunit beta type OS=Gallus gallus GN=PSMB1 PE=3 SV=1 - [Q6JLB2_CHICK]	168.99	0.995	26.0	6.89
Q5ZII9	Tuftelin-interacting protein 11 OS=Gallus gallus GN=TFIP11 PE=2 SV=1 - [TFP11_CHICK]	35.91	0.995	95.5	6.02
F1P180	Aspartate aminotransferase OS=Gallus gallus GN=GOT2 PE=3 SV=2 - [F1P180_CHICK]	480.25	0.995	47.3	9.33
Q90972	E3 ubiquitin-protein ligase RNF13 OS=Gallus gallus GN=RNF13 PE=1 SV=1 - [RNF13_CHICK]	0.00	0.995	42.8	4.97
Q90WD0	Actin-related protein 3 OS=Gallus gallus GN=ACTR3 PE=2 SV=1 - [ARP3_CHICK]	97.13	0.994	47.4	5.88
Q5ZKM0	Dysbindin OS=Gallus gallus GN=DTNBP1 PE=2 SV=1 - [DTBP1_CHICK]	0.00	0.994	39.6	4.59
R4GHX2	Peptidyl-prolyl cis-trans isomerase (Fragment) OS=Gallus gallus GN=PPIA PE=3 SV=1 - [R4GHX2_CHICK]	421.60	0.994	15.9	8.79
F1NAP9	RNA-binding protein with multiple-splicing (Fragment) OS=Gallus gallus GN=RBPMS PE=4 SV=1 - [F1NAP9_CHICK]	0.00	0.994	21.5	9.50

P62303	Small nuclear ribonucleoprotein E OS=Gallus gallus GN=SNRPE PE=3 SV=1 - [RUXE_CHICK]	0.00	0.994	10.8	9.44
Q9DDU8	Transformer-2 beta OS=Gallus gallus GN=TRA2B PE=2 SV=1 - [Q9DDU8_CHICK]	63.68	0.994	33.8	11.25
Q90Z16	Optineurin OS=Gallus gallus GN=OPTN PE=1 SV=1 - [OPTN_CHICK]	32.28	0.993	64.0	5.00
F1NQG5	Ribosomal protein L15 OS=Gallus gallus GN=RPL15 PE=3 SV=2 - [F1NQG5_CHICK]	73.10	0.993	24.1	11.62
Q91955	Myotrophin OS=Gallus gallus GN=MTPN PE=3 SV=1 - [MTPN_CHICK]	51.37	0.992	12.9	5.29
Q90771	Cyclin-dependent kinase OS=Gallus gallus GN=cdk6 PE=4 SV=1 - [Q90771_CHICK]	66.87	0.992	36.8	5.99
E1BRL4	Synaptosomal-associated protein OS=Gallus gallus GN=SNAP23 PE=3 SV=1 - [E1BRL4_CHICK]	0.00	0.992	23.6	5.01
Q5ZIF1	Adipocyte plasma membrane-associated protein OS=Gallus gallus GN=APMAP PE=2 SV=1 - [APMAP_CHICK]	137.57	0.992	46.1	6.21
F1NC33	Heat shock cognate protein HSP 90-beta OS=Gallus gallus GN=HSP90AB1 PE=3 SV=1 - [F1NC33_CHICK]	653.04	0.992	83.4	5.03
Q5ZIC8	Importin-13 OS=Gallus gallus GN=IPO13 PE=2 SV=1 - [IPO13_CHICK]	0.00	0.992	107.7	5.48
F1P3X9	Transcriptional enhancer factor TEF-5 OS=Gallus gallus GN=TEAD3 PE=4 SV=2 - [F1P3X9_CHICK]	23.90	0.992	46.3	8.78
F1NWH1	RNA polymerase II-associated protein 3 OS=Gallus gallus GN=RPAP3 PE=4 SV=1 - [F1NWH1_CHICK]	31.52	0.991	75.9	5.73
F1NQG6	Signal transducing adapter molecule 2 OS=Gallus gallus GN=STAM2 PE=4 SV=1 - [F1NQG6_CHICK]	41.81	0.991	48.4	4.84
P23614	Brain acid soluble protein 1 homolog OS=Gallus gallus GN=BASP1 PE=2 SV=4 - [BASP1_CHICK]	53.12	0.991	25.4	4.68

F1NGN4	Bifunctional arginine demethylase and lysyl-hydroxylase JMJD6 OS=Gallus gallus GN=JMJD6 PE=4 SV=1 - [F1NGN4_CHICK]	25.16	0.991	47.8	8.73
F1NLL8	Protein polybromo-1 OS=Gallus gallus GN=PBRM1 PE=4 SV=1 - [F1NLL8_CHICK]	0.00	0.991	186.7	6.83
P28687	Signal peptidase complex subunit 3 OS=Gallus gallus GN=SPC22 PE=1 SV=1 - [SPCS3_CHICK]	34.86	0.991	20.2	8.65
F1NJV4	Protein-L-isoaspartate O-methyltransferase OS=Gallus gallus GN=PCMT1 PE=3 SV=1 - [F1NJV4_CHICK]	51.16	0.991	21.2	5.78
P40618	High mobility group protein B3 OS=Gallus gallus GN=HMGB3 PE=2 SV=3 - [HMGB3_CHICK]	219.55	0.991	23.0	8.12
F1P2G4	Adenylyl cyclase-associated protein OS=Gallus gallus GN=CAP1 PE=3 SV=2 - [F1P2G4_CHICK]	247.85	0.990	57.6	7.99
Q5ZJQ7	RNA polymerase II subunit A C-terminal domain phosphatase SSU72 OS=Gallus gallus GN=SSU72 PE=2 SV=1 - [SSU72_CHICK]	63.20	0.990	22.6	5.33
P50890	40S ribosomal protein SA OS=Gallus gallus GN=RPSA PE=3 SV=1 - [RSSA_CHICK]	266.36	0.990	33.0	4.87
R9PXQ9	Fatty acyl-CoA reductase 1 OS=Gallus gallus GN=FAR1 PE=4 SV=1 - [R9PXQ9_CHICK]	47.72	0.990	56.5	9.01
Q5ZHT7	Signal recognition particle 9 kDa protein OS=Gallus gallus GN=SRP9 PE=3 SV=1 - [Q5ZHT7_CHICK]	0.00	0.990	10.1	7.88
Q9DEA3	Proliferating cell nuclear antigen OS=Gallus gallus GN=PCNA PE=1 SV=1 - [PCNA_CHICK]	72.93	0.990	28.9	4.72
Q5ZKF5	S-phase kinase-associated protein 1 OS=Gallus gallus GN=SKP1 PE=2 SV=1 - [SKP1_CHICK]	162.90	0.989	18.6	4.54
F1NWB8	p21-activated protein kinase-interacting protein 1-like OS=Gallus gallus GN=PAK1IP1 PE=4 SV=2 -	0.00	0.989	34.5	9.45

	[F1NWB8_CHICK]				
Q5F433	PRA1 family protein 3 OS=Gallus gallus GN=ARL6IP5 PE=2 SV=1 - [PRAF3_CHICK]	51.93	0.989	21.6	9.50
Q8UVD9	Far upstream element-binding protein 2 OS=Gallus gallus GN=KHSRP PE=1 SV=1 - [FUBP2_CHICK]	350.94	0.989	80.6	6.90
Q5F418	26S proteasome non-ATPase regulatory subunit 1 OS=Gallus gallus GN=PSMD1 PE=2 SV=1 - [PSMD1_CHICK]	248.46	0.989	106.0	5.36
B6ZLK1	FACT complex subunit SSRP1 OS=Gallus gallus GN=SSRP1 PE=2 SV=1 - [B6ZLK1_CHICK]	68.97	0.989	80.0	6.87
F1NN92	Uridine-cytidine kinase (Fragment) OS=Gallus gallus GN=UCK1 PE=3 SV=2 - [F1NN92_CHICK]	0.00	0.989	31.3	8.25
F1NFT8	WD repeat-containing protein 61 OS=Gallus gallus GN=WDR61 PE=4 SV=1 - [F1NFT8_CHICK]	43.80	0.989	33.3	5.47
F1NBB0	2-methoxy-6-polyprenyl-1,4-benzoquinol methylase, mitochondrial OS=Gallus gallus GN=COQ5 PE=3 SV=1 - [F1NBB0_CHICK]	0.00	0.989	35.1	7.21
P0CB05	Centrosomal protein of 63 kDa OS=Gallus gallus GN=CEP63 PE=2 SV=1 - [CEP63_CHICK]	0.00	0.989	82.0	5.36
Q9I8D6	T-complex protein 1 subunit delta OS=Gallus gallus GN=tcp-1 delta PE=2 SV=1 - [Q9I8D6_CHICK]	402.74	0.989	57.7	7.80
Q5ZHP5	Charged multivesicular body protein 4b OS=Gallus gallus GN=CHMP4B PE=2 SV=1 - [CHM4B_CHICK]	278.69	0.989	25.1	4.79
Q5ZMV3	Protein Dr1 OS=Gallus gallus GN=DR1 PE=2 SV=1 - [NC2B_CHICK]	57.37	0.989	19.4	4.75
F1NQT0	DNA polymerase OS=Gallus gallus GN=REV3L PE=3 SV=2 - [F1NQT0_CHICK]	16.84	0.989	344.3	8.07

Q5ZM91	cAMP-dependent protein kinase type I-alpha regulatory subunit OS=Gallus gallus GN=PRKAR1A PE=2 SV=1 - [KAP0_CHICK]	68.56	0.988	43.3	5.35
P33879	Sodium/potassium-transporting ATPase subunit beta-3 OS=Gallus gallus GN=ATP1B3 PE=2 SV=1 - [AT1B3_CHICK]	67.53	0.988	31.8	7.94
Q5ZLR5	Cytochrome b-c1 complex subunit Rieske, mitochondrial OS=Gallus gallus GN=UQCRFS1 PE=1 SV=1 - [UCRI_CHICK]	28.33	0.988	29.4	8.43
Q6ITC7	40S ribosomal protein S13 OS=Gallus gallus GN=RPS13 PE=2 SV=3 - [RS13_CHICK]	102.78	0.987	17.2	10.54
F1NI02	Argininosuccinate lyase (Fragment) OS=Gallus gallus GN=ASL2 PE=3 SV=1 - [F1NI02_CHICK]	35.10	0.987	50.9	6.40
Q90687	Tyrosine-protein phosphatase non-receptor type 11 OS=Gallus gallus GN=PTPN11 PE=2 SV=1 - [PTN11_CHICK]	57.07	0.987	67.9	7.20
F1P2L5	Large subunit GTPase 1 homolog OS=Gallus gallus GN=LSG1 PE=4 SV=1 - [F1P2L5_CHICK]	37.27	0.987	73.8	6.01
E1BRE2	NAD-dependent protein deacylase sirtuin-5, mitochondrial OS=Gallus gallus GN=SIRT5 PE=3 SV=1 - [SIR5_CHICK]	63.92	0.987	33.9	7.44
H9L0C4	Pre-mRNA-processing factor 19 (Fragment) OS=Gallus gallus GN=PRPF19 PE=4 SV=2 - [H9L0C4_CHICK]	96.81	0.987	40.5	6.74
F1P3L3	Semaphorin-3C OS=Gallus gallus GN=SEMA3C PE=4 SV=2 - [F1P3L3_CHICK]	48.61	0.987	85.4	8.68
R4GIW5	Phosphatidylinositol 5-phosphate 4-kinase type-2 alpha OS=Gallus gallus GN=PIP4K2A PE=4 SV=1 -	33.29	0.986	38.4	6.21

	[R4GIW5_CHICK]				
Q5ZJQ2	Phenylalanine--tRNA ligase alpha subunit OS=Gallus gallus GN=FARSA PE=1 SV=1 - [SYFA_CHICK]	115.79	0.986	49.5	8.92
F1NV93	Peptidyl-prolyl cis-trans isomerase (Fragment) OS=Gallus gallus GN=PPIB PE=3 SV=2 - [F1NV93_CHICK]	215.30	0.986	16.2	9.11
E1BR22	Calpain-2 catalytic subunit OS=Gallus gallus GN=CAPN2 PE=4 SV=1 - [E1BR22_CHICK]	165.13	0.986	79.2	5.58
E1BSF5	Branched-chain-amino-acid aminotransferase OS=Gallus gallus GN=BCAT1 PE=3 SV=2 - [E1BSF5_CHICK]	0.00	0.986	43.0	5.77
Q5SDR3	Polycomb complex protein BMI-1 OS=Gallus gallus GN=BMI1 PE=2 SV=1 - [BMI1_CHICK]	0.00	0.986	36.9	8.63
F1NVD4	Adenylosuccinate synthetase (Fragment) OS=Gallus gallus GN=ADSSL1 PE=3 SV=2 - [F1NVD4_CHICK]	153.88	0.985	48.8	7.96
Q5ZJ25	Vacuolar protein sorting-associated protein 51 homolog OS=Gallus gallus GN=VPS51 PE=2 SV=1 - [VPS51_CHICK]	28.03	0.985	86.0	6.42
F1NTD6	Activating signal cointegrator 1 complex subunit 3 OS=Gallus gallus GN=ascc3 PE=3 SV=2 - [ASCC3_CHICK]	0.00	0.985	251.7	6.77
P21868	Casein kinase II subunit alpha OS=Gallus gallus GN=CSNK2A1 PE=2 SV=1 - [CSK21_CHICK]	35.21	0.984	45.2	7.74
F1NJS6	Importin subunit alpha OS=Gallus gallus GN=KPNA2 PE=3 SV=1 - [F1NJS6_CHICK]	51.10	0.984	58.0	5.48
F1NL85	Magnesium transporter protein 1 OS=Gallus gallus GN=MAGT1 PE=4 SV=1 - [F1NL85_CHICK]	38.47	0.984	36.8	9.63

Q5ZIJ0	BUD13 homolog OS=Gallus gallus GN=BUD13 PE=2 SV=1 - [BUD13_CHICK]	0.00	0.984	64.4	9.89
Q8UUU0	Erg isoform C-1-1 OS=Gallus gallus GN=ERG PE=2 SV=1 - [Q8UUU0_CHICK]	39.00	0.984	51.0	7.62
Q5ZIT5	Ras-related protein Rab-10 OS=Gallus gallus GN=RAB10 PE=2 SV=1 - [RAB10_CHICK]	167.71	0.984	22.5	8.38
E1C4C0	Acyl carrier protein OS=Gallus gallus GN=NDUFAB1 PE=3 SV=2 - [E1C4C0_CHICK]	55.54	0.984	16.5	5.22
Q9YGW6	Ezrin OS=Gallus gallus GN=EZR PE=2 SV=1 - [Q9YGW6_CHICK]	396.69	0.984	69.3	6.20
Q5ZMU0	28S ribosomal protein S7, mitochondrial OS=Gallus gallus GN=MRPS7 PE=2 SV=2 - [RT07_CHICK]	0.00	0.983	27.3	9.72
F1NCA4	RNA binding protein fox-1 homolog OS=Gallus gallus GN=RBFOX2 PE=3 SV=2 - [F1NCA4_CHICK]	0.00	0.983	40.0	8.56
Q2PUH1	5'-AMP-activated protein kinase alpha-1 catalytic subunit OS=Gallus gallus GN=PRKAA1 PE=2 SV=1 - [Q2PUH1_CHICK]	38.94	0.983	64.1	7.97
Q5ZKJ4	FGFR1 oncogene partner 2 homolog OS=Gallus gallus GN=FGFR1OP2 PE=2 SV=1 - [FGOP2_CHICK]	0.00	0.983	25.2	6.33
Q5ZJ60	3-hydroxyisobutyryl-CoA hydrolase, mitochondrial OS=Gallus gallus GN=HIBCH PE=2 SV=1 - [HIBCH_CHICK]	167.60	0.983	42.8	8.44
P08629	Thioredoxin OS=Gallus gallus GN=TXN PE=3 SV=2 - [THIO_CHICK]	363.80	0.983	11.7	5.25
E1BR00	Adenylosuccinate lyase OS=Gallus gallus GN=ADSL PE=4 SV=2 - [E1BR00_CHICK]	65.39	0.983	54.6	7.12
F1NX30	Cell cycle control protein 50A OS=Gallus gallus GN=TMEM30A PE=4 SV=1 - [F1NX30_CHICK]	0.00	0.983	41.4	8.31

R4GKE0	Peptidyl-prolyl cis-trans isomerase (Fragment) OS=Gallus gallus GN=FKBP1A PE=4 SV=1 - [R4GKE0_CHICK]	240.99	0.983	8.9	7.25
F1P1H7	Rab GTPase-activating protein 1-like OS=Gallus gallus GN=RABGAP1L PE=4 SV=2 - [F1P1H7_CHICK]	0.00	0.983	92.5	5.29
R4GLB3	Proteasome subunit beta type OS=Gallus gallus GN=PSMB2 PE=3 SV=1 - [R4GLB3_CHICK]	34.25	0.982	22.7	6.54
P00789	Calpain-1 catalytic subunit OS=Gallus gallus PE=1 SV=2 - [CANX_CHICK]	77.57	0.982	80.3	5.00
Q5F3A4	RELT-like protein 1 OS=Gallus gallus GN=RELL1 PE=2 SV=1 - [RELL1_CHICK]	29.65	0.981	31.1	7.37
Q5ZLX5	Zinc finger Ran-binding domain-containing protein 2 OS=Gallus gallus GN=ZRANB2 PE=2 SV=1 - [ZRAB2_CHICK]	0.00	0.981	37.8	10.02
F1P1D9	Cysteine--tRNA ligase, cytoplasmic (Fragment) OS=Gallus gallus GN=CARS PE=3 SV=2 - [F1P1D9_CHICK]	40.09	0.981	85.0	6.70
Q5F3G7	Glycoprotein-N-acetylgalactosamine 3-beta-galactosyltransferase 1 OS=Gallus gallus GN=C1GALT1 PE=2 SV=1 - [C1GLT_CHICK]	0.00	0.981	42.6	6.35
F1NLL2	Beta-galactosidase OS=Gallus gallus GN=GLB1L3 PE=3 SV=2 - [F1NLL2_CHICK]	41.25	0.981	72.9	7.39
Q91000	Trans Golgi network protease furin OS=Gallus gallus GN=FURIN PE=2 SV=1 - [Q91000_CHICK]	63.11	0.980	86.6	6.43
O12940	Target of Myb protein 1 OS=Gallus gallus GN=TOM1 PE=2 SV=2 - [TOM1_CHICK]	65.86	0.980	57.0	5.19
Q5ZMD1	14-3-3 protein theta OS=Gallus gallus GN=YWHAQ PE=1 SV=1 - [1433T_CHICK]	585.27	0.980	27.8	4.78

F1NNI7	39S ribosomal protein L15, mitochondrial OS=Gallus gallus GN=MRPL15 PE=3 SV=1 - [F1NNI7_CHICK]	52.30	0.980	33.6	10.07
E1BTJ1	Glycylpeptide N-tetradecanoyltransferase OS=Gallus gallus GN=NMT2 PE=3 SV=1 - [E1BTJ1_CHICK]	0.00	0.980	56.8	7.53
Q04861	Nuclear factor NF-kappa-B p105 subunit OS=Gallus gallus GN=NFKB1 PE=2 SV=2 - [NFKB1_CHICK]	42.15	0.980	107.9	5.77
F1NZM1	Eukaryotic translation initiation factor 3 subunit E OS=Gallus gallus GN=EIF3E PE=3 SV=1 - [F1NZM1_CHICK]	175.56	0.979	52.1	6.04
F1NEQ6	Proteasome subunit alpha type (Fragment) OS=Gallus gallus GN=PSMA6 PE=3 SV=1 - [F1NEQ6_CHICK]	151.95	0.979	27.7	6.55
E1C810	Ribosomal protein L37 OS=Gallus gallus GN=RPL37 PE=3 SV=1 - [E1C810_CHICK]	39.79	0.978	11.1	11.74
P09653	Tubulin beta-5 chain OS=Gallus gallus PE=3 SV=1 - [TBB5_CHICK]	743.19	0.978	49.9	4.88
Q8UWG7	60S ribosomal protein L6 OS=Gallus gallus GN=RPL6 PE=2 SV=1 - [Q8UWG7_CHICK]	89.85	0.978	33.9	10.67
E1BRK2	Histone deacetylase complex subunit SAP130 OS=Gallus gallus GN=SAP130 PE=4 SV=2 - [E1BRK2_CHICK]	0.00	0.978	110.8	9.91
F1NCB2	Sodium/hydrogen exchanger OS=Gallus gallus GN=SLC9A6 PE=3 SV=2 - [F1NCB2_CHICK]	44.23	0.978	67.9	5.86
Q5ZHQ1	Small ubiquitin-related modifier 3 OS=Gallus gallus GN=SUMO3 PE=3 SV=1 - [SUMO3_CHICK]	107.05	0.977	10.7	5.50
F1NK38	T-complex protein 1 subunit eta OS=Gallus gallus GN=CCT7 PE=3 SV=2 - [F1NK38_CHICK]	532.63	0.977	60.0	6.09
F1NS23	Adenylosuccinate synthetase (Fragment) OS=Gallus gallus GN=ADSS PE=3 SV=1 - [F1NS23_CHICK]	0.00	0.977	43.9	6.38

E1C718	Ubiquitin carboxyl-terminal hydrolase OS=Gallus gallus GN=USP15 PE=3 SV=2 - [E1C718_CHICK]	63.75	0.977	112.0	5.26
F1NG91	Methyltransferase-like protein 9 (Fragment) OS=Gallus gallus GN=METTL9 PE=4 SV=2 - [F1NG91_CHICK]	0.00	0.977	35.8	6.43
F1NFW2	Hydroxypyruvate isomerase (Fragment) OS=Gallus gallus PE=3 SV=2 - [F1NFW2_CHICK]	0.00	0.977	29.4	7.44
P21869	Casein kinase II subunit alpha' OS=Gallus gallus PE=2 SV=1 - [CSK22_CHICK]	62.27	0.977	41.2	8.25
Q5ZKG5	Low molecular weight phosphotyrosine protein phosphatase OS=Gallus gallus GN=ACP1 PE=2 SV=3 - [PPAC_CHICK]	101.98	0.977	18.2	7.20
F1NXK3	Palmitoyltransferase OS=Gallus gallus GN=ZDHHC5 PE=3 SV=1 - [F1NXK3_CHICK]	20.20	0.976	78.2	9.11
F1NIG8	Cholecystokinin OS=Gallus gallus GN=CCK PE=3 SV=1 - [F1NIG8_CHICK]	0.00	0.976	14.1	8.03
Q90932	Nuclear factor 1 X-type OS=Gallus gallus GN=NFIX PE=2 SV=1 - [NFIX_CHICK]	34.19	0.976	46.9	8.29
Q5ZJU4	Ubiquitin-related modifier 1 OS=Gallus gallus GN=URM1 PE=3 SV=1 - [URM1_CHICK]	0.00	0.976	11.4	4.84
F1P204	Multidrug resistance-associated protein 1 OS=Gallus gallus GN=ABCC1 PE=3 SV=2 - [F1P204_CHICK]	94.88	0.976	170.8	7.88
R4GHW9	Erythroid protein 4.1 OS=Gallus gallus GN=EPB41 PE=4 SV=1 - [R4GHW9_CHICK]	53.63	0.976	96.8	5.92
Q5ZM60	Cell cycle progression protein 1 OS=Gallus gallus GN=CCPG1 PE=2 SV=1 - [CCPG1_CHICK]	38.61	0.976	93.4	6.32
Q5ZIW2	CCR4-NOT transcription complex subunit 10 OS=Gallus gallus GN=CNOT10 PE=2 SV=1 -	0.00	0.975	82.1	7.66

	[CNO10_CHICK]				
Q5ZHT1	Acyl-CoA dehydrogenase family member 11 OS=Gallus gallus GN=ACAD11 PE=2 SV=1 - [ACD11_CHICK]	0.00	0.975	86.9	8.15
Q5ZJ54	T-complex protein 1 subunit zeta OS=Gallus gallus GN=CCT6 PE=1 SV=3 - [TCPZ_CHICK]	332.94	0.975	57.6	6.81
F1NW23	Clathrin heavy chain (Fragment) OS=Gallus gallus GN=CLTC PE=3 SV=1 - [F1NW23_CHICK]	959.29	0.975	189.9	5.72
Q04205	Tensin OS=Gallus gallus GN=TNS PE=1 SV=2 - [TENS_CHICK]	84.63	0.975	187.1	7.34
Q9DGN6	Endothelin converting enzyme-1 OS=Gallus gallus GN=ECE-1 PE=2 SV=1 - [Q9DGN6_CHICK]	38.74	0.975	84.9	5.22
Q5ZML6	Protein FAM210A OS=Gallus gallus GN=FAM210A PE=2 SV=1 - [F210A_CHICK]	0.00	0.974	30.4	9.29
F1NB52	Coatomer subunit gamma OS=Gallus gallus GN=COPG PE=3 SV=1 - [F1NB52_CHICK]	236.62	0.974	97.5	5.45
Q3C2H6	Protein Wnt OS=Gallus gallus GN=Wnt6 PE=2 SV=1 - [Q3C2H6_CHICK]	0.00	0.974	39.3	8.38
O42254	Insulin-like growth factor 2 mRNA-binding protein 1 OS=Gallus gallus GN=IGF2BP1 PE=1 SV=1 - [IF2B1_CHICK]	358.11	0.974	63.2	9.14
Q9PTD5	Mitochondrial fission regulator 1 OS=Gallus gallus GN=MTFR1 PE=1 SV=2 - [MTFR1_CHICK]	0.00	0.974	37.0	9.36
P00940	Triosephosphate isomerase OS=Gallus gallus GN=TPI1 PE=1 SV=2 - [TPIS_CHICK]	245.17	0.974	26.6	7.20
F1NDG9	Mesoderm induction early response protein 1 (Fragment) OS=Gallus gallus GN=MIER1 PE=4 SV=1 - [F1NDG9_CHICK]	0.00	0.974	57.7	4.39

Q5F3T9	UDP-glucose 6-dehydrogenase OS=Gallus gallus GN=UGDH PE=2 SV=1 - [UGDH_CHICK]	71.65	0.974	55.0	7.27
F1P3Y7	Serine/threonine-protein kinase 11-interacting protein (Fragment) OS=Gallus gallus GN=STK11IP PE=4 SV=2 - [F1P3Y7_CHICK]	0.00	0.974	118.9	5.54
F1NV68	Importin subunit alpha (Fragment) OS=Gallus gallus GN=KPNA3 PE=3 SV=2 - [F1NV68_CHICK]	50.26	0.973	55.3	4.81
F1NKN6	Lens epithelium-derived growth factor OS=Gallus gallus GN=PSIP1 PE=4 SV=2 - [F1NKN6_CHICK]	0.00	0.973	62.6	9.31
P38024	Multifunctional protein ADE2 OS=Gallus gallus GN=AIRC PE=2 SV=1 - [PUR6_CHICK]	152.50	0.973	47.2	7.93
F1NQB6	Unconventional myosin-Va OS=Gallus gallus GN=MYO5A PE=4 SV=2 - [F1NQB6_CHICK]	145.64	0.973	212.3	8.47
F1NIQ3	Sister chromatid cohesion protein PDS5 homolog A OS=Gallus gallus GN=PDS5A PE=4 SV=1 - [F1NIQ3_CHICK]	70.11	0.973	150.0	7.83
F1P2D7	Chromatin assembly factor 1 subunit B OS=Gallus gallus GN=CHAF1B PE=4 SV=1 - [F1P2D7_CHICK]	17.21	0.973	62.6	7.66
Q5F3D7	U3 small nucleolar RNA-associated protein 15 homolog OS=Gallus gallus GN=UTP15 PE=2 SV=1 - [UTP15_CHICK]	0.00	0.972	58.4	9.04
F1NE97	Nuclear migration protein nudC OS=Gallus gallus GN=NUDC PE=4 SV=1 - [F1NE97_CHICK]	115.95	0.972	39.2	5.40
E1BZL0	Ubiquitin carboxyl-terminal hydrolase OS=Gallus gallus GN=USP24 PE=3 SV=2 - [E1BZL0_CHICK]	0.00	0.972	258.7	6.58
F1NKK7	Transmembrane protein 175 OS=Gallus gallus GN=TMEM175 PE=4 SV=1 - [F1NKK7_CHICK]	17.03	0.972	56.1	6.61

E1C650	P2X purinoceptor OS=Gallus gallus GN=P2RX4 PE=3 SV=1 - [E1C650_CHICK]	32.41	0.972	43.3	7.99
Q5ZM36	Eukaryotic initiation factor 4A-III OS=Gallus gallus GN=EIF4A3 PE=2 SV=1 - [IF4A3_CHICK]	204.72	0.972	46.8	6.73
P21566	Cofilin-2 OS=Gallus gallus GN=CFL2 PE=1 SV=2 - [COF2_CHICK]	528.01	0.972	18.6	7.88
P48440	Dolichyl-diphosphooligosaccharide--protein glycosyltransferase 48 kDa subunit OS=Gallus gallus GN=DDOST PE=1 SV=1 - [OST48_CHICK]	103.53	0.972	45.9	5.58
F1N9T0	Hydroxymethylglutaryl-CoA synthase, cytoplasmic OS=Gallus gallus GN=HMGCS1 PE=4 SV=1 - [F1N9T0_CHICK]	60.02	0.972	57.6	5.74
P50594	Protein mago nashi homolog OS=Gallus gallus GN=MAGOH PE=2 SV=2 - [MGN_CHICK]	43.15	0.972	17.1	6.39
R4GM98	Nucleoside diphosphate kinase (Fragment) OS=Gallus gallus GN=NME3 PE=3 SV=1 - [R4GM98_CHICK]	111.95	0.971	21.0	7.84
Q5ZMG9	T-complex protein 1 subunit alpha OS=Gallus gallus GN=TCP1 PE=2 SV=1 - [Q5ZMG9_CHICK]	309.02	0.971	60.4	5.88
P14315-2	Isoform 2 of F-actin-capping protein subunit beta isoforms 1 and 2 OS=Gallus gallus GN=CAPZB - [CAPZB_CHICK]	94.16	0.971	30.6	6.01
E1C050	Eukaryotic translation initiation factor 3 subunit F OS=Gallus gallus GN=EIF3F PE=3 SV=1 - [E1C050_CHICK]	171.45	0.971	35.2	5.58
R4GK07	Probable cytosolic iron-sulfur protein assembly protein CIAO1 OS=Gallus gallus GN=CIAO1 PE=3 SV=1 - [R4GK07_CHICK]	0.00	0.971	40.5	5.81

Q5ZIR8	Mitochondrial import inner membrane translocase subunit Tim9 OS=Gallus gallus GN=TIMM9 PE=3 SV=1 - [TIM9_CHICK]	0.00	0.971	10.5	7.21
Q6EE31	T-complex protein 1 subunit theta OS=Gallus gallus GN=CCT8 PE=1 SV=3 - [TCPQ_CHICK]	756.17	0.971	59.4	5.53
Q5ZM32	Dihydrolipoyl dehydrogenase OS=Gallus gallus GN=DLD PE=2 SV=1 - [Q5ZM32_CHICK]	191.41	0.971	53.9	7.99
F1NRI3	WD repeat-containing protein 1 OS=Gallus gallus GN=WDR1 PE=4 SV=2 - [F1NRI3_CHICK]	219.85	0.970	66.5	6.67
F1NWL8	LisH domain-containing protein FOPNL OS=Gallus gallus GN=FOPNL PE=4 SV=1 - [F1NWL8_CHICK]	0.00	0.970	19.7	8.10
P14093	ATP synthase protein 8 OS=Gallus gallus GN=MT-ATP8 PE=3 SV=1 - [ATP8_CHICK]	70.68	0.970	6.4	10.30
Q9PSX7	Rho-related GTP-binding protein RhoC OS=Gallus gallus GN=RHOC PE=1 SV=1 - [RHOC_CHICK]	0.00	0.970	22.0	6.58
Q1T766	Nsl1 protein OS=Gallus gallus GN=Nsl1/DC31 PE=2 SV=1 - [Q1T766_CHICK]	0.00	0.970	32.1	8.22
R9PXN3	Testin OS=Gallus gallus GN=TES PE=4 SV=1 - [R9PXN3_CHICK]	148.19	0.970	47.1	7.49
F1P376	Protein wntless homolog OS=Gallus gallus GN=WLS PE=4 SV=1 - [F1P376_CHICK]	49.74	0.970	62.1	6.96
F1NRQ9	Transforming growth factor beta-3 (Fragment) OS=Gallus gallus GN=TGFB3 PE=3 SV=2 - [F1NRQ9_CHICK]	0.00	0.970	33.8	7.80
Q5ZLI2	Proteasome subunit alpha type OS=Gallus gallus GN=PSMA3 PE=2 SV=1 - [Q5ZLI2_CHICK]	189.73	0.969	28.5	5.05
P18937	NADH-ubiquinone oxidoreductase chain 2 OS=Gallus gallus GN=MT-ND2 PE=3 SV=1 - [NU2M_CHICK]	0.00	0.969	38.3	9.91

Q90700	Calcium modulating cyclophilin ligand CAML OS=Gallus gallus GN=CAMLG PE=2 SV=1 - [Q90700_CHICK]	0.00	0.969	31.9	8.15
Q5ZHN1	Charged multivesicular body protein 2a OS=Gallus gallus GN=CHMP2A PE=2 SV=1 - [CHM2A_CHICK]	23.48	0.969	24.7	5.67
F1NH92	Apolipoprotein O-like OS=Gallus gallus GN=APOOL PE=4 SV=1 - [F1NH92_CHICK]	66.28	0.969	30.1	9.55
Q5ZIP3	Deoxyhypusine hydroxylase OS=Gallus gallus GN=DOHH PE=2 SV=1 - [DOHH_CHICK]	46.58	0.968	32.9	4.83
Q5ZID0	NmrA-like family domain-containing protein 1 OS=Gallus gallus GN=NMRAL1 PE=2 SV=1 - [NMRL1_CHICK]	232.54	0.968	32.7	8.05
Q5ZKU5	Ras-related protein Rab-14 OS=Gallus gallus GN=RAB14 PE=2 SV=3 - [RAB14_CHICK]	56.65	0.968	23.9	6.21
R4GKT2	Guanine nucleotide-binding protein subunit gamma OS=Gallus gallus GN=LOC100858976 PE=3 SV=1 - [R4GKT2_CHICK]	74.21	0.968	7.6	8.51
Q71R46	Midline 1 OS=Gallus gallus GN=MID1 PE=2 SV=1 - [Q71R46_CHICK]	29.77	0.968	75.4	6.76
Q98953	Protein S100-A6 OS=Gallus gallus GN=S100A6 PE=3 SV=1 - [S10A6_CHICK]	172.45	0.968	10.3	5.01
F1N9A6	E3 ubiquitin-protein ligase BRE1A OS=Gallus gallus GN=RNF20 PE=4 SV=1 - [F1N9A6_CHICK]	75.33	0.968	114.7	5.92
A5HUM1	Transporter associated with antigen presentation 2 OS=Gallus gallus GN=TAP2 PE=2 SV=1 - [A5HUM1_CHICK]	0.00	0.968	75.9	7.30
P23668	16 kDa beta-galactoside-binding lectin OS=Gallus gallus PE=1 SV=1 - [LEG6_CHICK]	51.98	0.968	14.9	5.07

F1NMA3	Sulfotransferase family cytosolic 1B member 1 OS=Gallus gallus GN=SULT1B1 PE=4 SV=2 - [F1NMA3_CHICK]	0.00	0.967	34.0	6.67
F1P0I3	Probable ATP-dependent RNA helicase DDX10 OS=Gallus gallus GN=DDX10 PE=4 SV=1 - [F1P0I3_CHICK]	0.00	0.967	100.1	8.29
F1NDR9	Putative GTP cyclohydrolase 1 type 2 OS=Gallus gallus GN=NIF3L1 PE=3 SV=2 - [F1NDR9_CHICK]	83.44	0.967	41.1	6.98
P34065	Proteasome subunit beta type-5 (Fragment) OS=Gallus gallus GN=PSMB5 PE=2 SV=2 - [PSB5_CHICK]	219.04	0.967	27.0	8.68
O57389	CNP2 OS=Gallus gallus GN=CNP PE=2 SV=1 - [O57389_CHICK]	310.03	0.966	47.2	9.07
Q5ZIJ2	28S ribosomal protein S6, mitochondrial OS=Gallus gallus GN=MRPS6 PE=2 SV=3 - [RT06_CHICK]	34.14	0.966	13.8	9.61
Q5ZKV9	Coiled-coil domain-containing protein 132 OS=Gallus gallus GN=CCDC132 PE=2 SV=1 - [CC132_CHICK]	0.00	0.966	109.5	5.77
Q5ZHS3	Nuclear nucleic acid-binding protein C1D OS=Gallus gallus GN=C1D PE=2 SV=1 - [C1D_CHICK]	0.00	0.966	16.4	8.75
P26990	ADP-ribosylation factor 6 OS=Gallus gallus GN=ARF6 PE=2 SV=3 - [ARF6_CHICK]	146.98	0.966	20.1	8.95
Q02391	Golgi apparatus protein 1 OS=Gallus gallus GN=GLG1 PE=1 SV=1 - [GSLG1_CHICK]	127.56	0.965	129.6	6.86
F1NA27	Receptor-type tyrosine-protein phosphatase gamma OS=Gallus gallus GN=PTPRG PE=4 SV=2 - [F1NA27_CHICK]	0.00	0.965	140.0	5.48
F1NBV0	Peroxiredoxin-6 OS=Gallus gallus GN=PRDX6 PE=4 SV=1 - [F1NBV0_CHICK]	513.34	0.965	25.1	6.38

F1NRM5	Actin-related protein 2 (Fragment) OS=Gallus gallus GN=ACTR2 PE=3 SV=1 - [F1NRM5_CHICK]	68.12	0.965	43.0	6.74
H9L3L1	Condensin complex subunit 2 OS=Gallus gallus GN=777261 PE=3 SV=2 - [H9L3L1_CHICK]	35.38	0.965	78.4	5.48
P31335	Bifunctional purine biosynthesis protein PURH OS=Gallus gallus GN=ATIC PE=1 SV=1 - [PUR9_CHICK]	255.69	0.965	64.4	8.18
A3R0S3	Growth factor receptor-bound protein 2 OS=Gallus gallus GN=GRB2 PE=2 SV=1 - [A3R0S3_CHICK]	39.26	0.965	25.2	6.15
Q8JGM8	BH3-interacting domain death agonist OS=Gallus gallus GN=BID PE=2 SV=2 - [BID_CHICK]	110.92	0.964	21.7	4.87
F1NJI0	Annexin (Fragment) OS=Gallus gallus GN=ANXA5 PE=3 SV=1 - [F1NJI0_CHICK]	391.43	0.964	36.2	5.82
F1N8H6	Heterochromatin protein 1-binding protein 3 OS=Gallus gallus GN=HP1BP3 PE=3 SV=1 - [F1N8H6_CHICK]	144.82	0.964	61.8	9.33
E1C0G5	Ubiquitin carboxyl-terminal hydrolase OS=Gallus gallus GN=USP8 PE=3 SV=2 - [E1C0G5_CHICK]	0.00	0.964	127.9	8.34
F1NFR4	Exportin-7 OS=Gallus gallus GN=XPO7 PE=4 SV=2 - [F1NFR4_CHICK]	40.84	0.963	123.8	6.35
F1NMJ9	Tyrosine-protein kinase OS=Gallus gallus GN=JAK1 PE=3 SV=2 - [F1NMJ9_CHICK]	51.75	0.962	132.9	7.47
F1NJM1	tRNA (cytosine(34)-C(5))-methyltransferase OS=Gallus gallus GN=NSUN2 PE=4 SV=1 - [F1NJM1_CHICK]	39.36	0.962	90.5	6.77
H9L023	Nicalin OS=Gallus gallus GN=NCLN PE=4 SV=2 - [H9L023_CHICK]	68.30	0.962	63.0	6.35
E1C2S1	Talin-1 OS=Gallus gallus GN=TLN1 PE=4 SV=2 - [E1C2S1_CHICK]	1525.96	0.962	271.7	6.23

F1NPJ4	Succinate dehydrogenase [ubiquinone] flavoprotein subunit, mitochondrial OS=Gallus gallus GN=SDHA PE=4 SV=2 - [F1NPJ4_CHICK]	79.53	0.962	72.9	7.18
P41239	Tyrosine-protein kinase CSK OS=Gallus gallus GN=CSK PE=1 SV=1 - [CSK_CHICK]	88.21	0.962	50.7	7.42
F1NUA2	Actin-related protein 2/3 complex subunit 5 (Fragment) OS=Gallus gallus GN=ARPC5 PE=3 SV=2 - [F1NUA2_CHICK]	68.42	0.961	11.1	9.64
F1NBA0	Syntaxin-binding protein 1 (Fragment) OS=Gallus gallus GN=STXBP1 PE=4 SV=2 - [F1NBA0_CHICK]	20.38	0.961	67.4	6.60
E1C2P3	Heat shock 70 kDa protein 14 OS=Gallus gallus GN=HSPA14 PE=3 SV=1 - [HSP7E_CHICK]	88.26	0.960	54.1	5.30
F1NB68	Paxillin (Fragment) OS=Gallus gallus GN=PXN PE=4 SV=2 - [F1NB68_CHICK]	33.63	0.960	60.8	6.71
F1NSP4	BSD domain-containing protein 1 OS=Gallus gallus GN=BSDC1 PE=4 SV=1 - [F1NSP4_CHICK]	0.00	0.960	49.4	4.54
E1BTX8	Caldesmon OS=Gallus gallus GN=CAD PE=3 SV=2 - [E1BTX8_CHICK]	17.49	0.960	200.7	6.30
P67869	Casein kinase II subunit beta OS=Gallus gallus GN=CSNK2B PE=2 SV=1 - [CSK2B_CHICK]	62.81	0.960	24.9	5.55
Q805F9	DNA damage-binding protein 1 OS=Gallus gallus GN=DDB1 PE=2 SV=1 - [DDB1_CHICK]	119.01	0.960	126.9	5.26
F1NT76	Hyccin OS=Gallus gallus GN=FAM126A PE=4 SV=2 - [F1NT76_CHICK]	30.95	0.960	57.0	7.96
F1P157	Exportin-4 (Fragment) OS=Gallus gallus GN=XPO4 PE=4 SV=1 - [F1P157_CHICK]	31.77	0.960	129.7	5.14
F1N914	Pyrroline-5-carboxylate reductase OS=Gallus gallus GN=PYCR2 PE=3 SV=2 - [F1N914_CHICK]	229.11	0.960	33.2	8.02

F1NZP5	Calfacilitin OS=Gallus gallus GN=TLCD1 PE=2 SV=2 - [F1NZP5_CHICK]	0.00	0.959	28.3	9.74
Q5ZJ00	55 kDa erythrocyte membrane protein OS=Gallus gallus GN=MPP1 PE=2 SV=1 - [EM55_CHICK]	189.19	0.959	52.5	7.09
F1NFS3	Putative oxidoreductase GLYR1 (Fragment) OS=Gallus gallus GN=GLYR1 PE=4 SV=2 - [F1NFS3_CHICK]	41.69	0.958	60.4	9.25
F1NK29	Na(+)/H(+) exchange regulatory cofactor NHE-RF1 OS=Gallus gallus GN=SLC9A3R1 PE=4 SV=1 - [F1NK29_CHICK]	62.30	0.958	35.9	6.16
Q5ZMV7	WD repeat-containing protein 82 OS=Gallus gallus GN=WDR82 PE=2 SV=1 - [WDR82_CHICK]	0.00	0.958	35.1	7.69
F1P3J5	Lipase (Fragment) OS=Gallus gallus GN=LIPA PE=3 SV=2 - [F1P3J5_CHICK]	57.05	0.958	45.7	8.81
F1P3F1	Adenosylhomocysteinase OS=Gallus gallus GN=Gga.51956 PE=3 SV=2 - [F1P3F1_CHICK]	526.32	0.958	74.4	9.23
Q5ZLR6	Rho guanine nucleotide exchange factor 6 OS=Gallus gallus GN=ARHGEF6 PE=2 SV=1 - [ARHG6_CHICK]	0.00	0.958	85.9	6.10
Q800W4	TIA-1 OS=Gallus gallus GN=TIA1 PE=2 SV=1 - [Q800W4_CHICK]	53.78	0.957	41.3	7.72
F1NBD7	Cyclin-dependent kinase 1 OS=Gallus gallus GN=CDK1 PE=4 SV=1 - [F1NBD7_CHICK]	28.44	0.957	34.7	7.97
F1NNI2	Lon protease homolog (Fragment) OS=Gallus gallus GN=LONP1 PE=3 SV=2 - [F1NNI2_CHICK]	128.92	0.957	95.1	6.09
F1NSP7	26S protease regulatory subunit 4 (Fragment) OS=Gallus gallus GN=PSMC1 PE=4 SV=2 - [F1NSP7_CHICK]	260.05	0.957	47.5	6.11
Q5ZK92-2	Isoform 2 of Spastin OS=Gallus gallus GN=SPAST - [SPAST_CHICK]	0.00	0.957	62.6	9.70

Q7T048	Aryl-hydrocarbon receptor-interacting protein OS=Gallus gallus GN=AIP PE=2 SV=1 - [Q7T048_CHICK]	36.07	0.956	37.5	7.20
F1NDF3	NADH dehydrogenase [ubiquinone] 1 alpha subcomplex subunit 2 (Fragment) OS=Gallus gallus GN=NDUFA2 PE=3 SV=1 - [F1NDF3_CHICK]	39.08	0.956	11.2	9.91
O93327-2	Isoform 1 of Core histone macro-H2A.1 OS=Gallus gallus GN=H2AFY - [H2AY_CHICK]	133.04	0.956	39.2	9.83
O57476	Hsp90 co-chaperone Cdc37 OS=Gallus gallus GN=CDC37 PE=2 SV=1 - [CDC37_CHICK]	132.37	0.956	45.6	5.33
E1C129	Cytosolic Fe-S cluster assembly factor NUBP1 OS=Gallus gallus GN=NUBP1 PE=3 SV=1 - [E1C129_CHICK]	87.18	0.956	34.5	5.31
F1NF62	Coiled-coil domain-containing protein 93 OS=Gallus gallus GN=CCDC93 PE=4 SV=1 - [F1NF62_CHICK]	42.71	0.955	71.6	6.65
F1NPX4	Vacuolar fusion protein CCZ1 homolog OS=Gallus gallus GN=CCZ1 PE=4 SV=1 - [F1NPX4_CHICK]	0.00	0.955	55.1	6.30
Q9PTG6	Dynactin subunit 2 OS=Gallus gallus GN=DCTN2 PE=2 SV=1 - [DCTN2_CHICK]	272.68	0.955	45.1	4.98
Q5ZIA5	Coatomer subunit beta OS=Gallus gallus GN=COPB1 PE=2 SV=1 - [COPB_CHICK]	245.19	0.955	107.1	6.00
P48463	Serine/threonine-protein phosphatase 2A catalytic subunit alpha isoform OS=Gallus gallus GN=PPP2CA PE=2 SV=1 - [PP2AA_CHICK]	47.33	0.955	35.5	5.43
Q5ZKF6	7-methylguanosine phosphate-specific 5'-nucleotidase OS=Gallus gallus GN=NT5C3B PE=2 SV=1 - [5NT3B_CHICK]	0.00	0.955	33.1	5.55
Q05744	Cathepsin D OS=Gallus gallus GN=CTSD PE=1 SV=1 - [CATD_CHICK]	194.09	0.954	43.3	6.32

Q2KN97	Cytospin-A OS=Gallus gallus GN=SPECC1L PE=2 SV=2 - [CYTSA_CHICK]	38.53	0.954	124.8	5.71
E1BTE0	Single-stranded DNA-binding protein OS=Gallus gallus GN=SSBP1 PE=3 SV=2 - [E1BTE0_CHICK]	47.50	0.954	17.5	9.45
E1C6T8	Eukaryotic translation initiation factor 3 subunit I OS=Gallus gallus GN=EIF3I PE=3 SV=1 - [E1C6T8_CHICK]	76.76	0.954	36.5	5.64
F1NEJ8	Transmembrane protein 70, mitochondrial OS=Gallus gallus GN=TMEM70 PE=4 SV=1 - [F1NEJ8_CHICK]	45.60	0.954	27.4	9.31
P63247	Guanine nucleotide-binding protein subunit beta-2-like 1 OS=Gallus gallus GN=GNB2L1 PE=2 SV=1 - [GBLP_CHICK]	44.29	0.954	35.1	7.69
Q6DV79	Signal transducer and activator of transcription 3 OS=Gallus gallus GN=STAT3 PE=1 SV=2 - [STAT3_CHICK]	49.76	0.954	88.1	6.30
F1NAD9	Phosphorylase OS=Gallus gallus GN=PYGL PE=3 SV=2 - [F1NAD9_CHICK]	176.21	0.953	87.4	5.87
F1NLH9	Inosine triphosphate pyrophosphatase OS=Gallus gallus GN=ITPA PE=3 SV=2 - [ITPA_CHICK]	59.13	0.953	22.2	6.05
P10288	Cadherin-2 OS=Gallus gallus GN=CDH2 PE=1 SV=1 - [CADH2_CHICK]	73.73	0.953	100.4	4.78
H9L047	Acidic leucine-rich nuclear phosphoprotein 32 family member E OS=Gallus gallus GN=ANP32E PE=4 SV=2 - [H9L047_CHICK]	61.03	0.953	28.9	3.88
Q7T2U9	Transcription factor CP2 OS=Gallus gallus GN=TFCP2 PE=2 SV=1 - [TFCP2_CHICK]	18.41	0.953	56.4	5.80
Q5ZLQ6	14-3-3 protein beta/alpha OS=Gallus gallus GN=YWHAB PE=2 SV=1 - [1433B_CHICK]	626.23	0.953	27.9	4.83

F1P1L9	Serine/threonine-protein kinase B-raf (Fragment) OS=Gallus gallus GN=BRAF PE=4 SV=1 - [F1P1L9_CHICK]	0.00	0.953	84.9	8.47
P67883	60S ribosomal protein L30 OS=Gallus gallus GN=RPL30 PE=3 SV=2 - [RL30_CHICK]	0.00	0.953	12.8	9.63
F1NZC6	Phosphoribosyl pyrophosphate synthase-associated protein 2 OS=Gallus gallus GN=PRPSAP2 PE=4 SV=1 - [F1NZC6_CHICK]	99.24	0.952	40.7	7.17
F1NZW7	Peptidyl-prolyl cis-trans isomerase OS=Gallus gallus GN=PPIC PE=3 SV=2 - [F1NZW7_CHICK]	132.62	0.952	24.7	8.75
Q5ZIR1	Endophilin-B1 OS=Gallus gallus GN=SH3GLB1 PE=2 SV=1 - [SHLB1_CHICK]	118.53	0.952	40.8	6.29
F1P4G0	Methionine aminopeptidase 2 OS=Gallus gallus GN=METAP2 PE=3 SV=1 - [F1P4G0_CHICK]	0.00	0.952	53.1	5.81
Q09121	Eukaryotic translation initiation factor 5A-1 OS=Gallus gallus GN=EIF5A1 PE=1 SV=3 - [IF5A1_CHICK]	161.15	0.952	15.9	5.55
F1NPG2	Isocitrate dehydrogenase [NADP] (Fragment) OS=Gallus gallus GN=IDH1 PE=3 SV=1 - [F1NPG2_CHICK]	173.60	0.951	46.9	8.12
Q5ZK62	Arf-GAP with coiled-coil, ANK repeat and PH domain-containing protein 2 OS=Gallus gallus GN=ACAP2 PE=2 SV=1 - [ACAP2_CHICK]	43.62	0.951	88.4	6.77
Q5ZLL8	Glycerol-3-phosphate acyltransferase 3 OS=Gallus gallus GN=AGPAT9 PE=2 SV=1 - [GPAT3_CHICK]	0.00	0.951	50.1	8.94
Q45QT1	Soluble epoxide hydrolase OS=Gallus gallus GN=EPHX2 PE=2 SV=1 - [Q45QT1_CHICK]	97.05	0.951	63.2	6.29
E1C5T4	5-aminolevulinate synthase OS=Gallus gallus GN=ALAS1 PE=3 SV=2 - [E1C5T4_CHICK]	0.00	0.950	69.9	8.37

Q5ZKG8	Golgi to ER traffic protein 4 homolog OS=Gallus gallus GN=GET4 PE=2 SV=1 - [GET4_CHICK]	61.60	0.950	34.6	5.55
F1P3A5	Proto-oncogene c-Rel OS=Gallus gallus GN=REL PE=4 SV=2 - [F1P3A5_CHICK]	0.00	0.950	66.9	6.49
Q5ZJA4	Actin-related protein 5 OS=Gallus gallus GN=ACTR5 PE=2 SV=1 - [ARP5_CHICK]	0.00	0.950	69.3	5.34
F1NFS0	Elongation factor 2 (Fragment) OS=Gallus gallus GN=EEF2 PE=4 SV=2 - [F1NFS0_CHICK]	560.36	0.950	95.2	6.83
F1NMK3	WAS protein family homolog 1 OS=Gallus gallus GN=WASH1 PE=4 SV=1 - [F1NMK3_CHICK]	121.88	0.950	51.6	5.52
R4GL78	Platelet-activating factor acetylhydrolase IB subunit beta OS=Gallus gallus GN=PAFAH1B2 PE=4 SV=1 - [R4GL78_CHICK]	203.65	0.949	26.8	6.21
F1NYK9	Pannexin (Fragment) OS=Gallus gallus GN=PANX1 PE=3 SV=2 - [F1NYK9_CHICK]	53.31	0.949	46.7	6.87
P87362	Bleomycin hydrolase OS=Gallus gallus GN=BLMH PE=1 SV=1 - [BLMH_CHICK]	0.00	0.949	52.7	5.90
P20136	Glutathione S-transferase 2 OS=Gallus gallus GN=GSTM2 PE=1 SV=4 - [GSTM2_CHICK]	68.84	0.949	25.9	7.33
P52162	Protein max OS=Gallus gallus GN=MAX PE=3 SV=1 - [MAX_CHICK]	39.72	0.949	18.2	6.30
Q5ZL74	Vesicle-associated membrane protein 7 OS=Gallus gallus GN=VAMP7 PE=2 SV=1 - [VAMP7_CHICK]	0.00	0.949	24.7	8.22
F1NSH8	Adenosylhomocysteinase OS=Gallus gallus GN=AHCYL2 PE=3 SV=2 - [F1NSH8_CHICK]	98.58	0.949	56.7	7.99
Q5F3R2	Lysine-specific demethylase 5B OS=Gallus gallus GN=KDM5B PE=2 SV=1 - [KDM5B_CHICK]	0.00	0.948	173.3	6.28
Q5ZMJ0	Peptidyl-prolyl cis-trans isomerase OS=Gallus gallus GN=PPIF PE=2 SV=1 - [Q5ZMJ0_CHICK]	124.42	0.948	21.7	8.98

Q5F3Z3	Ubiquitin-conjugating enzyme E2 variant 2 OS=Gallus gallus GN=UBE2V2 PE=2 SV=1 - [UB2V2_CHICK]	62.81	0.948	16.3	8.09
P0CG62	Polyubiquitin-B OS=Gallus gallus GN=UBB PE=2 SV=1 - [UBB_CHICK]	389.91	0.948	34.3	7.53
E1C4N7	Sorting nexin OS=Gallus gallus GN=SNX18 PE=3 SV=2 - [E1C4N7_CHICK]	0.00	0.947	66.6	6.57
E1BVS1	E3 ubiquitin-protein ligase OS=Gallus gallus GN=ITCH PE=4 SV=2 - [E1BVS1_CHICK]	0.00	0.947	102.3	6.96
E1BWJ5	Ubiquitin carboxyl-terminal hydrolase OS=Gallus gallus GN=USP9X PE=3 SV=1 - [E1BWJ5_CHICK]	69.47	0.947	290.0	5.86
F1N954	Proteasome subunit alpha type (Fragment) OS=Gallus gallus GN=PSMA1 PE=3 SV=2 - [F1N954_CHICK]	165.94	0.947	29.3	6.54
F1NH88	Condensin complex subunit 1 OS=Gallus gallus GN=NCAPD2 PE=3 SV=2 - [F1NH88_CHICK]	0.00	0.947	157.6	5.74
Q5ZMQ0	Armadillo repeat-containing protein 1 OS=Gallus gallus GN=ARMC1 PE=2 SV=1 - [ARMC1_CHICK]	26.74	0.947	31.0	5.74
Q5ZKE7	UMP-CMP kinase OS=Gallus gallus GN=CMPK PE=2 SV=1 - [KCY_CHICK]	103.18	0.946	22.2	7.21
F1NB37	tRNA (guanine(10)-N2)-methyltransferase homolog OS=Gallus gallus GN=TRMT11 PE=4 SV=1 - [F1NB37_CHICK]	0.00	0.946	53.0	8.27
O13113	Dolichyl-diphosphooligosaccharide--protein glycosyltransferase subunit DAD1 OS=Gallus gallus GN=DAD1 PE=2 SV=1 - [DAD1_CHICK]	113.66	0.946	12.9	8.53
R4GFV6	Oxysterol-binding protein (Fragment) OS=Gallus gallus GN=OSBP PE=3 SV=1 - [R4GFV6_CHICK]	56.21	0.946	78.3	6.93
P10039-3	Isoform 3 of Tenascin OS=Gallus gallus GN=TNC - [TENA_CHICK]	190.63	0.945	168.8	5.24

O57535	Nucleoside diphosphate kinase OS=Gallus gallus PE=2 SV=1 - [NDK_CHICK]	160.95	0.945	17.3	7.90
E1C4Q0	Ribonucleoside-diphosphate reductase OS=Gallus gallus GN=RRM1 PE=3 SV=2 - [E1C4Q0_CHICK]	59.66	0.945	90.0	7.15
Q6R2T1	PS1D OS=Gallus gallus GN=ZCCHC17 PE=2 SV=1 - [Q6R2T1_CHICK]	0.00	0.945	26.8	9.48
F1NH22	Reticulon OS=Gallus gallus GN=RTN1 PE=4 SV=1 - [F1NH22_CHICK]	271.49	0.944	23.6	8.94
P02612	Myosin regulatory light chain 2, smooth muscle major isoform OS=Gallus gallus PE=1 SV=2 - [MLRM_CHICK]	15.85	0.944	19.8	4.92
R4GH17	Axin-1 (Fragment) OS=Gallus gallus GN=AXIN1 PE=4 SV=1 - [R4GH17_CHICK]	0.00	0.944	62.1	8.51
Q5ZLF4	Zinc transporter 5 OS=Gallus gallus GN=SLC30A5 PE=2 SV=1 - [ZNT5_CHICK]	0.00	0.944	84.7	7.42
Q5ZKP4	Trafficking protein particle complex subunit 2 OS=Gallus gallus GN=TRAPPC2 PE=2 SV=2 - [TPPC2_CHICK]	0.00	0.943	16.6	6.52
Q9W689	Ataxin-3 OS=Gallus gallus GN=ATXN3 PE=2 SV=1 - [ATX3_CHICK]	18.20	0.943	41.6	4.65
E1BWG0	Fatty acid synthase OS=Gallus gallus GN=FASN PE=4 SV=2 - [E1BWG0_CHICK]	452.91	0.943	274.6	6.34
Q5ZLQ4	Iron-responsive element-binding protein 2 OS=Gallus gallus GN=IREB2 PE=2 SV=1 - [IREB2_CHICK]	0.00	0.942	105.3	7.62
Q04929	Adapter molecule crk OS=Gallus gallus GN=CRK PE=1 SV=1 - [CRK_CHICK]	311.60	0.942	33.8	5.57
Q5ZIM6	Protein AATF OS=Gallus gallus GN=AATF PE=2 SV=1 - [AATF_CHICK]	0.00	0.942	65.0	4.94

A5HUJ4	Tripartite motif protein 7 OS=Gallus gallus GN=TRIM7.2 PE=4 SV=1 - [A5HUJ4_CHICK]	0.00	0.942	56.5	5.48
E1C6D1	Microtubule-associated protein OS=Gallus gallus GN=MAP2 PE=4 SV=2 - [E1C6D1_CHICK]	66.96	0.942	203.4	4.91
P18359	Destrin OS=Gallus gallus GN=DSTN PE=1 SV=3 - [DEST_CHICK]	611.79	0.942	18.5	7.59
E1BS26	Dolichyl pyrophosphate Man9GlcNAc2 alpha-1,3-glucosyltransferase OS=Gallus gallus GN=ALG6 PE=4 SV=2 - [E1BS26_CHICK]	0.00	0.942	57.4	9.01
Q07498-2	Isoform Short of Ephrin type-B receptor 3 OS=Gallus gallus GN=EPHB3 - [EPHB3_CHICK]	30.60	0.941	107.9	6.81
F1NFY3	Oxysterol-binding protein (Fragment) OS=Gallus gallus GN=OSBPL11 PE=3 SV=2 - [F1NFY3_CHICK]	134.08	0.940	78.7	6.67
P33150	Cadherin-13 OS=Gallus gallus GN=CDH13 PE=1 SV=1 - [CAD13_CHICK]	132.73	0.940	78.3	5.07
Q5ZLN1	Phosphoglycerate mutase 1 OS=Gallus gallus GN=PGAM1 PE=1 SV=3 - [PGAM1_CHICK]	598.26	0.940	28.9	7.49
F1NHT3	Spectrin alpha chain, non-erythrocytic 1 OS=Gallus gallus GN=SPTAN1 PE=4 SV=2 - [F1NHT3_CHICK]	451.70	0.940	285.2	5.33
Q5ZIP2	Ragulator complex protein LAMTOR3 OS=Gallus gallus GN=LAMTOR3 PE=2 SV=1 - [LTOR3_CHICK]	31.35	0.940	13.7	7.34
R4GI40	LDLR chaperone MESD OS=Gallus gallus GN=MESDC2 PE=4 SV=1 - [R4GI40_CHICK]	38.50	0.940	24.5	7.37
F1NSY1	Sodium/potassium-transporting ATPase subunit alpha-1 (Fragment) OS=Gallus gallus GN=ATP1A1 PE=3 SV=2 - [F1NSY1_CHICK]	191.30	0.940	112.0	5.62
P53033	Replication factor C subunit 2 OS=Gallus gallus GN=RFC2 PE=2 SV=1 - [RFC2_CHICK]	55.71	0.940	39.7	5.95

H9L168	Multivesicular body subunit 12A OS=Gallus gallus GN=FAM125A PE=4 SV=1 - [H9L168_CHICK]	0.00	0.940	28.0	8.82
E1BRG6	Plasma membrane calcium-transporting ATPase 1 OS=Gallus gallus GN=ATP2B1 PE=3 SV=2 - [E1BRG6_CHICK]	89.11	0.939	129.2	6.35
Q08393	Glutathione S-transferase OS=Gallus gallus PE=2 SV=2 - [GSTA2_CHICK]	52.67	0.939	25.4	8.73
F1NE50	Probable glutamate--tRNA ligase, mitochondrial OS=Gallus gallus GN=EARS2 PE=3 SV=1 - [F1NE50_CHICK]	16.19	0.939	56.9	6.87
F1NPD7	Proteasomal ATPase-associated factor 1 OS=Gallus gallus GN=PAAF1 PE=4 SV=1 - [F1NPD7_CHICK]	38.66	0.939	42.4	5.77
Q5ZJ03	Guanine nucleotide-binding protein subunit gamma OS=Gallus gallus GN=GNG2 PE=3 SV=1 - [Q5ZJ03_CHICK]	52.91	0.939	7.9	7.99
F1NZ49	Yorkie homolog (Fragment) OS=Gallus gallus GN=YAP1 PE=4 SV=2 - [F1NZ49_CHICK]	85.67	0.939	42.2	5.22
F1P526	Protein phosphatase methylesterase 1 OS=Gallus gallus GN=PPME1 PE=3 SV=2 - [F1P526_CHICK]	0.00	0.938	42.6	5.85
Q90635	Dihydropyrimidinase-related protein 2 OS=Gallus gallus GN=DPYSL2 PE=2 SV=1 - [DPYL2_CHICK]	361.79	0.938	62.3	6.38
F1N9U1	Serine/threonine-protein kinase TAO3 OS=Gallus gallus GN=TAOK3 PE=4 SV=1 - [F1N9U1_CHICK]	0.00	0.937	105.3	7.62
F1N9S7	Annexin OS=Gallus gallus GN=ANXA1 PE=3 SV=1 - [F1N9S7_CHICK]	295.50	0.937	38.5	7.44
F1NSV1	Sulfhydryl oxidase OS=Gallus gallus GN=GFER PE=3 SV=2 - [F1NSV1_CHICK]	0.00	0.937	21.4	6.21

F1NKI1	Cartilage-associated protein OS=Gallus gallus GN=CRTAP PE=4 SV=2 - [F1NKI1_CHICK]	0.00	0.936	46.0	5.16
F1NZC2	AP-3 complex subunit mu-1 OS=Gallus gallus GN=AP3M1 PE=4 SV=1 - [F1NZC2_CHICK]	48.42	0.936	47.0	6.95
Q5ZMT0	14-3-3 protein epsilon OS=Gallus gallus GN=YWHAE PE=1 SV=1 - [1433E_CHICK]	762.30	0.936	29.2	4.74
R4GMG8	Cathepsin K (Fragment) OS=Gallus gallus GN=CTSK PE=3 SV=1 - [R4GMG8_CHICK]	39.87	0.936	36.7	9.25
F1N9M3	Centromere protein Q OS=Gallus gallus GN=CENPQ PE=4 SV=1 - [F1N9M3_CHICK]	0.00	0.936	32.2	9.17
Q5ZKY2	Ubiquitin-like modifier-activating enzyme ATG7 OS=Gallus gallus GN=ATG7 PE=2 SV=1 - [ATG7_CHICK]	0.00	0.935	78.7	6.54
Q9W6F3	Ubiquitin-conjugating enzyme OS=Gallus gallus GN=UBE2A PE=2 SV=1 - [Q9W6F3_CHICK]	27.09	0.935	17.3	5.15
F1N9H3	Protein disulfide-isomerase OS=Gallus gallus GN=P4HB PE=3 SV=2 - [F1N9H3_CHICK]	1418.28	0.934	55.8	4.88
P00337	L-lactate dehydrogenase B chain OS=Gallus gallus GN=LDHB PE=1 SV=3 - [LDHB_CHICK]	102.00	0.934	36.3	7.43
F1NRN7	Leucine-rich repeat protein SHOC-2 OS=Gallus gallus GN=SHOC2 PE=4 SV=1 - [F1NRN7_CHICK]	0.00	0.934	59.1	8.72
P02467	Collagen alpha-2(I) chain (Fragments) OS=Gallus gallus GN=COL1A2 PE=1 SV=2 - [CO1A2_CHICK]	78.56	0.934	129.6	8.94
Q5F3W5	Histone-lysine N-methyltransferase SUV39H2 OS=Gallus gallus GN=SUV39H2 PE=2 SV=1 - [SUV92_CHICK]	0.00	0.934	46.6	8.27
Q5ZJS8	Galectin OS=Gallus gallus GN=LGALS8 PE=2 SV=1 - [Q5ZJS8_CHICK]	0.00	0.933	33.7	8.07
F1NLV9	DNA-directed RNA polymerase OS=Gallus gallus GN=Gga.56030 PE=3 SV=2 - [F1NLV9_CHICK]	0.00	0.933	125.6	7.81

F1NW80	Golgi SNAP receptor complex member 1 OS=Gallus gallus GN=GOSR1 PE=3 SV=2 - [F1NW80_CHICK]	34.29	0.933	28.0	9.45
E1C8W4	Ubiquitin carboxyl-terminal hydrolase OS=Gallus gallus GN=USP5 PE=3 SV=2 - [E1C8W4_CHICK]	269.32	0.933	95.7	5.11
F1NVX3	Alpha-(1,3)-fucosyltransferase 11 OS=Gallus gallus GN=FUT11 PE=3 SV=1 - [F1NVX3_CHICK]	0.00	0.933	57.5	7.24
F1NY25	Cytoplasmic aconitate hydratase OS=Gallus gallus GN=ACO1 PE=4 SV=1 - [F1NY25_CHICK]	0.00	0.933	98.0	7.33
Q5ZME2	Malate dehydrogenase, cytoplasmic OS=Gallus gallus GN=MDH1 PE=2 SV=1 - [MDHC_CHICK]	136.49	0.932	36.5	7.36
F1NNY1	Casein kinase I isoform alpha OS=Gallus gallus GN=CSNK1A1 PE=4 SV=1 - [F1NNY1_CHICK]	89.17	0.932	38.7	9.57
Q5ZMH1	Septin-2 OS=Gallus gallus GN=SEPT2 PE=2 SV=1 - [SEPT2_CHICK]	206.76	0.931	40.2	6.55
F1P046	Spondin-1 OS=Gallus gallus GN=SPON1 PE=4 SV=2 - [F1P046_CHICK]	0.00	0.931	90.4	6.60
F1NE88	Ubiquitin carboxyl-terminal hydrolase OS=Gallus gallus GN=USP4 PE=3 SV=1 - [F1NE88_CHICK]	42.15	0.931	107.4	5.83
D5M8S2	DJ-1 OS=Gallus gallus GN=DJ-1 PE=2 SV=1 - [D5M8S2_CHICK]	85.12	0.931	19.9	6.77
F1NAX3	Phosphoglycolate phosphatase (Fragment) OS=Gallus gallus GN=PGP PE=4 SV=2 - [F1NAX3_CHICK]	0.00	0.931	19.8	4.68
R4GLV0	Coronin OS=Gallus gallus GN=LOC776194 PE=3 SV=1 - [R4GLV0_CHICK]	29.63	0.930	42.8	6.60
Q5ZJ39	Density-regulated protein OS=Gallus gallus GN=DENR PE=2 SV=1 - [DENR_CHICK]	36.69	0.929	22.1	5.21
Q91012-2	Isoform 2 of TSC22 domain family protein 1 OS=Gallus gallus GN=TSC22D1 - [T22D1_CHICK]	0.00	0.929	15.3	5.19

P21872-2	Isoform Short of Trifunctional purine biosynthetic protein adenosine-3 OS=Gallus gallus GN=GART - [PUR2_CHICK]	35.52	0.929	46.0	7.87
F1NTM7	Aspartate aminotransferase OS=Gallus gallus GN=GOT1 PE=3 SV=2 - [F1NTM7_CHICK]	236.93	0.928	45.9	8.12
F1NV24	Syndecan (Fragment) OS=Gallus gallus GN=SDC1 PE=3 SV=1 - [F1NV24_CHICK]	0.00	0.927	33.3	4.70
Q5ZLT7	Basic leucine zipper and W2 domain-containing protein 1 OS=Gallus gallus GN=BZW1 PE=2 SV=1 - [BZW1_CHICK]	89.35	0.927	48.0	5.92
Q5ZL13	Nucleoside diphosphate-linked moiety X motif 19, mitochondrial OS=Gallus gallus GN=NUDT19 PE=2 SV=1 - [NUD19_CHICK]	0.00	0.927	42.1	6.86
Q5ZJA9	Loss of heterozygosity 12 chromosomal region 1 protein homolog OS=Gallus gallus GN=LOH12CR1 PE=2 SV=1 - [L12R1_CHICK]	0.00	0.927	20.9	7.44
Q92074	Beta-1,4-galactosyltransferase OS=Gallus gallus GN=CKI PE=2 SV=1 - [Q92074_CHICK]	58.05	0.926	40.9	8.32
F1NJU7	Tyrosine--tRNA ligase, cytoplasmic OS=Gallus gallus GN=YARS PE=4 SV=2 - [F1NJU7_CHICK]	98.79	0.926	59.3	6.65
Q90W83	Aldo-keto reductase OS=Gallus gallus GN=akr PE=2 SV=1 - [Q90W83_CHICK]	200.94	0.925	36.4	7.75
F1NI43	Long-chain-fatty-acid--CoA ligase ACSBG2 OS=Gallus gallus GN=ACSBG2 PE=4 SV=2 - [F1NI43_CHICK]	212.43	0.924	83.9	7.12
Q90694	Cell division control protein 42 homolog OS=Gallus gallus GN=CDC42 PE=2 SV=1 - [CDC42_CHICK]	43.47	0.924	21.3	6.55
Q5ZI60	Coronin OS=Gallus gallus GN=CORO1C PE=2 SV=1 - [Q5ZI60_CHICK]	81.39	0.924	53.2	6.67

C6KGD4	Progestin and adipoQ receptor family member VII OS=Gallus gallus GN=PAQR7 PE=2 SV=1 - [C6KGD4_CHICK]	39.87	0.924	39.7	8.37
Q1G1I6	PACSIN 3 OS=Gallus gallus GN=PACSIN3 PE=2 SV=1 - [Q1G1I6_CHICK]	0.00	0.924	50.7	6.00
E1BRE9	Decorin OS=Gallus gallus GN=DCN PE=4 SV=2 - [E1BRE9_CHICK]	28.78	0.923	39.6	8.32
F1NZ78	Alpha-enolase OS=Gallus gallus GN=ENO1 PE=3 SV=2 - [F1NZ78_CHICK]	1378.88	0.923	47.3	6.80
E1BTT8	L-lactate dehydrogenase OS=Gallus gallus GN=LDHA PE=3 SV=2 - [E1BTT8_CHICK]	491.72	0.923	36.5	7.90
F1NPF0	Eukaryotic translation initiation factor 2 subunit 3 OS=Gallus gallus GN=EIF2S3 PE=4 SV=1 - [F1NPF0_CHICK]	148.24	0.923	51.1	8.27
R4GM10	Fructose-bisphosphate aldolase OS=Gallus gallus GN=ALDOC PE=3 SV=1 - [R4GM10_CHICK]	1950.09	0.922	39.3	6.64
Q5ZLL9	Breast cancer metastasis-suppressor 1-like protein OS=Gallus gallus GN=BRMS1L PE=2 SV=1 - [BRM1L_CHICK]	0.00	0.922	37.7	5.15
P84172	Elongation factor Tu, mitochondrial (Fragment) OS=Gallus gallus GN=TUFM PE=1 SV=1 - [EFTU_CHICK]	182.04	0.921	38.2	8.76
P00174	Cytochrome b5 OS=Gallus gallus GN=CYB5A PE=1 SV=4 - [CYB5_CHICK]	58.16	0.921	15.5	5.15
F1NF77	Vacuolar protein sorting-associated protein 53 homolog (Fragment) OS=Gallus gallus GN=VPS53 PE=4 SV=2 - [F1NF77_CHICK]	30.86	0.921	91.8	7.84
F1NBT4	Ferrochelatase OS=Gallus gallus GN=FECH PE=3 SV=1 - [F1NBT4_CHICK]	0.00	0.920	45.4	8.91

Q90744	Alpha-N-acetylgalactosaminidase OS=Gallus gallus GN=NAGA PE=1 SV=1 - [NAGAB_CHICK]	85.32	0.919	45.6	6.09
F1P053	DnaJ homolog subfamily C member 3 OS=Gallus gallus GN=DNAJC3 PE=4 SV=1 - [F1P053_CHICK]	117.95	0.918	57.4	5.97
P28497	F-actin-capping protein subunit alpha-2 OS=Gallus gallus GN=CAPZA2 PE=1 SV=1 - [CAZA2_CHICK]	44.43	0.917	32.8	5.82
Q5ZKX6	NEDD8-conjugating enzyme UBE2F OS=Gallus gallus GN=UBE2F PE=2 SV=1 - [UBE2F_CHICK]	31.13	0.917	21.2	6.68
F1P470	D-dopachrome decarboxylase OS=Gallus gallus GN=DDT PE=4 SV=1 - [F1P470_CHICK]	52.11	0.917	12.8	7.14
F1NE60	Sodium/hydrogen exchanger OS=Gallus gallus GN=SLC9A1 PE=3 SV=2 - [F1NE60_CHICK]	0.00	0.917	77.1	6.67
F1NSX8	Cytochrome c-type heme lyase OS=Gallus gallus GN=HCCS PE=4 SV=1 - [F1NSX8_CHICK]	0.00	0.917	31.4	6.54
P14092	ATP synthase subunit a OS=Gallus gallus GN=MT-ATP6 PE=3 SV=1 - [ATP6_CHICK]	25.58	0.916	24.8	9.38
Q5ZHQ5	Cholinephosphotransferase 1 OS=Gallus gallus GN=CHPT1 PE=2 SV=1 - [CHPT1_CHICK]	28.17	0.915	37.1	6.67
P08836	Farnesyl pyrophosphate synthase OS=Gallus gallus GN=FDPS PE=1 SV=2 - [FPPS_CHICK]	323.84	0.915	42.1	6.57
F1NCK0	Reticulon OS=Gallus gallus GN=RTN4 PE=4 SV=1 - [F1NCK0_CHICK]	79.20	0.914	22.3	9.19
F1NB02	40S ribosomal protein S8 OS=Gallus gallus GN=RPS8 PE=3 SV=2 - [F1NB02_CHICK]	111.99	0.914	24.2	10.32
Q9DER7	Tolloid-like protein 1 OS=Gallus gallus GN=TLL1 PE=2 SV=1 - [TLL1_CHICK]	24.99	0.913	114.8	6.32
F1P593	Heat shock protein beta-1 OS=Gallus gallus GN=HSPB1 PE=3 SV=2 - [F1P593_CHICK]	84.93	0.913	21.8	6.70

F1NTK1	Phosphoserine aminotransferase OS=Gallus gallus GN=PSAT1 PE=3 SV=2 - [F1NTK1_CHICK]	94.48	0.913	40.5	8.63
F1NGP4	tRNA-dihydrouridine(47) synthase [NAD(P)(+)] (Fragment) OS=Gallus gallus GN=DUS3L PE=3 SV=1 - [F1NGP4_CHICK]	0.00	0.912	71.3	6.98
Q5ZJJ9	Osteoclast-stimulating factor 1 OS=Gallus gallus GN=OSTF1 PE=2 SV=1 - [OSTF1_CHICK]	75.79	0.912	22.3	8.13
P05081	Adenylate kinase isoenzyme 1 OS=Gallus gallus GN=AK1 PE=1 SV=1 - [KAD1_CHICK]	102.67	0.912	21.7	8.59
F1NJG4	Cytochrome P450 CYP2D49 OS=Gallus gallus GN=Gga.10540 PE=2 SV=2 - [F1NJG4_CHICK]	0.00	0.911	57.9	8.72
Q5ZKZ9	Lipase maturation factor 2 OS=Gallus gallus GN=LMF2 PE=2 SV=2 - [LMF2_CHICK]	37.69	0.911	81.5	9.60
F1NLV8	Inactive tyrosine-protein kinase 7 OS=Gallus gallus GN=PTK7 PE=4 SV=1 - [F1NLV8_CHICK]	59.51	0.911	115.9	7.05
Q5ZJ73	Protein CREG1 OS=Gallus gallus GN=CREG1 PE=2 SV=1 - [CREG1_CHICK]	50.85	0.911	21.1	6.67
Q49B65	EF hand-containing protein 1 OS=Gallus gallus GN=EFHD1 PE=2 SV=1 - [Q49B65_CHICK]	106.72	0.910	26.9	5.11
Q5ZJ64	Eukaryotic translation initiation factor 3 subunit M OS=Gallus gallus GN=EIF3M PE=2 SV=1 - [EIF3M_CHICK]	121.01	0.909	42.6	5.53
F1NIY3	Programmed cell death protein 4 OS=Gallus gallus GN=PDCD4 PE=4 SV=1 - [F1NIY3_CHICK]	0.00	0.908	51.7	5.49
F1P2Q5	Fatty acid synthase OS=Gallus gallus GN=FAS PE=4 SV=2 - [F1P2Q5_CHICK]	0.00	0.908	38.4	6.93
Q5ZL35	Arginine and glutamate-rich protein 1 OS=Gallus gallus GN=ARGLU1 PE=2 SV=1 - [ARGL1_CHICK]	28.27	0.908	33.5	10.32

Q02960	Macrophage migration inhibitory factor OS=Gallus gallus GN=MIF PE=3 SV=3 - [MIF_CHICK]	485.40	0.908	12.5	7.37
P02457	Collagen alpha-1(I) chain OS=Gallus gallus GN=COL1A1 PE=1 SV=3 - [CO1A1_CHICK]	164.16	0.907	137.7	5.62
E1C6J9	Thy-1 membrane glycoprotein OS=Gallus gallus GN=THY1 PE=4 SV=2 - [E1C6J9_CHICK]	174.94	0.907	18.2	8.02
E1BZ85	Meiosis arrest female protein 1 homolog OS=Gallus gallus GN=MARF1 PE=3 SV=1 - [MARF1_CHICK]	0.00	0.907	193.2	7.71
Q5F428	Eukaryotic translation initiation factor 3 subunit L OS=Gallus gallus GN=EIF3L PE=2 SV=1 - [EIF3L_CHICK]	192.26	0.907	66.6	6.34
F1NF68	Solute carrier family 2, facilitated glucose transporter member 1 (Fragment) OS=Gallus gallus GN=SLC2A1 PE=3 SV=1 - [F1NF68_CHICK]	15.14	0.907	53.7	8.21
F2Z4L6	Serum albumin OS=Gallus gallus GN=ALB PE=4 SV=2 - [F2Z4L6_CHICK]	0.00	0.906	64.0	5.45
P31395	Stathmin OS=Gallus gallus GN=STMN1 PE=1 SV=1 - [STMN1_CHICK]	165.81	0.906	17.1	6.58
F1NQF4	Heme oxygenase 1 OS=Gallus gallus GN=HMOX1 PE=4 SV=2 - [F1NQF4_CHICK]	346.99	0.905	33.5	8.43
F1NZY6	CUGBP Elav-like family member 2 OS=Gallus gallus GN=CELF2 PE=4 SV=2 - [F1NZY6_CHICK]	18.54	0.905	48.6	8.98
F2Z4M7	60S ribosomal protein L37a (Fragment) OS=Gallus gallus GN=RPL37A PE=3 SV=1 - [F2Z4M7_CHICK]	135.13	0.905	10.1	10.36
F1NM13	Ankyrin repeat and MYND domain-containing protein 2 OS=Gallus gallus GN=ANKMY2 PE=4 SV=1 - [F1NM13_CHICK]	42.70	0.904	51.6	6.24

O73871	Microphthalmia-associated transcription factor isoform B OS=Gallus gallus GN=cmi9 PE=2 SV=1 - [O73871_CHICK]	0.00	0.904	52.4	6.67
Q5ZKE5	45 kDa calcium-binding protein OS=Gallus gallus GN=SDF4 PE=2 SV=2 - [CAB45_CHICK]	113.37	0.904	41.9	4.72
F1NUT5	Adenosylhomocysteinase OS=Gallus gallus GN=AHCYL1 PE=3 SV=2 - [F1NUT5_CHICK]	95.09	0.904	53.7	7.44
O73930	Pterin-4-alpha-carbinolamine dehydratase OS=Gallus gallus GN=PCBD1 PE=2 SV=3 - [PHS_CHICK]	37.77	0.904	12.0	6.52
P21642	Phosphoenolpyruvate carboxykinase [GTP], mitochondrial OS=Gallus gallus GN=PCK2 PE=1 SV=2 - [PCKGM_CHICK]	34.83	0.904	71.1	7.61
E1BSC6	Oxysterol-binding protein OS=Gallus gallus GN=OSBPL5 PE=3 SV=2 - [E1BSC6_CHICK]	0.00	0.903	100.7	8.03
P00356	Glyceraldehyde-3-phosphate dehydrogenase OS=Gallus gallus GN=GAPDH PE=2 SV=3 - [G3P_CHICK]	2394.51	0.903	35.7	8.54
R9PXN7	Hematopoietic prostaglandin D synthase OS=Gallus gallus GN=HPGDS PE=3 SV=1 - [R9PXN7_CHICK]	71.73	0.903	22.6	7.28
Q5ZIZ0	6-phosphogluconate dehydrogenase, decarboxylating OS=Gallus gallus GN=PGD PE=2 SV=1 - [Q5ZIZ0_CHICK]	390.04	0.903	53.3	6.98
E1BS67	Serine hydroxymethyltransferase (Fragment) OS=Gallus gallus GN=SHMT1 PE=3 SV=2 - [E1BS67_CHICK]	84.68	0.903	58.2	8.57
A6NAB8	Delta-6 fatty acid desaturase OS=Gallus gallus GN=FADS2 PE=2 SV=2 - [A6NAB8_CHICK]	0.00	0.902	52.0	8.84
P28693-2	Isoform Short of Ephrin type-B receptor 2 OS=Gallus gallus GN=EPHB2 - [EPHB2_CHICK]	23.32	0.902	110.2	5.64

P07341	Fructose-bisphosphate aldolase B OS=Gallus gallus GN=ALDOB PE=3 SV=3 - [ALDOB_CHICK]	70.45	0.902	39.3	8.59
F1P1S6	Actin-related protein 2/3 complex subunit 5 (Fragment) OS=Gallus gallus PE=3 SV=1 - [F1P1S6_CHICK]	59.05	0.901	11.8	10.15
F1NIJ6	Glucose-6-phosphate isomerase OS=Gallus gallus GN=GPI PE=3 SV=1 - [F1NIJ6_CHICK]	390.39	0.901	62.1	7.96
F1ND11	Protein arginine N-methyltransferase 7 OS=Gallus gallus GN=PRMT7 PE=4 SV=1 - [F1ND11_CHICK]	0.00	0.900	78.0	5.87
F1P3A9	Methylsterol monooxygenase 1 OS=Gallus gallus GN=SC4MOL PE=4 SV=1 - [F1P3A9_CHICK]	40.99	0.900	35.5	7.08
P07032	Acylphosphatase-1 OS=Gallus gallus GN=ACYP1 PE=1 SV=2 - [ACYP1_CHICK]	47.04	0.900	11.1	9.86
F1P006	Toll-interacting protein OS=Gallus gallus GN=TOLLIP PE=4 SV=1 - [F1P006_CHICK]	71.62	0.900	30.6	5.16
A4F5B6	TAP binding protein (Precursor) OS=Gallus gallus GN=TPN PE=2 SV=1 - [A4F5B6_CHICK]	34.63	0.899	45.5	8.65
E1BXQ0	Ubiquitin carboxyl-terminal hydrolase OS=Gallus gallus GN=USP18 PE=3 SV=1 - [E1BXQ0_CHICK]	41.01	0.899	43.7	8.10
E1C1V3	Plakoglobin OS=Gallus gallus GN=JUP PE=2 SV=2 - [E1C1V3_CHICK]	85.89	0.898	82.1	6.14
F1NCK8	Glycophorin C OS=Gallus gallus GN=GYPC PE=4 SV=2 - [F1NCK8_CHICK]	33.89	0.898	10.1	5.34
E1C735	S-adenosylmethionine synthase OS=Gallus gallus GN=MAT1A PE=3 SV=1 - [E1C735_CHICK]	44.14	0.897	43.7	6.76
Q9I8D1-2	Isoform 2 of Unconventional myosin-VI OS=Gallus gallus GN=MYO6 - [MYO6_CHICK]	33.81	0.897	144.9	8.60
F1NVM9	Elastin OS=Gallus gallus GN=ELN PE=4 SV=2 - [F1NVM9_CHICK]	16.91	0.895	58.6	10.51

F1P3Z0	Protein argonaute-3 OS=Gallus gallus GN=EIF2C3 PE=3 SV=1 - [F1P3Z0_CHICK]	19.90	0.895	97.3	9.11
P79996	Mitogen-activated protein kinase 9 OS=Gallus gallus GN=MAPK9 PE=2 SV=1 - [MK09_CHICK]	0.00	0.893	44.0	6.39
Q5F359	Trafficking protein particle complex subunit 5 OS=Gallus gallus GN=TRAPPC5 PE=2 SV=1 - [TPPC5_CHICK]	0.00	0.892	21.0	9.29
Q90WI4	Matrix-remodeling-associated protein 8 OS=Gallus gallus GN=MXRA8 PE=2 SV=1 - [MXRA8_CHICK]	42.40	0.890	50.7	7.77
Q5ZIU8	Katanin p80 WD40 repeat-containing subunit B1 OS=Gallus gallus GN=KATNB1 PE=2 SV=2 - [KTNB1_CHICK]	0.00	0.890	72.7	7.59
F1NW43	Pyruvate kinase OS=Gallus gallus GN=PKM2 PE=3 SV=2 - [F1NW43_CHICK]	740.98	0.890	57.8	7.87
Q5ZLK5	Prolyl 4-hydroxylase subunit alpha-2 OS=Gallus gallus GN=P4HA2 PE=2 SV=1 - [P4HA2_CHICK]	119.17	0.888	61.4	6.11
F1P5J8	N-acetylglucosamine-6-phosphate deacetylase (Fragment) OS=Gallus gallus GN=AMDHD2 PE=3 SV=1 - [F1P5J8_CHICK]	39.78	0.887	45.6	6.80
F1NF81	Acyl-CoA-binding protein (Fragment) OS=Gallus gallus GN=DBI PE=4 SV=1 - [F1NF81_CHICK]	510.46	0.887	9.6	8.31
Q5ZI04	Protein FAM49A OS=Gallus gallus GN=FAM49A PE=2 SV=1 - [FA49A_CHICK]	57.71	0.885	37.3	6.02
Q90997	Transferrin receptor protein 1 OS=Gallus gallus GN=TFRC PE=2 SV=2 - [TFR1_CHICK]	100.91	0.885	85.6	5.78
Q5F352	GTPase NRas OS=Gallus gallus GN=NRAS PE=2 SV=1 - [RASN_CHICK]	50.10	0.885	21.3	5.06

P30997	Catenin alpha-2 OS=Gallus gallus GN=CTNNA2 PE=1 SV=1 - [CTNA2_CHICK]	62.07	0.885	100.6	6.29
F1NM17	Integral membrane protein 2B OS=Gallus gallus GN=ITM2B PE=4 SV=1 - [F1NM17_CHICK]	0.00	0.883	30.0	5.53
A1IMF0	Ubiquitin carboxyl-terminal hydrolase isozyme L1 OS=Gallus gallus GN=UCH-L1 PE=2 SV=1 - [A1IMF0_CHICK]	56.63	0.882	25.1	6.07
F1NKX4	Protein MIS12 homolog OS=Gallus gallus GN=MIS12 PE=4 SV=1 - [F1NKX4_CHICK]	0.00	0.882	24.7	5.73
F1P3X5	tRNA pseudouridine synthase OS=Gallus gallus GN=PUS3 PE=3 SV=2 - [F1P3X5_CHICK]	0.00	0.881	52.1	8.25
Q07816-2	Isoform Short of Bcl-2-like protein 1 OS=Gallus gallus GN=BCL2L1 - [B2CL1_CHICK]	33.09	0.881	21.5	5.06
E1BY52	Prolyl 4-hydroxylase subunit alpha-1 OS=Gallus gallus GN=P4HA1 PE=4 SV=2 - [E1BY52_CHICK]	357.68	0.880	61.5	5.76
Q5ZK09	Protein Hikeshi OS=Gallus gallus GN=RCJMB04_13p7 PE=2 SV=2 - [HIKES_CHICK]	44.61	0.880	21.8	5.20
P01012	Ovalbumin OS=Gallus gallus GN=SERPINB14 PE=1 SV=2 - [OVAL_CHICK]	0.00	0.879	42.9	5.29
Q5F3X0	Lysocardiolipin acyltransferase 1 OS=Gallus gallus GN=LCLAT1 PE=2 SV=1 - [LCLT1_CHICK]	34.88	0.879	44.4	8.57
P35915	Hydroxymethylglutaryl-CoA lyase, mitochondrial OS=Gallus gallus GN=HMGCL PE=1 SV=1 - [HMGCL_CHICK]	35.70	0.879	31.4	7.25
R4GFT9	Guanine nucleotide-binding protein subunit gamma OS=Gallus gallus GN=GNG12 PE=3 SV=1 - [R4GFT9_CHICK]	45.84	0.878	8.1	9.25

F1N9I7	Probable carboxypeptidase PM20D1 OS=Gallus gallus GN=PM20D1 PE=4 SV=1 - [F1N9I7_CHICK]	43.52	0.878	57.1	7.21
F1NF23	Oxysterol-binding protein OS=Gallus gallus GN=OSBPL6 PE=3 SV=2 - [F1NF23_CHICK]	0.00	0.877	110.8	7.15
Q5ZHK9	Protein LLP homolog OS=Gallus gallus GN=LLPH PE=3 SV=1 - [LLPH_CHICK]	46.73	0.876	13.4	10.42
P07583	Beta-galactoside-binding lectin OS=Gallus gallus PE=1 SV=2 - [LEG4_CHICK]	648.06	0.876	15.1	7.09
F1NM57	Acyl-CoA-binding domain-containing protein 5 OS=Gallus gallus GN=ACBD5 PE=3 SV=1 - [F1NM57_CHICK]	36.82	0.876	54.4	5.20
F1N8Q1	Superoxide dismutase [Cu-Zn] OS=Gallus gallus GN=SOD1 PE=3 SV=2 - [F1N8Q1_CHICK]	257.00	0.875	15.7	6.60
F1NEX5	Beta-hexosaminidase OS=Gallus gallus GN=HEXA PE=3 SV=1 - [F1NEX5_CHICK]	59.80	0.874	58.9	6.54
R4GGI6	Methyltransferase-like protein 14 OS=Gallus gallus GN=METTL14 PE=4 SV=1 - [R4GGI6_CHICK]	0.00	0.874	50.8	5.49
E1BXE8	Transporter OS=Gallus gallus GN=SLC6A11 PE=3 SV=1 - [E1BXE8_CHICK]	0.00	0.873	69.8	6.43
P12105	Collagen alpha-1(III) chain (Fragments) OS=Gallus gallus GN=COL3A1 PE=2 SV=2 - [CO3A1_CHICK]	74.04	0.872	121.2	6.70
E1BUA8	Protein BCCIP homolog (Fragment) OS=Gallus gallus GN=BCCIP PE=3 SV=2 - [E1BUA8_CHICK]	42.38	0.872	35.9	4.91
F1NNN2	Serine/threonine-protein phosphatase 2A 55 kDa regulatory subunit B OS=Gallus gallus GN=PPP2R2D PE=3 SV=2 - [F1NNN2_CHICK]	0.00	0.872	52.0	6.49
E1BR38	Argininosuccinate synthase OS=Gallus gallus GN=ASS1 PE=3 SV=2 - [E1BR38_CHICK]	0.00	0.872	46.9	6.54

E1BSP1	Proactivator polypeptide OS=Gallus gallus GN=PSAP PE=4 SV=1 - [E1BSP1_CHICK]	231.42	0.871	57.5	5.16
P19753	Parvalbumin, thymic OS=Gallus gallus PE=1 SV=2 - [PRVT_CHICK]	0.00	0.868	11.7	4.68
Q5ZHM5	Protein RER1 OS=Gallus gallus GN=RER1 PE=2 SV=1 - [RER1_CHICK]	44.96	0.867	23.0	9.51
F1NFG8	tRNA (guanine(37)-N1)-methyltransferase OS=Gallus gallus GN=TRMT5 PE=3 SV=2 - [F1NFG8_CHICK]	0.00	0.867	58.5	8.29
F1NZC0	Aldose 1-epimerase OS=Gallus gallus GN=GALM PE=3 SV=2 - [F1NZC0_CHICK]	21.88	0.867	38.0	6.89
Q05916	Homeobox protein engrailed-1 OS=Gallus gallus GN=EN1 PE=3 SV=1 - [HME1_CHICK]	95.24	0.865	34.5	9.51
P05300	Lysosome-associated membrane glycoprotein 1 OS=Gallus gallus GN=LAMP1 PE=2 SV=1 - [LAMP1_CHICK]	129.07	0.865	44.6	7.02
Q05423	Fatty acid-binding protein, brain OS=Gallus gallus GN=FABP7 PE=2 SV=2 - [FABP7_CHICK]	286.68	0.863	14.9	5.91
Q5ZI42	Malignant T-cell-amplified sequence 1 OS=Gallus gallus GN=MCTS1 PE=2 SV=1 - [MCTS1_CHICK]	0.00	0.863	20.6	8.82
Q90WG1	Spindlin-Z OS=Gallus gallus GN=SPINZ PE=2 SV=1 - [SPINZ_CHICK]	41.15	0.862	29.6	7.02
Q646T7	FK-506 binding protein 51 OS=Gallus gallus GN=FKBP51 PE=2 SV=1 - [Q646T7_CHICK]	85.27	0.861	50.4	6.21
F1NTI1	Coiled-coil domain-containing protein 80 OS=Gallus gallus GN=CCDC80 PE=4 SV=2 - [F1NTI1_CHICK]	40.63	0.861	95.9	9.86
O93510	Gelsolin OS=Gallus gallus GN=GSN PE=2 SV=1 - [GELS_CHICK]	415.97	0.859	85.8	6.32
E1C4H1	Trafficking protein particle complex subunit 11 OS=Gallus gallus GN=TRAPPC11 PE=4 SV=2 -	0.00	0.859	128.4	7.53

	[E1C4H1_CHICK]				
F1NMB9	Heme-binding protein 1 OS=Gallus gallus GN=HEBP1 PE=4 SV=1 - [F1NMB9_CHICK]	33.21	0.858	21.1	6.73
F1NU17	Phosphoglycerate kinase OS=Gallus gallus GN=PGK1 PE=3 SV=1 - [F1NU17_CHICK]	919.47	0.857	44.6	8.12
P08070	Tubulin alpha-2 chain OS=Gallus gallus PE=2 SV=1 - [TBA2_CHICK]	223.40	0.854	49.8	5.01
Q9YH69	Dermo protein OS=Gallus gallus GN=cdermo-1 PE=2 SV=1 - [Q9YH69_CHICK]	56.89	0.854	18.2	9.47
P21611	Beta-2-microglobulin OS=Gallus gallus GN=B2M PE=1 SV=3 - [B2MG_CHICK]	46.92	0.852	13.0	6.28
P00378	Dihydrofolate reductase OS=Gallus gallus GN=DHFR PE=1 SV=1 - [DYR_CHICK]	36.00	0.852	21.6	8.07
P98157-2	Isoform 2 of Low-density lipoprotein receptor-related protein 1 OS=Gallus gallus GN=LRP1 - [LRP1_CHICK]	223.12	0.852	506.3	5.26
R4GKA6	Collagen alpha-2(VI) chain OS=Gallus gallus GN=COL6A2 PE=4 SV=1 - [R4GKA6_CHICK]	181.18	0.850	102.4	5.48
E1BXS2	Guanine nucleotide-binding protein G(i) subunit alpha-1 OS=Gallus gallus GN=GNAI1 PE=4 SV=2 - [E1BXS2_CHICK]	147.69	0.850	40.4	5.97
Q90927	Nuclear factor 1 OS=Gallus gallus GN=cNFI-A4 PE=2 SV=1 - [Q90927_CHICK]	0.00	0.850	54.6	8.31
E1BUI0	tRNA pseudouridine synthase (Fragment) OS=Gallus gallus GN=PUSL1 PE=3 SV=2 - [E1BUI0_CHICK]	55.04	0.849	33.7	9.64
Q90617-3	Isoform LAMP-2C of Lysosome-associated membrane glycoprotein 2 OS=Gallus gallus GN=LAMP2 -	149.29	0.849	46.4	6.43

	[LAMP2_CHICK]				
Q90733	COUP transcription factor 2 OS=Gallus gallus GN=NR2F2 PE=2 SV=1 - [COT2_CHICK]	0.00	0.848	45.4	8.28
P12957-2	Isoform Brain l-cad of Caldesmon OS=Gallus gallus GN=CALD1 - [CALD1_CHICK]	0.00	0.847	58.8	8.44
F1N9D8	Cathepsin B OS=Gallus gallus GN=CTSB PE=3 SV=2 - [F1N9D8_CHICK]	133.09	0.847	37.6	5.86
A4GTP0	Galectin OS=Gallus gallus PE=2 SV=1 - [A4GTP0_CHICK]	66.44	0.846	25.7	8.27
Q08392	Glutathione S-transferase OS=Gallus gallus PE=2 SV=1 - [GSTA1_CHICK]	108.74	0.846	25.3	8.88
F1N965	Frizzled-7 OS=Gallus gallus GN=FZD7 PE=4 SV=1 - [F1N965_CHICK]	0.00	0.842	62.7	7.99
E1C3U7	Lysyl oxidase homolog 2 OS=Gallus gallus GN=LOXL2 PE=3 SV=1 - [LOXL2_CHICK]	0.00	0.842	86.9	6.49
F1NGX1	Integrin alpha-V OS=Gallus gallus GN=ITGAV PE=3 SV=1 - [F1NGX1_CHICK]	172.35	0.841	114.3	5.58
E1BRJ4	DNA-directed RNA polymerase OS=Gallus gallus GN=POLR3B PE=3 SV=1 - [E1BRJ4_CHICK]	0.00	0.840	127.4	8.54
Q8AXV1	Endophilin-A1 OS=Gallus gallus GN=SH3GL2 PE=1 SV=1 - [SH3G2_CHICK]	65.55	0.839	39.9	5.47
F1NMF6	Procollagen-lysine,2-oxoglutarate 5-dioxygenase 1 OS=Gallus gallus GN=PLOD1 PE=4 SV=1 - [F1NMF6_CHICK]	198.96	0.837	84.3	6.74
F1P2F0	Collagen alpha-3(VI) chain OS=Gallus gallus GN=COL6A3 PE=4 SV=1 - [F1P2F0_CHICK]	642.87	0.836	339.4	6.68
R4GFM0	FERM, RhoGEF and pleckstrin domain-containing protein 1 OS=Gallus gallus GN=FARP1 PE=4 SV=1 -	0.00	0.835	119.5	8.15

	[R4GFM0_CHICK]				
F1N8G4	Diphthamide biosynthesis protein 2 OS=Gallus gallus GN=DPH2 PE=4 SV=1 - [F1N8G4_CHICK]	0.00	0.834	52.1	5.54
H9L0H3	Alpha-actinin-4 (Fragment) OS=Gallus gallus GN=ACTN4 PE=4 SV=2 - [H9L0H3_CHICK]	515.20	0.834	71.6	6.09
Q5F4B1	Phosphoglycolate phosphatase OS=Gallus gallus GN=PGP PE=2 SV=1 - [PGP_CHICK]	39.98	0.833	33.0	5.73
P56673	Pituitary homeobox 1 OS=Gallus gallus GN=PITX1 PE=2 SV=1 - [PITX1_CHICK]	20.32	0.833	34.5	9.11
Q90611	72 kDa type IV collagenase OS=Gallus gallus GN=MMP2 PE=1 SV=1 - [MMP2_CHICK]	267.53	0.829	74.9	5.49
F1NME2	Integrin beta OS=Gallus gallus GN=ITGB5 PE=3 SV=1 - [F1NME2_CHICK]	51.04	0.828	88.4	6.71
F1NBZ7	Serine/threonine-protein phosphatase OS=Gallus gallus GN=PPP3CA PE=3 SV=2 - [F1NBZ7_CHICK]	0.00	0.827	60.6	5.83
P51890	Lumican OS=Gallus gallus GN=LUM PE=1 SV=1 - [LUM_CHICK]	44.33	0.826	38.6	6.52
B3TZC1	PNPLA7 OS=Gallus gallus GN=PNPLA7 PE=2 SV=1 - [B3TZC1_CHICK]	0.00	0.821	147.6	7.17
Q5ZLG0	Acetoacetyl-CoA synthetase OS=Gallus gallus GN=AACS PE=2 SV=1 - [AACS_CHICK]	189.47	0.818	74.3	6.49
F1NLD4	Inhibitor of nuclear factor kappa-B kinase subunit alpha OS=Gallus gallus GN=CHUK PE=4 SV=1 - [F1NLD4_CHICK]	0.00	0.817	86.1	6.32
F1NFE0	Collagen alpha-1(VI) chain OS=Gallus gallus GN=COL6A1 PE=4 SV=2 - [F1NFE0_CHICK]	110.52	0.812	107.9	5.90
F1N9N4	Stathmin-3 OS=Gallus gallus GN=NPC2 PE=4 SV=1 - [F1N9N4_CHICK]	34.96	0.808	16.2	6.51

F1NPX5	SH3 domain-binding glutamic acid-rich-like protein (Fragment) OS=Gallus gallus GN=SH3BGRL PE=3 SV=2 - [F1NPX5_CHICK]	106.54	0.802	12.9	4.88
P01038	Cystatin OS=Gallus gallus PE=1 SV=2 - [CYT_CHICK]	39.09	0.799	15.3	7.69
Q8QG94	Suppressor of fused OS=Gallus gallus GN=SUFU PE=2 SV=1 - [Q8QG94_CHICK]	46.78	0.786	53.7	5.33
Q90Y35-2	Isoform 2 of Zinc finger protein 622 OS=Gallus gallus GN=ZNF622 - [ZN622_CHICK]	0.00	0.783	42.6	6.65
F1NMZ3	Hemoglobin subunit epsilon OS=Gallus gallus GN=HBE PE=3 SV=1 - [F1NMZ3_CHICK]	37.75	0.761	16.6	8.91
Q5ZK77	Biogenesis of lysosome-related organelles complex 1 subunit 5 OS=Gallus gallus GN=BLOC1S5 PE=2 SV=1 - [BL1S5_CHICK]	34.23	0.759	22.6	7.06
F1P0D2	Glutamine synthetase (Fragment) OS=Gallus gallus GN=LOC417253 PE=3 SV=2 - [F1P0D2_CHICK]	0.00	0.758	44.9	7.02
F1NJT4	Fibronectin OS=Gallus gallus GN=FN1 PE=4 SV=2 - [F1NJT4_CHICK]	1373.06	0.752	259.0	6.11
Q155F6	Tumor necrosis factor-inducible protein 6 OS=Gallus gallus GN=TNFIP6 PE=2 SV=1 - [Q155F6_CHICK]	44.59	0.747	30.7	6.02
F1NJT3	Fibronectin OS=Gallus gallus GN=FN1 PE=4 SV=1 - [F1NJT3_CHICK]	1373.06	0.727	273.1	5.64
O93532	Keratin, type II cytoskeletal cochlear OS=Gallus gallus PE=2 SV=1 - [K2CO_CHICK]	95.26	0.693	53.8	6.10

Fold change=infected/control. Fold change >1 indicates up regulation, and fold change <1 indicates down regulation.
